# Single-trait and multi-trait genome-wide association analyses identify novel loci for blood pressure in African-ancestry populations

**DOI:** 10.1371/journal.pgen.1006728

**Published:** 2017-05-12

**Authors:** Jingjing Liang, Thu H. Le, Digna R. Velez Edwards, Bamidele O. Tayo, Kyle J. Gaulton, Jennifer A. Smith, Yingchang Lu, Richard A. Jensen, Guanjie Chen, Lisa R. Yanek, Karen Schwander, Salman M. Tajuddin, Tamar Sofer, Wonji Kim, James Kayima, Colin A. McKenzie, Ervin Fox, Michael A. Nalls, J. Hunter Young, Yan V. Sun, Jacqueline M. Lane, Sylvia Cechova, Jie Zhou, Hua Tang, Myriam Fornage, Solomon K. Musani, Heming Wang, Juyoung Lee, Adebowale Adeyemo, Albert W. Dreisbach, Terrence Forrester, Pei-Lun Chu, Anne Cappola, Michele K. Evans, Alanna C. Morrison, Lisa W. Martin, Kerri L. Wiggins, Qin Hui, Wei Zhao, Rebecca D. Jackson, Erin B. Ware, Jessica D. Faul, Alex P. Reiner, Michael Bray, Joshua C. Denny, Thomas H. Mosley, Walter Palmas, Xiuqing Guo, George J. Papanicolaou, Alan D. Penman, Joseph F. Polak, Kenneth Rice, Ken D. Taylor, Eric Boerwinkle, Erwin P. Bottinger, Kiang Liu, Neil Risch, Steven C. Hunt, Charles Kooperberg, Alan B. Zonderman, Cathy C. Laurie, Diane M. Becker, Jianwen Cai, Ruth J. F. Loos, Bruce M. Psaty, David R. Weir, Sharon L. R. Kardia, Donna K. Arnett, Sungho Won, Todd L. Edwards, Susan Redline, Richard S. Cooper, D. C. Rao, Jerome I. Rotter, Charles Rotimi, Daniel Levy, Aravinda Chakravarti, Xiaofeng Zhu, Nora Franceschini

**Affiliations:** 1 Department of Epidemiology & Biostatistics, School of Medicine, Case Western Reserve University, Cleveland, OH, United States of America; 2 Department of Medicine, Division of Nephrology, University of Virginia, Charlottesville, Virginia, United States of America; 3 Department of Obstetrics and Gynecology, Institute for Medicine and Public Health, Vanderbilt Genetics Institute, Vanderbilt University Medical Center, Nashville, Tennessee, United States of America; 4 Department of Public Health Sciences, Loyola University Chicago Stritch School of Medicine, Maywood, Illinois, United States of America; 5 Department of Pediatrics, University of California San Diego, La Jolla, California, United States of America; 6 Department of Epidemiology, School of Public Health, University of Michigan, Ann Arbor, Michigan, United States of America; 7 The Charles Bronfman Institute for Personalized Medicine, Icahn School of Medicine at Mount Sinai, New York City, New York, United States of America; 8 The Genetics of Obesity and Related Metabolic Traits Program, Ichan School of Medicine at Mount Sinai, New York City, New York, United States of America; 9 Division of Epidemiology, Department of Medicine, Vanderbilt-Ingram Cancer Center, Vanderbilt Epidemiology Center, Vanderbilt University School of Medicine, Nashville, Tennessee, United States of America; 10 Cardiovascular Health Research Unit, Department of Medicine, University of Washington, Seattle, Washington, United States of America; 11 Center for Research on Genomics and Global Health, National Human Genome Research Institute, National Institutes of Health, Bethesda, Maryland, United States of America; 12 Department of Medicine, Johns Hopkins University School of Medicine, Baltimore, Maryland, United States of America; 13 Division of Biostatistics, School of Medicine, Washington University in St. Louis, St. Louis, Missouri, United States of America; 14 Laboratory of Epidemiology and Population Sciences, National Institute on Aging, National Institutes of Health, Baltimore, Maryland, United States of America; 15 Department of Biostatistics, University of Washington, Seattle, Washington, United States of America; 16 Interdisciplinary Program of Bioinformatics, Seoul National University, Seoul, Republic of Korea; 17 Division of Adult Cardiology, Uganda Heart Institute, Makerere University College of Health Sciences, Kampala, Uganda; 18 Department of Medicine, Makerere University College of Health Sciences, Kampala, Uganda; 19 Tropical Metabolism Research Unit, Caribbean Institute for Health Research, University of the West Indies, Mona, Jamaica; 20 Department of Preventive Medicine, University of Mississippi Medical Center, Jackson, Mississippi, United States of America; 21 Data Tecnica International, Glen Echo, MD, United States of America and Laboratory of Neurogenetics, National Institute on Aging, National Institute of Health, Bethesda, Maryland, United States of America; 22 Department of Epidemiology, Rollins School of Public Health, Emory University, Atlanta, Georgia, United States of America; 23 Center for Genomic Medicine, Massachusetts General Hospital, Boston, Massachusetts, United States of America; 24 Anesthesia, Critical Care and Pain Medicine, Massachusetts General Hospital and Harvard Medical School, Boston, Massachusetts, United States of America; 25 Program in Medical and Population Genetics, Broad Institute, Cambridge, Massachusetts, United States of America; 26 Department of Genetics, Stanford University School of Medicine, Stanford, California, United States of America; 27 Institute of Molecular Medicine and Human Genetics Center, University of Texas Health Science Center at Houston, Houston, TX, United States of America; 28 Division of Structural and Functional Genomics, Center for Genome Science, Korea National Institute of Health, Cheongju, Republic of Korea; 29 Department of Internal Medicine, Graduate Institute of Biomedical and Pharmaceutical Science, College of Medicine, Fu Jen Catholic University, New Taipei City, Taiwan; 30 Division of Endocrinology, Diabetes, and Metabolism, Perelman School of Medicine at the University of Pennsylvania, Philadelphia, United States of America; 31 Human Genetics Center, School of Public Health, University of Texas Health Science Center, Houston, Texas, United States of America; 32 The George Washington University School of Medicine and Health Sciences, Washington DC. United States of America; 33 Department of Internal Medicine, Ohio State University, Columbus, Ohio, United States of America; 34 Survey Research Center, Institute for Social Research, University of Michigan Ann Arbor, Michigan, United States of America; 35 Division of Public Health Sciences, Fred Hutchinson Cancer Research Center, Seattle, Washington, United States of America; 36 Department of Biomedical Informatics, Department of Medicine, Vanderbilt University Medical Center, Nashville, Tennessee, United States of America; 37 Department of Medicine, Columbia University, New York City, New York, United States of America; 38 Medical Genetics Institute, Cedars-Sinai Medical Center, Los Angeles, CA, United States of America; 39 Division of Cardiovascular Sciences, National Heart, Lung, and Blood Institute, National Institutes of Health, Bethesda, Maryland, United States of America; 40 Tufts Medical Center, Tufts University School of Medicine, Boston, Massachusetts, United States of America; 41 Institute for Translational Genomics and Population Sciences, Los Angeles Biomedical Research Institute and Department of Pediatrics, Harbor-UCLA Medical Center, Torrance, CA; 42 Department of Preventive Medicine, Northwestern University Medical School, Chicago, Illinois, United States of America; 43 Institute for Human Genetics, University of California, San Francisco, California, United States of America; 44 Cardiovascular Genetics, University of Utah, Salt Lake City, Utah, United States of America; 45 Department of Biostatistics, Gillings School of Global Public Health, University of North Carolina, Chapel Hill, NC, United States of America; 46 The Mindich Child Health and Development Institute, Ichan School of Medicine at Mount Sinai, New York City, New York, United States of America; 47 Kaiser Permanente Washington Health Research Institute, Seattle, Washington, United States of America; 48 University of Kentucky, College of Public Health, Lexington, KY; 49 Department of Public Health Science, Seoul National University, Seoul, Republic of Korea; 50 Division of Epidemiology, Department of Medicine, Institute of Medicine and Public Health, Vanderbilt Genetics Institute, Vanderbilit University Medical Center, Nashville, Tennessee, United States of America; 51 Department of Medicine, Brigham and Women's Hospital, Harvard Medical School, Boston, Massachusetts, United States of America; 52 Population Sciences Branch, National Heart, Lung, and Blood Institute of the National Institutes of Health, Bethesda, MD, and the Framingham Heart Study, Framingham, Massachusetts, United States of America; 53 McKusick-Nathans Institute of Genetic Medicine, Johns Hopkins University School of Medicine, Baltimore, Maryland, United States of America; 54 Epidemiology, Gilling School of Global Public Health, University of North Carolina, Chapel Hill, North Carolina, United States of America; Georgia Institute of Technology, UNITED STATES

## Abstract

Hypertension is a leading cause of global disease, mortality, and disability. While individuals of African descent suffer a disproportionate burden of hypertension and its complications, they have been underrepresented in genetic studies. To identify novel susceptibility loci for blood pressure and hypertension in people of African ancestry, we performed both single and multiple-trait genome-wide association analyses. We analyzed 21 genome-wide association studies comprised of 31,968 individuals of African ancestry, and validated our results with additional 54,395 individuals from multi-ethnic studies. These analyses identified nine loci with eleven independent variants which reached genome-wide significance (P < 1.25×10^−8^) for either systolic and diastolic blood pressure, hypertension, or for combined traits. Single-trait analyses identified two loci (*TARID*/*TCF21* and *LLPH/TMBIM4*) and multiple-trait analyses identified one novel locus (*FRMD3)* for blood pressure. At these three loci, as well as at *GRP20/CDH17*, associated variants had alleles common only in African-ancestry populations. Functional annotation showed enrichment for genes expressed in immune and kidney cells, as well as in heart and vascular cells/tissues. Experiments driven by these findings and using angiotensin-II induced hypertension in mice showed altered kidney mRNA expression of six genes, suggesting their potential role in hypertension. Our study provides new evidence for genes related to hypertension susceptibility, and the need to study African-ancestry populations in order to identify biologic factors contributing to hypertension.

## Author summary

Hypertension is a global health problem which affects disproportionally people of African descent. We conducted a genome-wide association study of blood pressure in 31,968 Africans and African Americans to identify genes conferring susceptibility to increased blood pressure. This research identified three novel genomic regions associated with blood pressure which have not been previously reported in studies of other race/ethnicity. Using experimental models, we also showed an altered expression of these genes in kidney tissue in hypertension. These findings provide new evidence for genes influencing hypertension risk and supports the need to study diverse ancestry populations in order to identify biologic factors contributing to hypertension.

## Introduction

Genetic studies hold the promise of providing tools to better understand and treat clinical conditions. To achieve the clinical and public health goals of reducing hypertension and its sequelae, and to understand ethnic disparities in the risk for hypertension, there is a need to study susceptible populations for genetic determinants of blood pressure (BP). BP traits are highly heritable across world populations (30 to 55%).[1–4] Over 200 genetic loci have been identified in genome-wide association studies [5–13] and admixture mapping studies.[14–17] These variants explain approximately 3.5% of inter-individual variation in BP.[5, 7] However, there is still a paucity of studies focused on individuals of African descent. Most of the loci identified in the literature have not been replicated in individuals of African ancestry.[18, 19]

African Americans have higher mean BP, an earlier onset of hypertension, and a greater likelihood to have treatment-resistant hypertension than other ethnic groups.[20–23] Emerging research on Africans shows increasing prevalence of hypertension in urban African communities [24, 25] which are more Westernized than rural African communities and, so, more closely resemble communities in which African Americans live in the U.S. Hypertension contributes to a greater risk of coronary heart disease, stroke, and chronic kidney disease.[26–30] African Americans experience increased risk of these hypertension-related outcomes [31–34] but the underlying mechanisms, whether environmental exposures or increased genetic susceptibility, are unknown.

We hypothesized that additional variants associated with BP can be identified in people of African ancestry; some variants may be African-specific, as has been observed for multiple traits, including kidney disease [35] and metabolic syndrome.[36, 37] Other variants may be identified in novel loci based on a higher frequency of risk alleles in this population. We used high density imputed genotypes from the 1000 Genomes Project (1000G) to expand the genome coverage of genetic variants so that we could examine the evidence for association with BP traits.

Here, we report three novel loci associated with BP which are driven by variants that are common in or unique to African-ancestry populations. Through bioinformatics and experimental evidence of kidney gene expression in mice submitted to angiotensin-II (Ang II) induced hypertension, we provide evidence for a key role of these genes in the pathogenesis of hypertension. In addition, our study extends the discovery of BP loci to genes related to kidney and the immune systems, and provides biological relevance for these loci to BP regulation.

## Results

**Fig 1:**
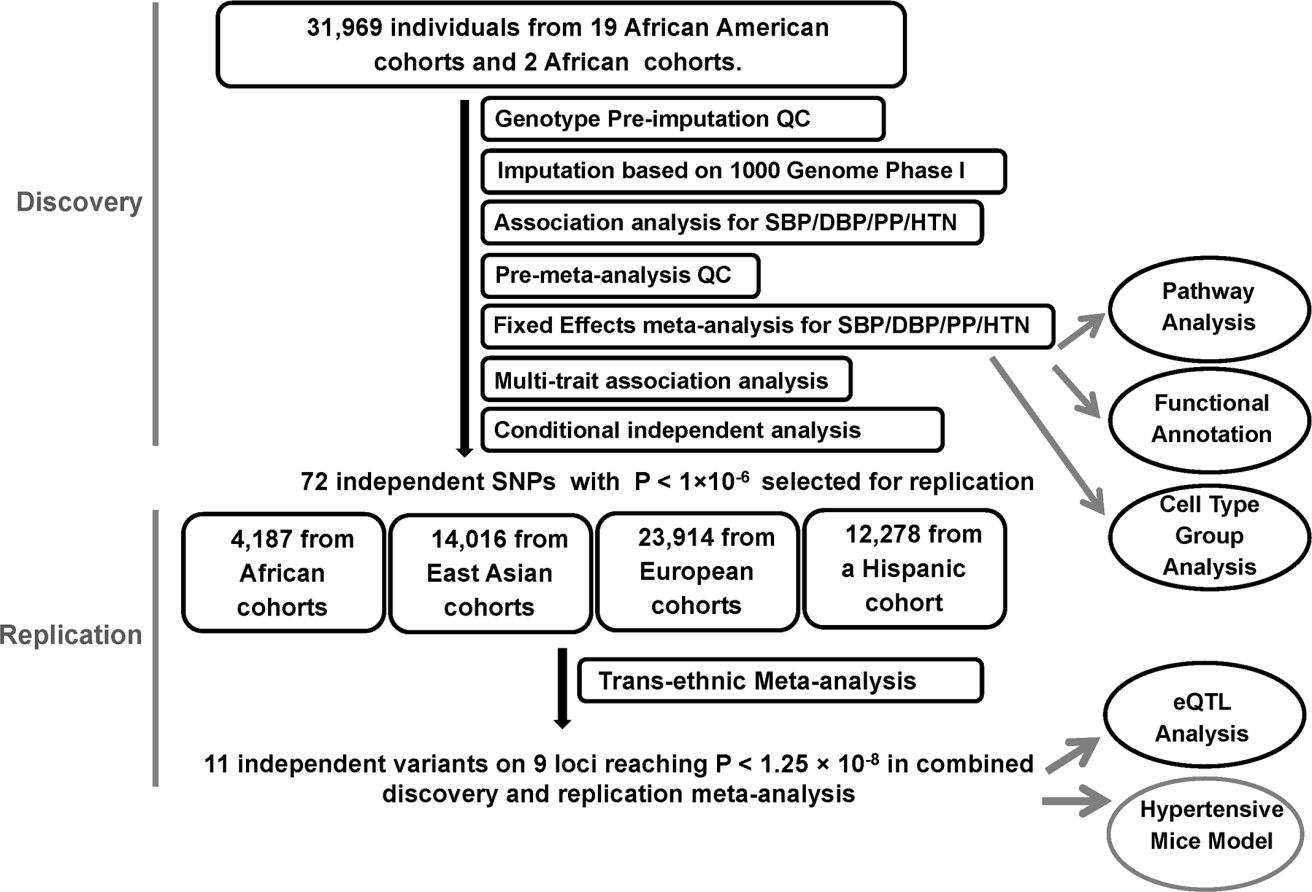
Study design schematic for discovery and replication of loci. QC, quality control; SBP, systolic blood pressure; DBP, diastolic blood pressure; PP, pulse pressure; HTN, hypertension; eQTL, expression quantitative loci.

The study design and analysis process are shown in Fig 1. Study characteristics, genotyping, and quality control (QC) for discovery and replication samples are shown in S1 and S2 Tables. The discovery samples included 31,968 individuals of African ancestry from 19 studies. The replication samples included 4,184 individuals of African ancestry from three studies, 23,914 individuals of European ancestry from five studies, 14,016 individuals of Korean ancestry from three studies, and 12,278 individuals of Hispanic/Latino ancestry from one study.

### Single-trait and multi-trait meta-analysis genome-wide association study (GWAS) results

Study-specific genomic-control inflation ranged from 0.98–1.06 (S3 Table, S1 Fig) and the linkage disequilibrium (LD) score regression intercepts of the single-trait BP meta-analyses calculated by the LD score regression approach ranged from 1.02–1.04. [38] These results suggest well-controlled population stratification.

The single-trait BP meta-analyses identified several genome-wide significant single nucleotide polymorphisms (SNP) at eight loci (P < 5.0×10^−8^, systolic BP (SBP): three loci, four SNPs; diastolic BP (DBP): three loci, three SNPs; pulse pressure (PP): three loci, four SNPs; and hypertension (HTN): one locus, one SNP), with the *EVX1/HOXA* locus identified for SBP, DBP and HTN (S2A–S2D Fig). When combining summary statistics for SBP, DBP, and HTN using the multi-trait approach CPASSOC,[39] we identified one locus by the multi-trait statistic S_Hom_ (*EVX1/HOXA*) and six loci by S_Het_ (*ULK4*, *TCF21*, *EVX1/HOXA*, *IGFBP3*, *CDH17*, *ZNF746*) at P < 5×10^−8^ (S2E and S2F Fig). Note some loci overlap between single-trait and multi-trait findings.

We observed 264 variants with P < 1×10^−6^ for either single- or multi- trait GWAS and these variants were further analyzed by conditional association on the most associated SNPs at each locus (S4 Table). These analyses resulted in 72 independent associations, which included 58 SNPs with minor allele frequency (MAF) ≥ 0.05 and 14 with low frequency variants (0.01< MAF < 0.05) (S5 Table).

### Trans-ethnic replication

**Fig 2:**
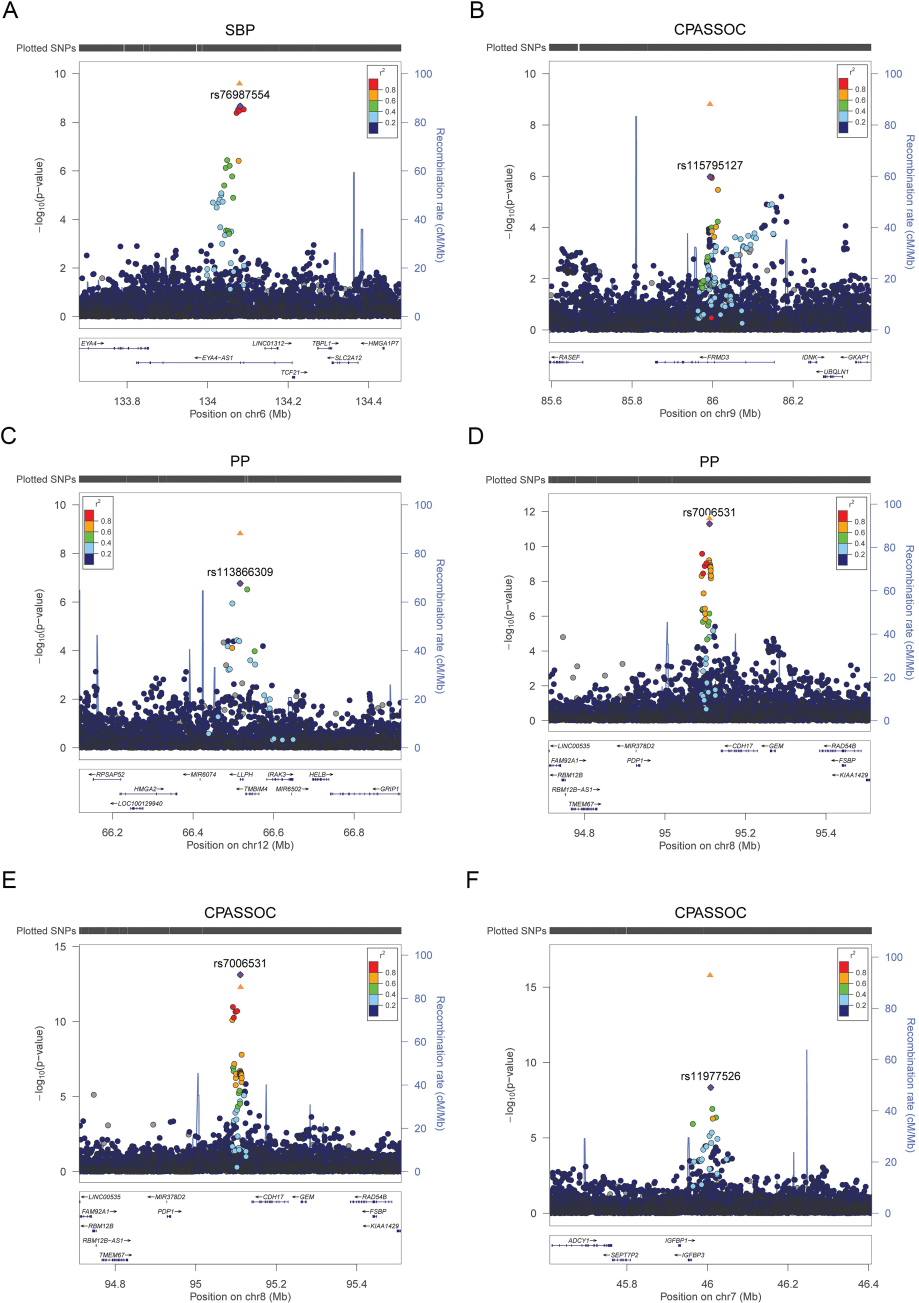
Regional plots of the significant loci **A.** *TARID/TCF21* for SBP B. *FRMD3* for S_Het_ of CPASSOC C. *LLPH* locus for PP **D.** *CDH17* for PP **E.** *CDH17* for S_Het_ of CPASSOC F. *IGFBP3* for S_Het_ of CPASSOC. The *y* axis shows the −log_10_ P values of SNP associations, and the *x* axis shows their chromosomal positions. The lowest P value SNP is plotted as a purple diamond and its correlation with other SNPs in the region is shown in color. The orange triangle is P value in the combined discovery and replication trans-ethnic meta-analysis of the lowest P value SNP.

Among these 72 variants carried forward for trans-ethnic replication, nine variants, all low frequency variants (MAF<0.02), were not available in replication cohorts because they were either monomorphic in the replication population or had a low imputation quality, reducing our replication effort to 63 variants (S6 Table). Eleven independent variants at nine loci were significantly associated with BP traits at P < 1.25×10^−8^ in the combined discovery and replication analyses and are reported in Table 1. This significance level was determined by adjusting for two independent traits for SBP, DBP, PP and HTN, and two tests of multiple trait analysis. This includes six variants that reached significance level at discovery stage (P <5 x10^-8^). Two loci were identified only through multi-trait analyses (*FRMD3*, *IGFBP3*). Three of these nine loci are novel: *TARID* /*TCF21*, *FRMD3*, and *LLPH/TMBIM4* (Fig 2A–2C). Four loci (*ULK4*, *PLEKHG1*, *EVX1/HOXA* cluster, and *GPR20*) have been reported in our previous BP GWAS of African ancestry (S3 Fig),[7, 18] and two loci (*IGFBP3*, *CDH17*) have been reported in multiple-trait analyses of African-ancestry studies (Fig 2D–2F).[39] A composite genetic-risk score using the eleven variants identified accounted for 1.89%, 2.92%, 1.03% and 1.08% of the variance for SBP, DBP, PP and HTN respectively.

**Table 1:** Loci identified in combined COGENT-BP African ancestry discovery samples and multi-ethnic replication samples.

**SNP**	**Effect Allele/** **Other Allele**	**Chr**	**Nearby Gene**	**COGENT-BP Allele Frequency**	**1000G Phase 1 Allele Frequency**				**Single or Multi-Trait (CPASSOC) Statistic**	**COGENT-BP Discovery (Up to N = 31,155)**		**Trans-Ethnic Replication (Up to N = 54,245)**	**Combined Meta-analyses** **(Up to N = 85,397)**
					AFR	AMR	ASN	EUR		Beta (SE)	P	P	P
**SNPs in novel loci**													
**rs76987554**	C/T	6	*TARID/* *TCF21*	0.91	0.91	0.99	1	1	SBP	1.85 (0.31)	**2.2x10**^**-9**^	2.0x10^-2^	**2.3x10**^**-10**^
**rs115795127**	T/C	9	*FRMD3*	0.89	0.86	1	1	1	CPASSOC S_Het_	NA	1.1x10^-6^	8.4x10^-6^	**7.3x10**^**-9**^
**rs113866309**	C/T	12	*LLPH/* *TMBIM4*	0.02	0.02	0.01	0.00	0.00	PP	3.28 (0.63)	1.7x10^-7^	1.5x10^-3^	**8.2x10**^**-9**^
**SNPs in published BP loci**													
**rs7651190**	G/A	3	*ULK4*	0.65	0.72	0.17	0.15	0.19	DBP	0.45 (0.11)	4.2x10^-5^	1.0x10^-5^	**2.0x10**^**-9**^
									CPASSOC S_Het_	NA	**6.9x10**^**-9**^	2.0x10^-4^	**9.8x10**^**-11**^
**rs7372217**	G/A	3	*ULK4*	0.66	0.71	0.20	0.16	0.20	DBP	0.55 (0.11)	9.5x10^-7^	8.1x10^-7^	**5.3x10**^**-12**^
									CPASSOC S_Het_	NA	8.2x10^-6^	6.5x10^-8^	**1.4x10**^**-11**^
**rs62434120**	T/A	6	*PLEKHG1*	0.85	0.83	0.82	0.95	0.92	SBP	1.19 (0.24)	1.1x10^-6^	2.7x10^-3^	**5.7x10**^**-9**^
**rs11563582**	A/G	7	*EVX1/* *HOXA* cluster						SBP	1.61 (0.28)	**7.1x10**^**-9**^	4.2x10^-4^	**4.5x10**^**-10**^
				0.13	0.16	0.09	0.05	0.08	DBP	1.02 (0.17)	**8.4x10**^**-10**^	1.4x10^-4^	**1.7x10**^**-11**^
									CPASSOC S_Hom_	NA	**1.5x10**^**-10**^	8.0x10^-4^	**1.9x10**^**-11**^
									CPASSOC S_Het_	NA	**1.1x10**^**-9**^	9.4x10^-3^	**1.8x10**^**-9**^
**rs6969780**	C/G	7	*HOXA*						SBP	0.82 (0.19)	1.7x10^-5^	6.5x10^-5^	**6.2x10**^**-9**^
				0.30	0.35	0.21	0.13	0.10	DBP	0.62 (0.12)	7.0x10^-8^	2.1x10^-4^	**3.3x10**^**-10**^
									CPASSOC S_Hom_	NA	4.1x10^-7^	4.0x10^-4^	**9.9x10**^**-9**^
**rs11977526**	A/G	7	*IGFBP3*	0.34	0.34	0.31	0.78	0.41	CPASSOC S_Het_	NA	**4.5x10**^**-9**^	2.9x10^-9^	**7.3x10**^**-16**^
**rs7006531**	G/A	8	*CDH17*	0.15	0.19	0.02	0.00	0.00	PP	1.16 (0.17)	**5.0x10**^**-12**^	9.7x10^-2^	**5.9x10**^**-12**^
									CPASSOC S_Het_	NA	**7.6x10**^**-14**^	6.1x10^-3^	**2.2x10**^**-13**^
**rs78192203**	T/A	8	*GPR20*	0.80	0.79	0.98	1	1	DBP	0.77 (0.14)	**1.3x10**^**-8**^	2.7x10^-4^	**4.1x10**^**-11**^

Bold P-values represent either significance level at 5.0x10^-8^ in discovery sample or at 1.25x10^-8^ at combined discovery and replication samples. 1000G samples: AFR, African ancestry; AMR, American ancestry; ASN, Asian ancestry; EUR, European ancestry

### Newly identified loci harbor variants common only in African-ancestry populations

Five of the eleven replicated variants are common in individuals of African ancestry but rare or monomorphic in individuals of non-African ancestry (rs76987554, rs115795127, rs113866309, rs7006531, and rs78192203)(Table 1). These five variants were 1) either low frequency or common variants in COGENT-BP African-ancestry samples; 2) low frequency in 1000G Phase I Integrated Release Ad Mixed-American ancestry (AMR); and 3) monomorphic in 1000G Asian ancestry (ASN) or European ancestry (EUR). One common variant was present in only 1000G samples of African ancestry (rs115795127 at *FRMD3*, Table 1). These variants were located at the three novel loci (*TARID*/*TCF21*, *FRMD3*, and *LLPH/TMBIM4*). Given the differences in allele frequency across continental-ancestry populations, we examined the evidence for selection at each of these loci using iHS, which measures the amount of extended haplotype homozygosity at a given SNP along the ancestral allele relative to the derived allele.[40] The iHS score for rs115795127 was 2.7 in African American samples from the Candidate-gene Association Resource (CARe) consortium (see Methods), suggesting selection at the *FRMD3* locus (S7 Table).

### Distinct associations at *EVX1/HOXA*, *ULK4*, and *GPR20* in African-ancestry populations

We observed two independent genome-wide significant variants at the *EVX1/HOXA* locus (P < 1.25×10^−8^). The two variants, rs11563582 and rs6969780, are in weak LD (r^2^ = 0.21) (S3A–S3C Fig), and the LD pattern suggests that these SNPs are located in two blocks (S4 Fig). SNP rs11563582 is in strong LD with the previously reported SNP in the region (rs17428741).[18] SNP rs6969780 remained significant when conditioning on rs11563582 (S4 Table), thus demonstrating the presence of allelic heterogeneity at this locus. Two independent variants at *ULK4* reached the significance threshold: rs7651190 and rs7372217 (LD r^2^ = 0.15) (S4E Fig). SNP rs7372217 is in strong LD with the previous reported SNP rs1717027.[18] The association evidence of rs1717027 can be explained by rs7372217 but not by rs7651190 in conditional analysis (S4 Table). Thus, rs7651190 is an independent association at this locus. At the *GPR20* locus, our most significant SNP, rs78192203, is 8kb away and it is not in LD with the published SNP, rs34591516 (r^2^ = 0.008, D^’^ = 0.68 in African American CARe participants).

## Pathway analyses suggest enrichment of immune pathways for BP traits

To gain insight into biologic mechanisms underlying genes associated with BP traits, we performed pathway analysis using publicly available databases. [41] The most relevant pathways identified were GSK3, Th1/Th2 differentiation, and Sonic Hedgehog (SHH) pathways (BIOCARTA): pyrimidine metabolism, apoptosis signaling pathway, and B cell activation (Panther); JAK Stat signaling, T cell receptor signaling, and B cell receptor signaling (Ingenuity); cytokine-cytokine receptor interaction and vascular smooth muscle contraction (KEGG); and neuronal activity, T cell mediated immunity, and tumor suppressor (Panther Biological Process) (Gene Set Enrichment Analysis [GSEA] P-value < 0.01, S8 Table). These analyses suggest enrichment of immune pathways for BP traits.

### Tissue and cell type group enrichment analyses identify immune, kidney, and cardiovascular enriched systems

**Fig 3:**
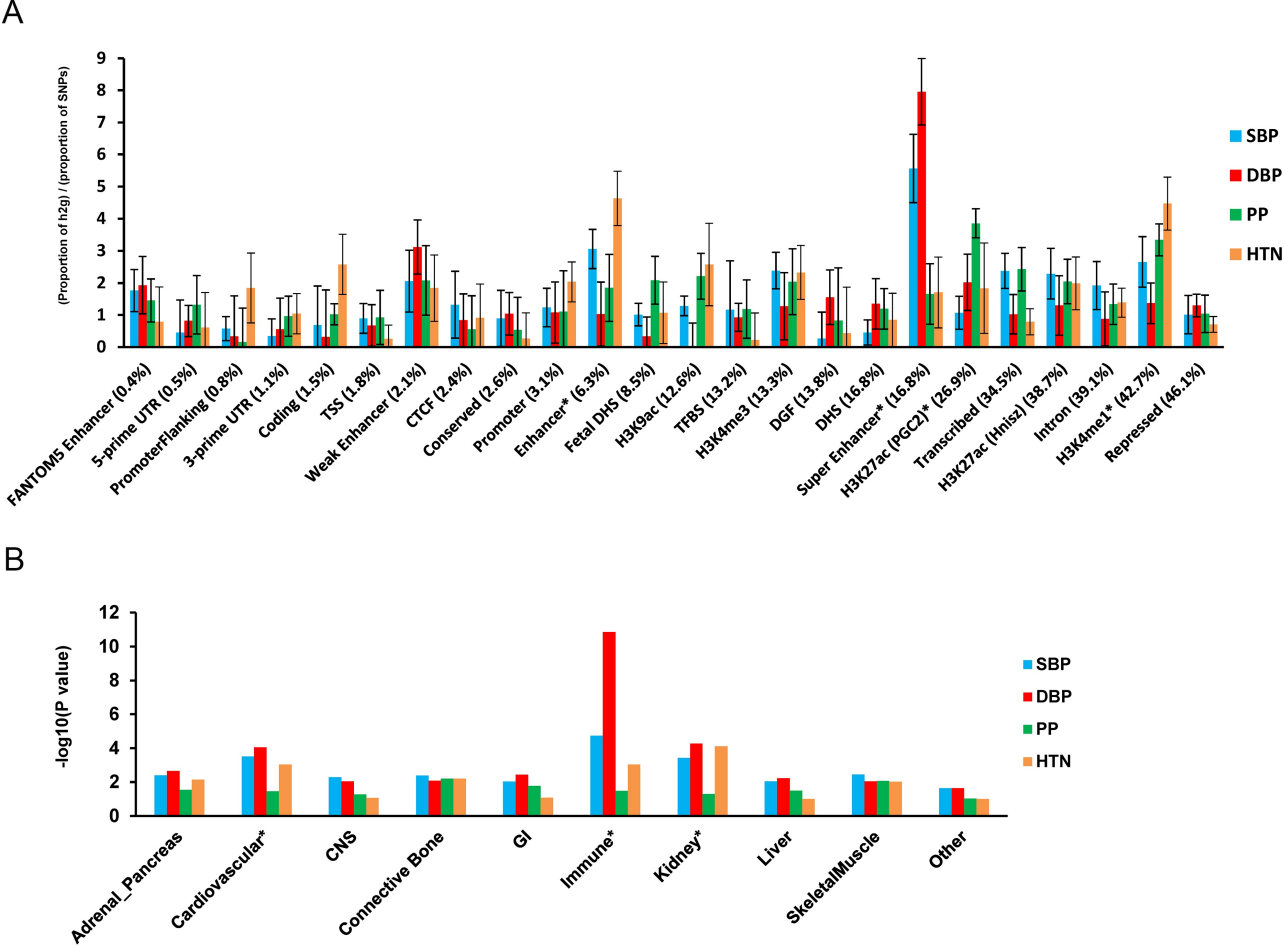
Enrichment for functional annotations and cell-type groups using stratified LD score regression. **A.** Enrichment estimates of 24 main annotations for each of four BP traits. Annotations are ordered by size. Error bars represent jackknife standard errors around the estimates of enrichment, and stars indicate significance at P < 0.05 after Bonferroni correction for 24 hypotheses tested and four BP traits. **B.** Significance of enrichment of 10 cell-type groups for four BP traits. Dotted line and stars indicate significance at P < 0.05 after Bonferroni correction for 10 hypotheses tested and four BP traits.

We performed functional annotation and cell type group enrichment analysis using the stratified LD score regression approach which uses data from ENCODE and the Roadmap Epigenetic Project, as well as GWAS results while accounting for the correlation among markers. [42] We estimated functional categories of enrichment using an enrichment score, which is the proportion of SNP-heritability in the category divided by the proportion of SNPs. We identified super enhancer (P_Enrich_ = 5.4×10^−5^_,_ Enrichment = 5.6 for DBP), enhancer (P_Enrich_ = 4.8 ×10^−4^, Enrichment = 4.3 for HTN), and H3K27ac (P_Enrich_ = 3.2×10^−4^, Enrichment = 3.6 for HTN) significant enrichment (Fig 3). These results support a role of identified noncoding regulatory regions in BP regulation. In addition, the following cell types showed significant enrichment (P ≤ 2.5 × 10^−3^): the immune (P_Enrich_ = 1.4×10^−9^, Enrichment = 8.4 for DBP), kidney (P_Enrich_ = 5.4×10^−5^, Enrichment = 4.8 for DBP), and cardiovascular (P_Enrich_ = 8.9×10^−5^, Enrichment = 4.2 for SBP) systems (Fig 3).

We next determined the enrichment of variants at the eleven genome-wide significant loci for DNase l hypersensitive (DHS) sites in 34 tissue categories from ENCODE. At each locus, we identified variants in r^2^>0.1 with the index variant and calculated causal evidence (Bayes Factors) for each variant. We then tested for enrichment in the causal evidence of variants in DHS sites using fGWAS.[43] We found enrichment of blood/immune DHS (Enrichment = 3.1) and cardiovascular DHS (blood vessel Enrichment = 28.7, heart Enrichment = 2.0), in addition to DHS in several fetal tissues (S5 Fig). Candidate causal variants at several loci overlapped enriched DHS sites. For example, at the *LLPH/TMBIM4* locus, the most likely causal variant, rs12426813, overlaps a DHS site active in immune (CD14+, CD4+, CD34+), blood vessel (HMVEC), and heart (HCF) cells (S5 Fig).

### Overlap with eQTL at specific tissues

To examine whether the eleven significant SNPs are eQTL, we searched the genotype-tissue expression (GTEx) pilot database, which includes non-disease human tissue.[42] Among the eleven SNPs, three SNPs have been identified as eQTL: rs6969780 (*HOXA2*), rs7651190 (*ULK4*), and rs62434120 (*PLEKHG1*) (S9 Table). SNP rs6969780 is an eQTL for expression of *HOXA2*, *HOXA7*, *HOTAIRM1*, and *HOXA5* in multiple tissues, including esophagus, artery, lung, skin, nerve, adipose, skeletal muscle, and stomach tissues. SNP rs7651190 is an eQTL for *ULK4* and *RPL36P20* in artery, whole blood, thyroid, nerve, esophagus, skeletal muscle, skin, brain, and stomach cells/tissues. SNP rs62434120 is an eQTL for *PLEKHG1* in testis tissue.

### Kidney gene expression in experimental angiogensin II-induced hypertension

**Fig 4:**
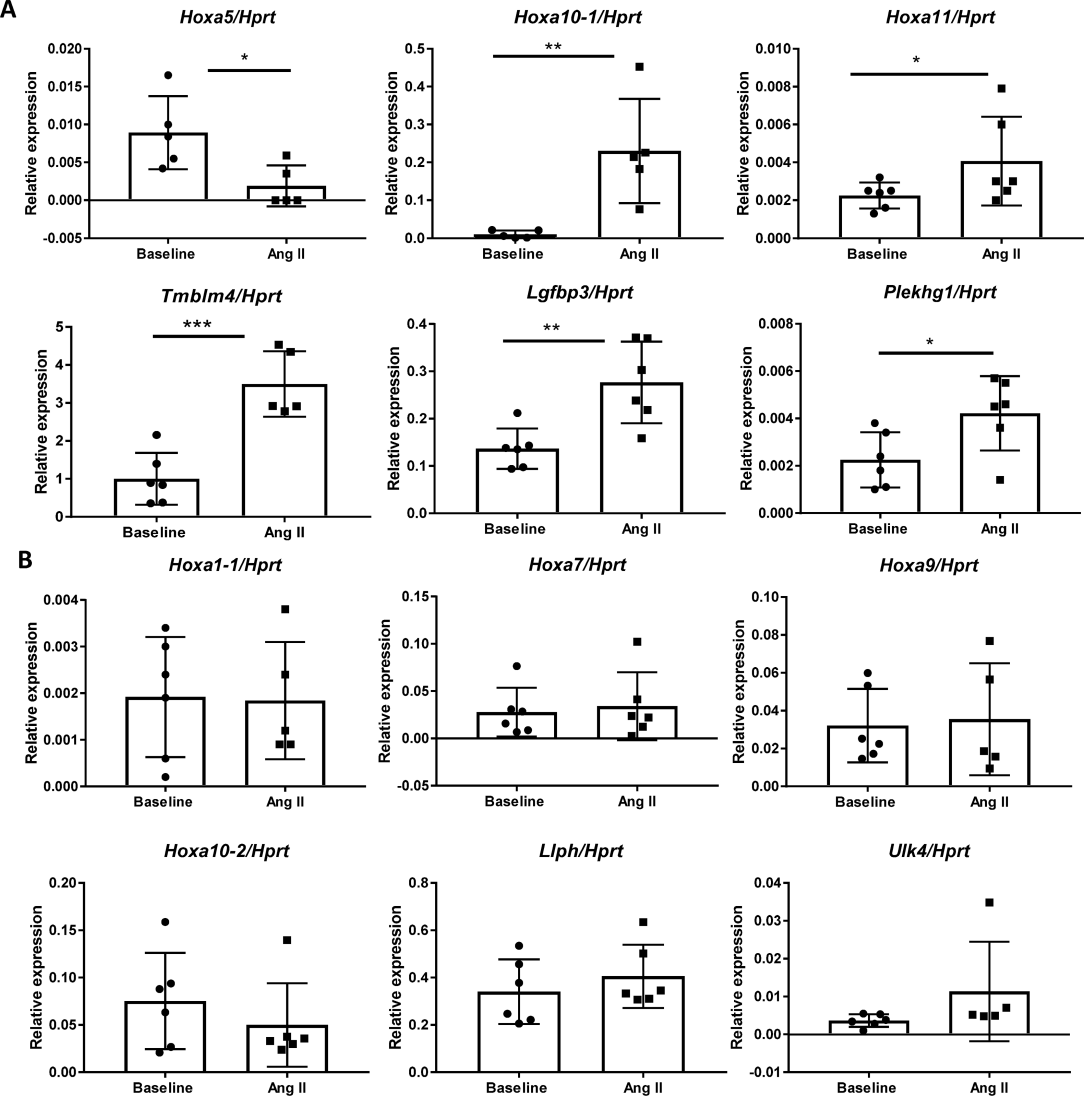
Relative renal mRNA levels of genes identified at baseline and after 2 weeks of Ang II-induced hypertension. *HPRT* gene was used for normalization. N ≥ 5 in each group. **A.** Genes that were differentially expressed between baseline and Ang II conditions. **B.** Genes that were not altered between the two conditions. * P < 0.05. ** P < 0.01. *** P < 0.001.

To determine if identified genes are functionally involved in BP regulation in the kidney during hypertension,[44] we quantified gene expression in mice kidneys at baseline and during the hypertensive state induced by Ang II. This hypertensive model was chosen for two reasons: 1) to mimic the low plasma renin state, albeit more exaggerated than the level observed, in African-ancestry individuals that has been suggested to reflect the elevated renin-angiotensin system activity at the tissue level in the kidney [45], and 2) maintenance of hypertension in the Ang II model requires activation of the immune system that is implicated in several identified loci.[46, 47] Kidney gene expressions of the identified genes were compared to age-matched untreated mice after two weeks of Ang II infusion, which increases SBP. For the *HOXA* locus, we examined the expression of genes that are known to be expressed in the mouse kidney: *Hoxa1* (2 isoforms), *5*, *7*, *9*, *10* (2 isoforms), and *11*. Among all the genes examined, *Tmbim4* was the most abundantly expressed gene in the kidney at baseline. Six genes—*Hoxa5*, *Hoxa10-1* isoform, *Hoxa11*, *Tmbim4*, *Igfbp3*, and *Plekhg1*—were significantly differentially expressed in the kidney after Ang II treatment compared to baseline (Fig 4). Except for *Hoxa5*, which showed a significant decrease (Fig 4A), the expression of all these genes increased after the intervention. The expression of six genes—*Hoxa1-1* isoform, *Hoxa7*, *Hoxa9*, *Hoxa10-2* isoform, *Llph*, and *Ulk4*—were unchanged after Ang II infusion (Fig 4B). The following genes were not expressed in the adult mouse kidney at baseline or after Ang II intervention: *Frmd3-1* isoform, *Frmd3-2* isoform, *Grp20*, *Tcf21*, *Cdh17*, and *Hoxa1-2* isoform.

## Discussion

To date, this is the largest genome-wide analysis of African-ancestry populations to study genetic variants underlying BP traits using dense-coverage imputed genotypes. Our main findings are eleven independent variants at nine loci, significantly associated with BP traits, including three newly identified loci (*TARID/TCF21*, *FRMD3*, *LLPH/TMBIM4*). We also found evidence for additional independent SNP associations in fine-mapping of three previously described loci, *ULK4*, *EVX1/HOXA*, and *GRP20*.[18, 39]

The most significant variants at *TARID/TCF21*, *FRMD3*, *GPR20*, and *CDH17* are common variants in COGENT-BP African-ancestry participants, but monomorphic or low frequency in non-African-ancestry populations. For example, rs115795127 at *FRMD3* is rare in European populations (MAF = 0.0007) and absent in East Asian and Hispanic/Latino populations. Therefore, they could not be identified in GWAS of non-African-ancestry populations even when increasing sample sizes. We also show evidence for selection for the variant at *FRMD3*, although additional studies should confirm these findings. The African-specific variants were not well tagged by HAPMAP2 data and therefore were not detected in our previous African-ancestry GWAS.[18] Overall, our results suggest additional gain in discovery when using dense imputed genotypes and support a role of population-specific alleles in African and African-admixed populations contributing to BP regulation and hypertension. Furthermore, they support the rationale and the need to study diverse populations in order to more effectively characterize the genetic architecture of BP in populations and the ethnic disparities in hypertension.

Functional annotation of our lead variants showed co-localization with annotated elements, including super enhancer, enhancer, and H3K27ac chromatic mapping in immune cells and kidney tissues, which has not been previously reported, in addition to cardiovascular tissues. There was also evidence for regulatory function in these relevant tissues through gene expression regulation (eQTL) and through overlaps with DHS in relevant tissues/cells. This evidence was additionally supported by experimental findings of differential expression of six genes (*Hoxa5*, *Hoxa10-1* isoform, *Hoxa11*, *Tmbim4*, *Igfbp3*, and *Plekhg1*) in the mouse kidney after HTN induced by Ang II treatment. Overall, our results suggest the functional importance of identified genes in regulating BP in both normal and hypertension states.

At the newly identified loci, SNP rs76987554 is an intronic variant in *TARID* (*TCF21* antisense RNA inducing promoter demethylation) which has not been previously reported to be associated with BP traits. A nearby gene, *TCF21* (transcription factor 21), is a transcription factor of the basic helix-loop-helix family, which is mainly expressed in the liver, kidney, and heart. *TCF21* is involved in epithelial differentiation and branching morphogenesis in kidney development,[48] and was associated with hypertension in a study of individuals of Japanese ancestry.[49] At the chromosome 7, rs115795127 is an intronic variant to *FRMD3* (FERM domain containing 3) which encodes a protein involved in maintaining cell shape and integrity. *FRMD3* has been associated with type 1 and type 2 diabetic kidney diseases in different ethnic populations, including those of European, African, and Asian ancestries.[50] The diabetes variant, rs10868025, is not in LD with rs115795127 in our African American samples or in 1000G EUR samples (r^2^ = 0.00028 and 0.0018, respectively), thus representing an independent association at this locus.

At chromosome 9, the functions of *LLPH* and *TMBIM4* genes in BP regulation are currently unknown. *LLPH* belongs to the learning-associated protein family and is highly expressed in the immune system and the adrenal gland. *TMBIM4* encodes the transmembrane BAX inhibitor motif-containing protein 4 and is highly expressed in whole blood, the immune system, and the adrenal gland.[51] The most significant variant at this locus, rs113866309, overlaps a DHS in immune, blood vessel, and heart cells. In our experimental model in mice, *Tmbim4* gene expression was significantly increased after Ang II-induced HTN. This gene has been shown to inhibit apoptosis[52] and to decrease the efficacy of inositol 1,4,5-triphosphate (IP_3_)-dependent release of intracellular Ca^2+^. [53] This raises the possibility that the TMBIM4 protein may serve to dampen the effect of Ang II, which activates IP_3_ in vascular smooth muscle cells through the stimulation of the angiotensin type 1 receptor.[51, 53, 54] Therefore, it is possible that in conditions of activated renin-angiotensin system, genetic variants that lower the expression of *TMBIM4* may augment BP, whereas genetic variants that increase its expression may attenuate BP.

Other genes, such as *Hoxa5*, *Hoxa10-1*, *Hoxa11*, *Igfbp3*, and *Plekhg1*, were significantly differentially expressed after Ang II-induced HTN in our mice experimental models. The *HOXA*-cluster has been identified in our previous GWAS of BP in African ancestry and in a recent GWAS of BP in European ancestry[5] though the underlying mechanisms related to BP control are unknown. We identified two independent variants at this locus; further studies are needed to delineate which of the *HOXA* genes are most likely involved in the association. In our experimental mice model, the *Hoxa10-1* isoform had a greater than 20-fold increase in kidney expression during Ang II-induced HTN compared to baseline levels. However, it remains to be determined whether it is an effect of Ang II in hypertension, or a compensatory response to hypertension. Future studies using genetic manipulation in rodents are required to determine whether these changes are specific response related to BP and Ang II or simply a generic response to stress.

We identified several additional pathways involved in BP traits, including the GSK3 pathway, which has been reported to influence Wnt-mediated central BP regulation.[55] The Th1/Th2 pathway is involved in the regulation of immune responses[56] and has been linked to hypertension and atherosclerosis.[57, 58] The role of the immune system in the development of hypertension has been suggested in clinical studies and experimental animal models.[59–64] This includes reports of overlap of genetic variant associations between BP traits and immune-disorders [65] and evidence of enrichment of immune pathways from GWAS of BP.[66] Mutations of *SH2B3*, a gene identified in a GWAS of hypertension, have been recently shown to attenuate Dahl salt-sensitivity hypertension through inflammatory modulation.[67] In addition, the actions of Ang II in the pathophysiology and maintenance of hypertension are in part mediated through the activation of the immune system.[46]

Our assessment of the clinical implications of identified variants is limited by available data on African-ancestry populations. For example, there are currently no large publicly available GWAS of coronary heart disease or stroke outcomes in African-ancestry populations. It should also be noted that most of our replication cohorts were from populations other than those of African ancestry. Therefore, the power of replication analysis could still be low, which explains why only 11 of 63 variants were successfully replicated.

In summary, we report 11 independent variants at nine loci that are potential regulators of BP in our African-ancestry population study. Three loci are new. Identified BP variants are enriched in immune, kidney, heart, and vascular system pathways. Our experimental findings suggest that several of these genes may be involved in the renin-angiotensin pathways in the kidney during hypertension. Further population studies and experimental models are required for a comprehensive assessment of the identified genes across the immune, kidney, and cardiovascular systems. Our study demonstrates the need to further study individuals of African ancestry in order to identify loci and new biological pathways for BP.

## Methods

### Samples and BP phenotypes

Each study followed protocols for phenotype harmonization. For individuals taking anti-hypertensive medications, we added 15 and 10 mm Hg to measured SBP and DBP, respectively, a standard method used in other BP GWAS.[6, 68] PP was calculated as the difference between SBP and DBP after addition of the constant values. HTN was defined by a SBP ≥ 140 mm Hg, a DBP ≥ 90 mm Hg, or use of antihypertensive drugs.[69]

### Genotyping and imputation

Each cohort was genotyped on either Affymetrix or Illumina genotyping platforms. Pre-imputation quality criteria were applied as described in S2 Table, and included exclusion of individuals with discordant self-reported gender and genetic gender. Imputation was performed using the software MACH-ADMIX, MACH-minimac or IMPUTE2 [70–72] using the Phase 1 integrated (March 2012 release) multi-ethnic reference panel from the 1000G Consortium (http://www.internationalgenome.org/).[73]

### Association analysis

Autosomal chromosome SNP associations for SBP, DBP, and PP were assessed by linear regression for unrelated data or by the generalized linear mixed-effects model for family data, under the assumption of an additive genetic model. All models were adjusted for age, age^2^, sex, and body mass index. Up to ten principal components were included, as needed as covariates in the regression models, to control population stratification.[74, 75] We used standardized pre-meta-analysis QC criteria for all 21 discovery studies.[76] At the SNP level, we excluded variants with 1) imputation quality r^2^ < 0.3 in MACH or <0.4 in IMPUTE2; 2) the number of informative individuals (2×MAF×N×r^2^) ≤ 30; 3) an effect allele frequency (EAF) difference larger than 0.3 in comparison with the mixture of 80% YRI and 20% CEU of 1000G; and 4) the absolute regression coefficient ≥ 10. SNPs that passed the QC were carried forward for inverse variance weighted meta-analyses, implemented in METAL.[77]

### Multi-trait statistical analyses using CPASSOC

We applied the CPASSOC software to combine association evidence of SBP, DBP, and HTN. CPASSOC provides two statistics, *S*_*Hom*_ and *S*_*Het*,_ as previously described.[39] *S*_*Hom*_ is similar to the fixed effect meta-analysis method[77] but accounts for the correlation of summary statistics of the multi-traits and for overlapping or related samples among the cohorts. *S*_*Hom*_ uses the trait sample size as the weight, so that it is possible to combine traits with different measurement scales. *S*_*Het*_ is an extension of *S*_*Hom*,_ and it can increase the statistical power over S_*Hom*_ when a variant affects only a subset of traits. The distribution of *S*_*Het*_ under the null hypothesis was obtained through an estimated beta distribution. To calculate the statistics, *S*_*Hom*_ and *S*_*Het*_, and to account for the correlation among the traits, a correlation matrix is required. In this study, we used the correlation matrix calculated from the residuals of the three BP traits after adjustments for covariates and principal components.

### Replication and meta-analyses

All independent SNPs identified with P < 10^−6^ (threshold chosen for suggestive association) in the discovery stage were carried forward for replication in African-ancestry individuals and in multi-ethnic samples of European Americans, East Asians, or Hispanics/Latinos (Fig 1). For single-trait analyses, we conducted fixed effect meta-analyses in the replication sets for each of four BP traits (SBP, DBP, PP and HTN), followed by a combined trans-ethnic meta-analysis of each trait. This was followed by a mega-meta-analyses, combining the results of discovery and replication for single traits using fixed-effects meta-analysis. We also performed a multi-trait CPASSOC analysis of SBP, DBP, and HTN in each replication study. Because CPASSOC only generated test statistics S_Hom_/S_Het_ and corresponding P values without effect sizes, we combined the association P values from all four replication populations using Fisher’s method (http://hal.case.edu/zhu-web/). Finally, we combined the CPASSOC meta-analysis results from the discovery and replication stages using Fisher’s method.

### Multiple-testing thresholds

For a single trait GWAS discovery analysis, we used genome-wide significant level P = 5.0×10^−8^. We performed six different analyses, four single trait (SBP, DBP, PP and HTN) analyses and two CPASSOC (S_Hom_ and S_Het_) analyses for each SNP. For the four single correlated traits (SBP, DBP, PP and HTN), we calculated the number of independent traits using the eigenvalues of the correlation matrix, [78] which resulted two independent traits. Therefore, we counted four independent analyses, which were two independent single traits and two statistics of CPASSOC analyses, and applied an experimental significance level P = 1.25×10^−8^ for claiming a genome-wide significance when combining discovery and replication samples. We should point out that the two CPASSOC test statistics and a single trait statistic are not independent. Thus, the significance level P = 1.25×10^−8^ is conservative.

### Conditional analysis

Since a locus may consist of multiple independent signals, we applied approximate conditional analysis implemented in GCTA-COJO[79, 80] using the summary statistics of SNPs with P < 1.0×10^−6^ from both of the individual trait meta-analyses (http://cnsgenomics.com/software/gcta/cojo.html). The LD among variants was estimated from the five African American cohorts from the CARe consortium.[79]

### Pathway analysis

Pathway analysis was performed using the Meta-Analysis Gene-set Enrichment of variant Associations (MAGENTA) program (http://www.broadinstitute.org/mpg/magenta/).[41] Using the summary statistics from the four BP traits and two statistics from CPASSOC, from the discovery stage, we tested whether sets of functionally-related genes are enriched for associations. This method first converts the P values of SNPs into gene scores with correcting for confounders, such as gene site, number of variants in a gene, and their LD patterns, and then calculated a gene set enrichment P value for each biological pathway or gene set of interest using a non-parametric statistical test. The nominal GSEA P value refers to the nominal gene set enrichment P value for a gene set. The database of pathway/gene-sets to be tested include Ingenuity (June 2008), KEGG (2010), GO, and the Panther, signaling pathways downloaded from MSigDB and PANTHER (http://www.broad.mit.edu/gsea/msigdb/collections.jsp; http://www.pantherdb.org/).[81] We applied the parameters suggested by the authors, which includes the 75^th^ percentile cut off of gene scores, the nominal GSEA P-value < 0.01 and the false discovery rate (FDR) < 0.3.

### Functional annotation enrichment analysis

The enrichment of heritability of genomic regions to different functional categories, including cell type-specific elements, was evaluated using the method of LD score regression (https://github.com/bulik/ldsc).[42, 82] This method partitioned the heritability from the discovery GWAS summary statistics of four BP traits (SBP, DBP, PP, and HTN) while accounting for LD among markers.[42] We calculated enrichment, in functional regions and in expanded regions (+500bp) around each functional class, based on functional annotation, using a “full baseline model” previously created from 24 publicly available main annotations that are not specific to any cell type.[42] Enrichment was calculated based on the ratio of explained heritability and the proportion of SNPs in each annotation category. The standard error of enrichment was estimated with a block jackknife to calculate z scores and P values.[42] The multiple testing threshold was determined using the Bonferroni correction while accounting for two independent-trait analyses based on Ji and Li’s method[78] (P of 0.05/[25 classes × 2 traits]). We also performed cell-type-specific group enrichment analysis using cell-type-specific annotations from four histone marks (H3K4me1, H3K4me3, H3K9ac, and H3K27ac), which corresponded to 220 cell types. We divided the 220 cell-type-specific annotations into 10 groups: adrenal/pancreas, central nervous system (CNS), cardiovascular, connective/bone, gastrointestinal, immune/hematopoietic, kidney, liver, skeletal muscle and other. The analysis characterized cell-type-specific annotations within each group and calculated the enrichment of heritability for each group.[42]

### Genomic annotation enrichment

We selected sets of variants in LD r^2^ > 0.1 from the eleven replicated variants, and calculated Bayes Factors and posterior causal probabilities for each variant from the effect sizes and standard errors, as previously described.[83] Each distinct variant associated with multiple traits was included in the analysis only once. The genomic annotations of DHS sites for 348 cell types from the ENCODE project were obtained and grouped into cell types associated with 34 tissues (http://genome.ucsc.edu/ENCODE/cellTypes.html). Four gene-based annotations—coding exon, 5-UTR, 3-UTR, and 1kb upstream of transcription start site (TSS)—from GENCODE transcripts were also obtained. Variants overlapping each of these annotations were then identified. Using the variant annotations and fGWAS (https://github.com/joepickrell/fgwas), we tested for enrichment of variants across all signals in 38 DHS categories, including in the four gene-based annotations in each model.[43]

### Expression quantitative trait loci (eQTL) analysis

We used the GTEx pilot database [82] (http://www.gtexportal.org/home/) to identify eQTLs in the successfully replicated SNPs.

### Integrated haplotype score (iHS) analysis

To evaluate population differentiation and natural selection, using Haplotter,[40] we calculated the integrated haplotype score (iHS) in five cohorts of CARe so that we could measure the amount of extended haplotype homozygosity (http://coruscant.itmat.upenn.edu/whamm/ihs.html). Hence, we tested the evidence of recent positive selection at five significant SNPs with differences in allele frequency across continental-ancestry populations. The measures were standardized (mean 0, variance 1) empirically to the distribution of observed iHS scores over a range of SNPs with similar derived allele frequencies. This method assesses the evidence for selection by comparing the extended homozygosity for haplotypes on a high frequency derived allele relative to the ancestry background.[40]

### Experimental mouse models

Experiments were carried out in accordance with local and the National Institutes of Health guidelines. The animal protocol was approved by the University of Virginia Institutional Animal Care and Use Committee. Wild-type male mice on the 129S6 background at ~ 3 months of age were used for gene expression analyses. All mice were maintained on a 12-hour light-dark cycle with free access to standard chow and water in the animal facility of the University of Virginia.

The hypertension experimental model was induced using Ang II (Sigma-Aldrich, St. Luis, MO) delivered at 600 ng/kg/min for 2 weeks via Alzet mini-osmotic pumps (Durect Corporation, Cupertino, CA, model 2004), as previously described.[84] For gene expression analyses, RNA from kidney tissue was isolated by RNeasy Mini kit (Qiagen) and transcribed to cDNA by iScript ^TM^ cDNA synthesis kit (Bio-Rad). Real time PCR analyses were performed on iQ^TM^5 Multicolor real time PCR Bio-Rad instruments using iQ^TM^ SYBER^®^ Green Supermix. *Hprt* was used as a reference gene for normalization. Sequences of forward and reversed primers (FP and RP) for the gene expression studies are shown in S10 Table.

#### Ethic statement

All research involving human participants have been approved by the Institutional Review Board (IRB) # 04-95-72 and study-related Publication and Presentation committees. All participants have provided informed consent for DNA research and data are publicly available in dbGap.

Animal experiments were carried out following the guidelines established locally at the University of Virginia (UVA) and by the National Institutes of Health. The animal protocol was approved by the UVA Institutional Animal Care and Use Committee (Protocol # 3791, Protocol Title: Genes regulating Hypertension and Kidney Disease). Wild-type male mice on the 129S6 background at ~ 3 months of age were used for gene expression analyses. All mice were maintained on a 12-hour light-dark cycle with free access to standard chow and water in the animal facility UVA.

## Supporting information

S1 Fig Quantile-quantile plots for both individual traits and CPASSOC analysis in discovery stage. (PDF)

S2 Fig Manhattan plots of single trait and CPASSOC analyses at the discovery stage. (PDF)

S3 Fig Regional interrogation of the *HOXA/EVX1*, *ULK4* and *PLEKHG1*. (PDF)

S4 Fig Discovery stage results and linkage disequilibrium maps of the candidate regions. (PDF)

S5 Fig Enrichment for functional annotations of variants in 11 replicated loci reaching genome-wide significance. (PDF)

S1 Table Descriptive characteristics of the discovery studies. (PDF)

S2 Table Genotyping, pre-imputation quality control, imputation and analysis methods in the participating studies. (PDF)

S3 Table Genomic inflation factors by study and analysis. (PDF)

S4 Table Conditional analysis of SNPs with P < 1.0 × 10^−6^ in discovery stage for SBP, DBP, PP, HTN or CPASSOC analysis. (PDF)

S5 Table 72 Independent SNPs with P < 1.0 × 10^−6^ in discovery stage for SBP, DBP, PP, HTN or CPASSOC analysis. (PDF)

S6 Table Trans-ethnic replication of 72 independent SNPs with P < 1.0 × 10^−6^ in discovery stage for SBP, DBP, PP, HTN or CPASSOC. (PDF)

S7 Table Summary of iHS signals in significant loci with frequency differences across ancestry populations. (PDF)

S8 Table MAGENTA analysis. (PDF)

S9 Table eQTL analysis of significant SNPs in tissues. (PDF)

S10 Table Primes for mouse expression experiments. (PDF)

S1 Note Single-trait and multi-trait genome wide association analyses identify novel loci for blood pressure in African-ancestry populations. (DOCX)
