# Correction: Single-trait and multi-trait genome-wide association analyses identify novel loci for blood pressure in African-ancestry populations

**DOI:** 10.1371/journal.pgen.1007345

**Published:** 2018-05-11

**Authors:** Jingjing Liang, Thu H. Le, Digna R. Velez Edwards, Bamidele O. Tayo, Kyle J. Gaulton, Jennifer A. Smith, Yingchang Lu, Richard A. Jensen, Guanjie Chen, Lisa R. Yanek, Karen Schwander, Salman M. Tajuddin, Tamar Sofer, Wonji Kim, James Kayima, Colin A. McKenzie, Ervin Fox, Michael A. Nalls, J. Hunter Young, Yan V. Sun, Jacqueline M. Lane, Sylvia Cechova, Jie Zhou, Hua Tang, Myriam Fornage, Solomon K. Musani, Heming Wang, Juyoung Lee, Adebowale Adeyemo, Albert W. Dreisbach, Terrence Forrester, Pei-Lun Chu, Anne Cappola, Michele K. Evans, Alanna C. Morrison, Lisa W. Martin, Kerri L. Wiggins, Qin Hui, Wei Zhao, Rebecca D. Jackson, Erin B. Ware, Jessica D. Faul, Alex P. Reiner, Michael Bray, Joshua C. Denny, Thomas H. Mosley, Walter Palmas, Xiuqing Guo, George J. Papanicolaou, Alan D. Penman, Joseph F. Polak, Kenneth Rice, Ken D. Taylor, Eric Boerwinkle, Erwin P. Bottinger, Kiang Liu, Neil Risch, Steven C. Hunt, Charles Kooperberg, Alan B. Zonderman, Cathy C. Laurie, Diane M. Becker, Jianwen Cai, Ruth J. F. Loos, Bruce M. Psaty, David R. Weir, Sharon L. R. Kardia, Donna K. Arnett, Sungho Won, Todd L. Edwards, Susan Redline, Richard S. Cooper, D. C. Rao, Jerome I. Rotter, Charles Rotimi, Daniel Levy, Aravinda Chakravarti, Xiaofeng Zhu, Nora Franceschini

The following information is missing from the Funding section: The work was supported by the National Institutes of Health, the National Heart, Lung and Blood Institute 6R21HL121429-03 to TLE and DRVE.
