# Supplementary material for: Single-trait and multi-trait genome-wide association analyses identify novel loci for blood pressure in African-ancestry populations
Source: PLoS Genet. 2017 May 12;13(5):e1006728. doi: 10.1371/journal.pgen.1006728 (PMC5446189; doi:10.1371/journal.pgen.1006728)

S1 Fig. Quantile-quantile plots for both individual traits and CASSOC analysis in discovery stage. **A.** SBP. **B.** DBP. **C.** PP **D.** HTN **F.** S_{Hom} in CPASSOC **G.** S_{Hom} in CPASSOC

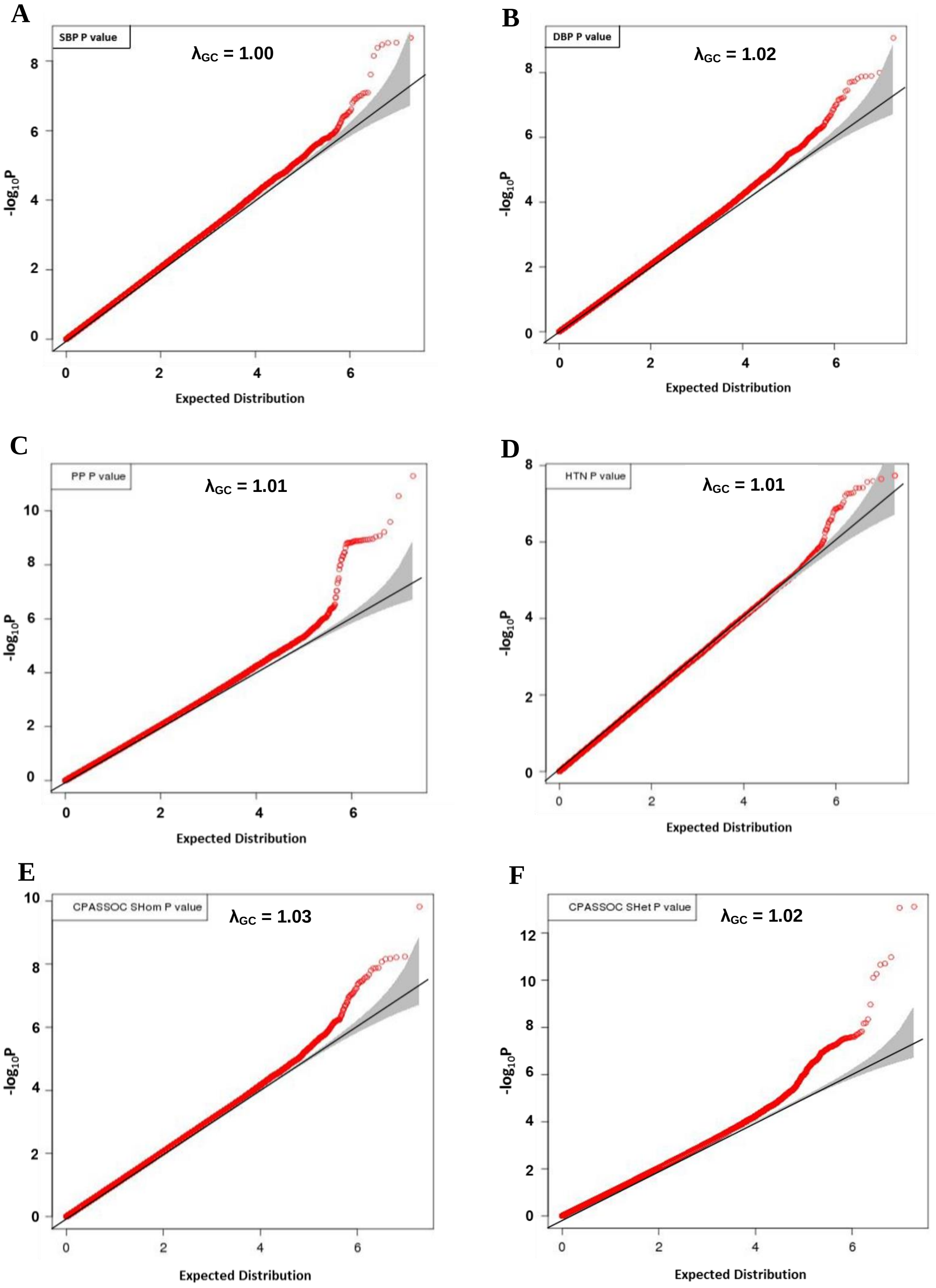

Supplement: S1 Fig — (PDF) [file pgen.1006728.s001.pdf]
