# Supplementary material for: Single-trait and multi-trait genome-wide association analyses identify novel loci for blood pressure in African-ancestry populations
Source: PLoS Genet. 2017 May 12;13(5):e1006728. doi: 10.1371/journal.pgen.1006728 (PMC5446189; doi:10.1371/journal.pgen.1006728)
Supplement: S2 Fig — (PDF) [file pgen.1006728.s002.pdf]

S2 Fig. Manhattan plots of single trait and CPASSOC analyses at the discovery stage. **A.** SBP. **B.** DBP. **C.** PP **D.** HTN **E.** S_{Hom} in CPASSOC **F.** S_{Het} in CPASSOC. The red line is genome-wide significance cutoff of $P = 5.0\times10^{-8}$. Loci that replicated in the combined discovery and replication trans-ethnic meta-analyses at the experimental-wide significance threshold ($P < 1.25\times10^{-8}$) are highlighted using asterix.

A

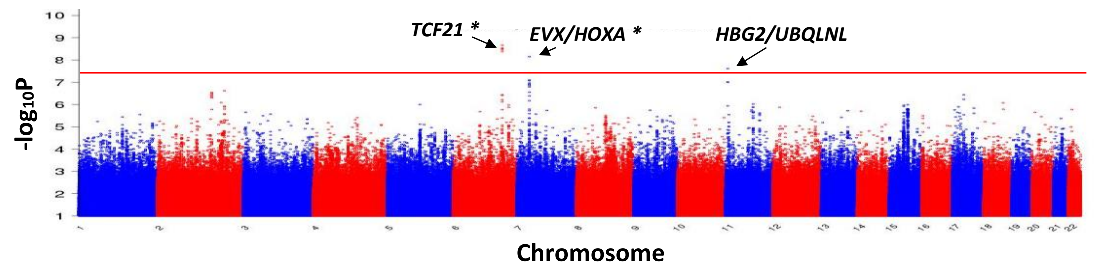

B

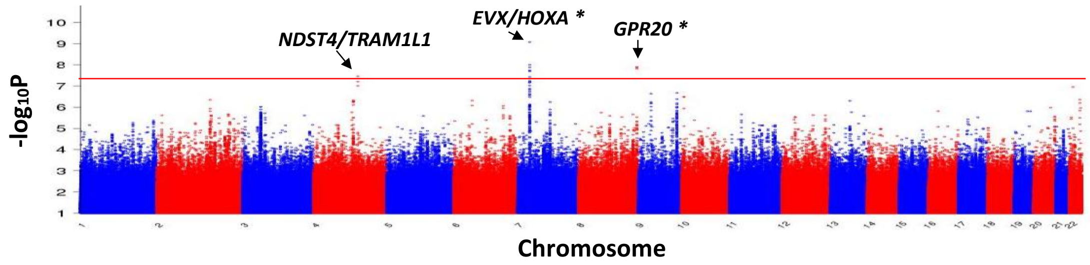

C

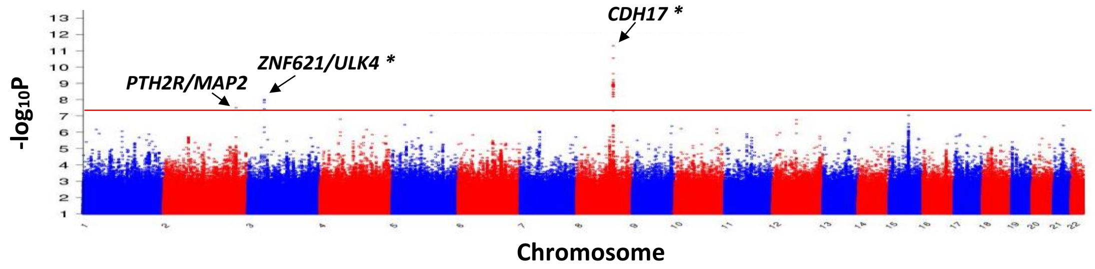

D

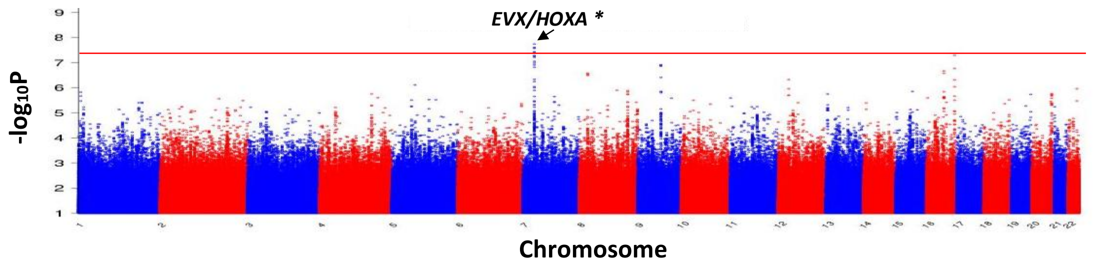

E

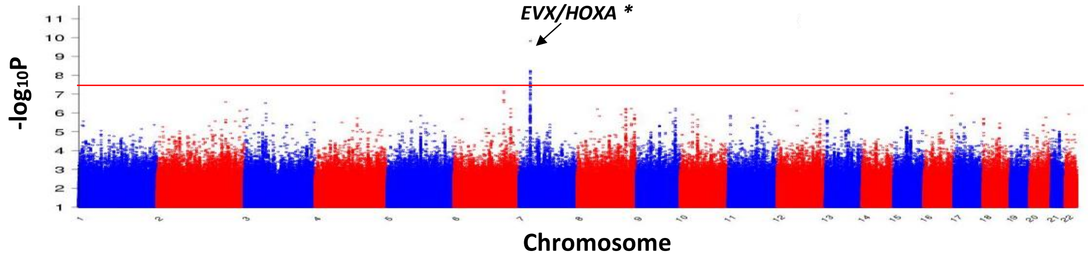
EVX/HOXA *
-log10P
Chromosome

F

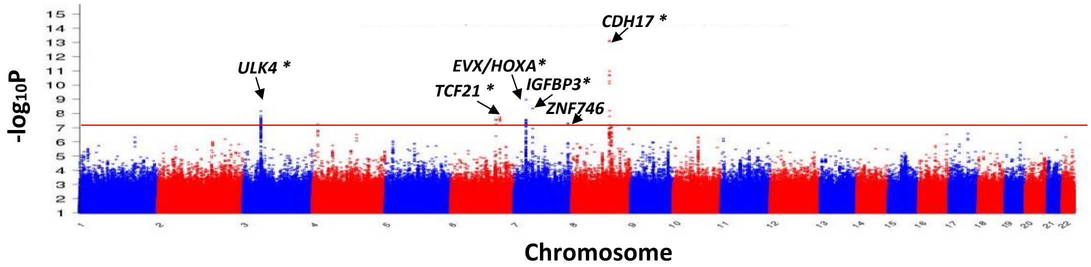
CDH17 *
ULK4 *
EVX/HOXA*
IGFBP3*
TCF21 *
ZNF746
-log10P
Chromosome
