# Supplementary material for: Single-trait and multi-trait genome-wide association analyses identify novel loci for blood pressure in African-ancestry populations
Source: PLoS Genet. 2017 May 12;13(5):e1006728. doi: 10.1371/journal.pgen.1006728 (PMC5446189; doi:10.1371/journal.pgen.1006728)
Supplement: S3 Fig — (PDF) [file pgen.1006728.s003.pdf]

S3 Fig. Regional Interrogation of the **A.** *HOXA/EVX1* locus for SBP **B.** *HOXA/EVX1* locus for DBP **C.** *HOXA/EVX1* locus for S_{Het} of CPASSOC **D.** *GPR20* for DBP **E.** *GPR20* for S_{Het} of CPASSOC **F.** *ULK4* for DBP **G.** *ULK4* for S_{Het} of CPASSOC **H.** PLEKHG1 for SBP.
The *y* axis shows the $-\log_{10}$ P values of SNPs, and the *x* axis shows their chromosomal positions. The lowest P value SNP is plotted as a purple diamond and its correlation with other SNPs in the region is shown in color. The orange triangle is P value in the combined discovery and replication trans-ethnic meta-analysis of the lowest P value SNP.

A

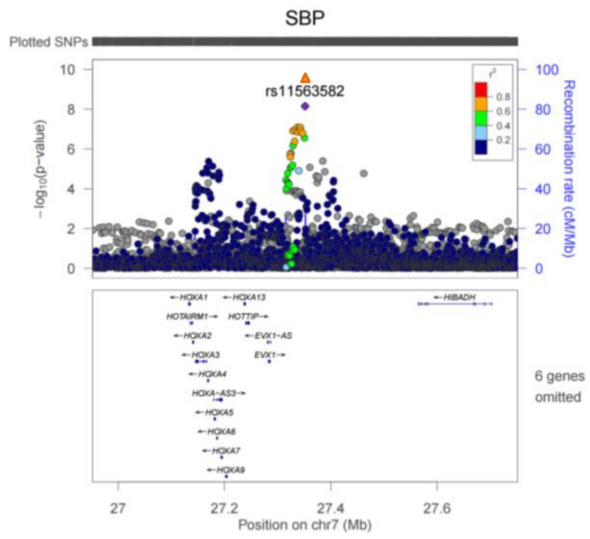

B

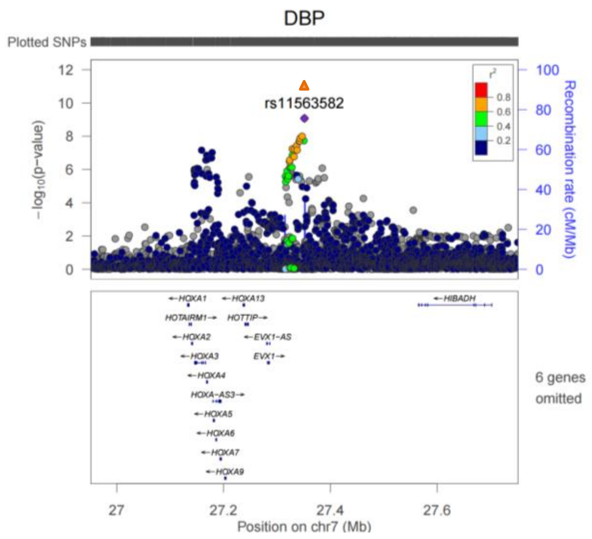

C

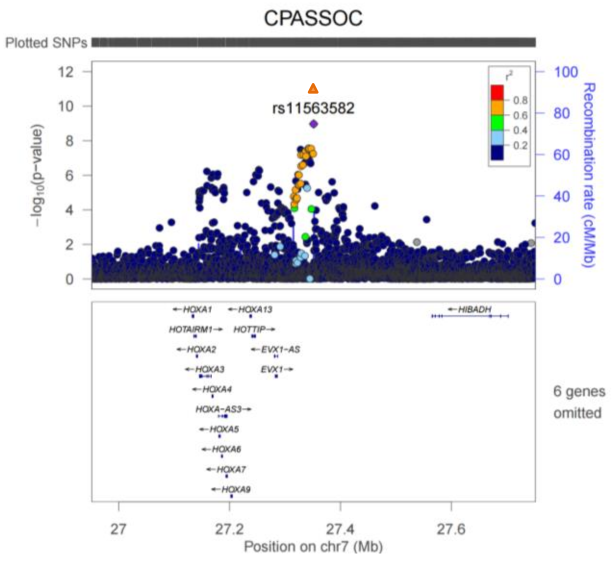

D

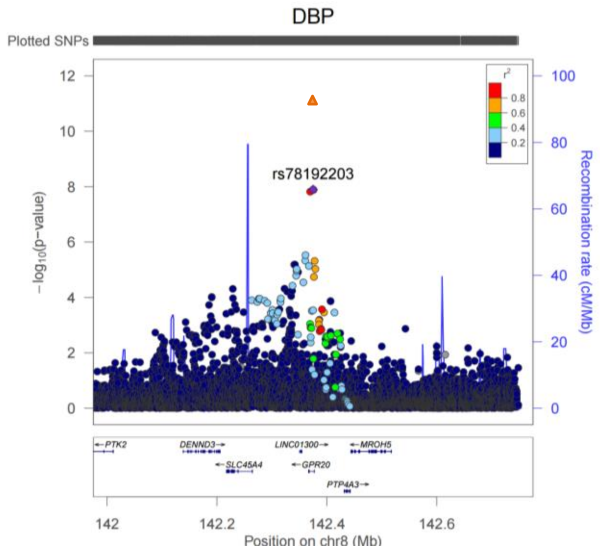

E

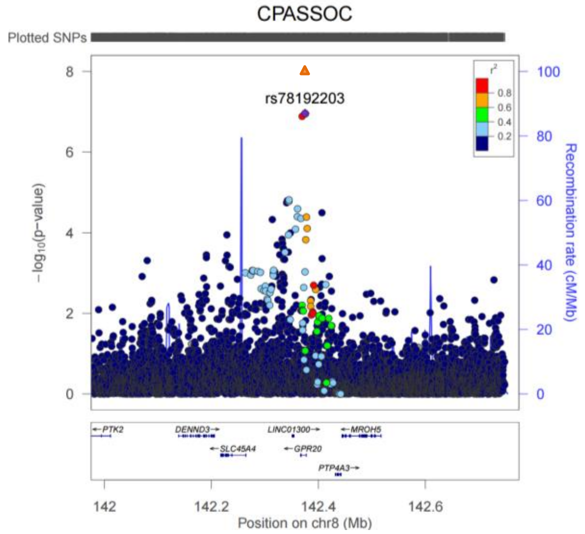
CPASSOC
Plotted SNPs
rs78192203
−log10(p−value)
Recombination rate (cM/Mb)
r²
PTK2
DENND3
SLC45A4
LINC01300
GPR20
MROH5
PTP4A3
Position on chr8 (Mb)

F

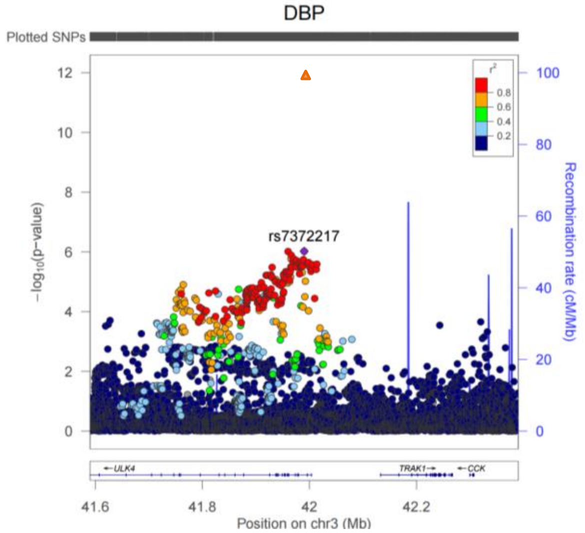
DBP
Plotted SNPs
rs7372217
−log10(p−value)
Recombination rate (cM/Mb)
r²
ULK4
TRAK1
CCK
Position on chr3 (Mb)

G

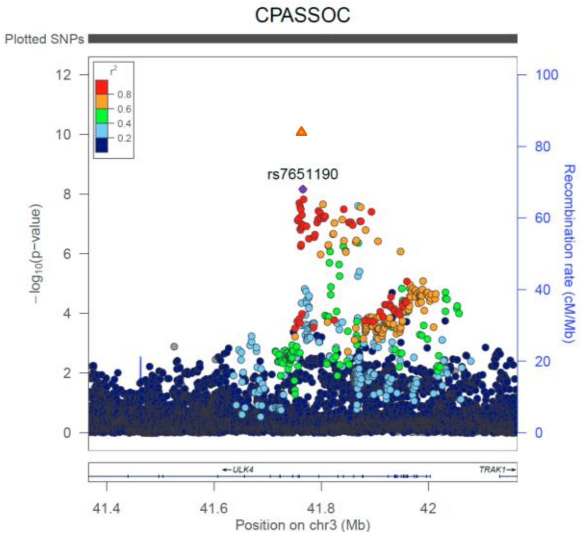
CPASSOC
Plotted SNPs
rs7651190
−log10(p−value)
Recombination rate (cM/Mb)
r²
ULK4
TRAK1
Position on chr3 (Mb)

H

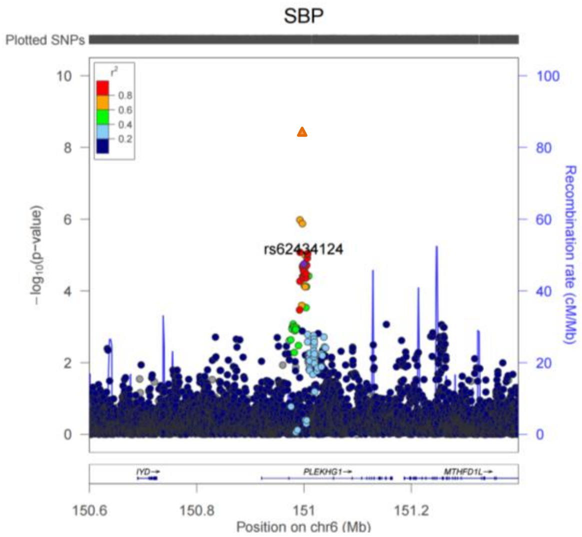
SBP
Plotted SNPs
rs62434124
−log10(p−value)
Recombination rate (cM/Mb)
r²
IYD
PLEKHG1
MTHFD1L
Position on chr6 (Mb)
