# Supplementary material for: Single-trait and multi-trait genome-wide association analyses identify novel loci for blood pressure in African-ancestry populations
Source: PLoS Genet. 2017 May 12;13(5):e1006728. doi: 10.1371/journal.pgen.1006728 (PMC5446189; doi:10.1371/journal.pgen.1006728)
Supplement: S4 Fig — (PDF) [file pgen.1006728.s004.pdf]

S4 Fig. Discovery stage results and linkage disequilibrium maps of the candidate regions. P value plots, genomic structures and LD maps of chromosomes 6q23 (A), p15 (B), 8q24 (C), 8q22 (D), 3p22 (E), 6q26 (F), 9q21 (G), 7p21 (H), 12q14 (I). The red plots represent the $-\log_{10}$ of the P values for association results. The LD maps based on D' (above) and r^2 (below) were drawn using the genotype data of 7717 CARe cohorts. Black triangels indicate the LD blocks identified by Haploview using Gabriel's method. Red dot lines indicate positions of marker SNPs.

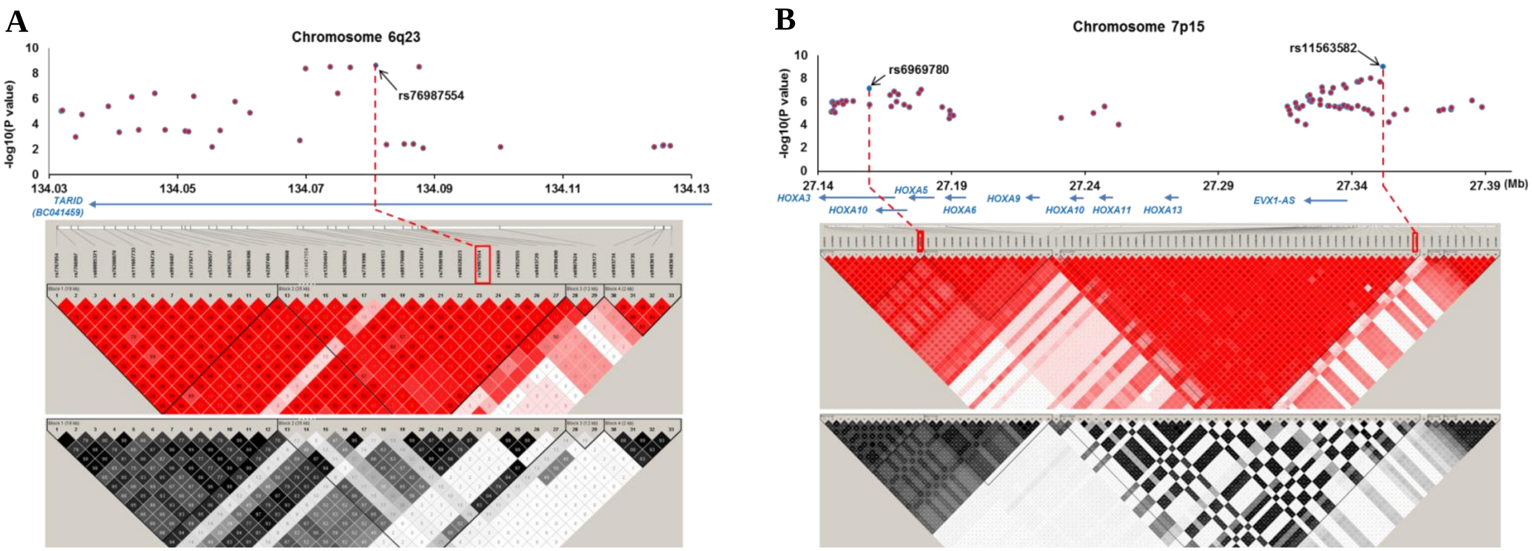

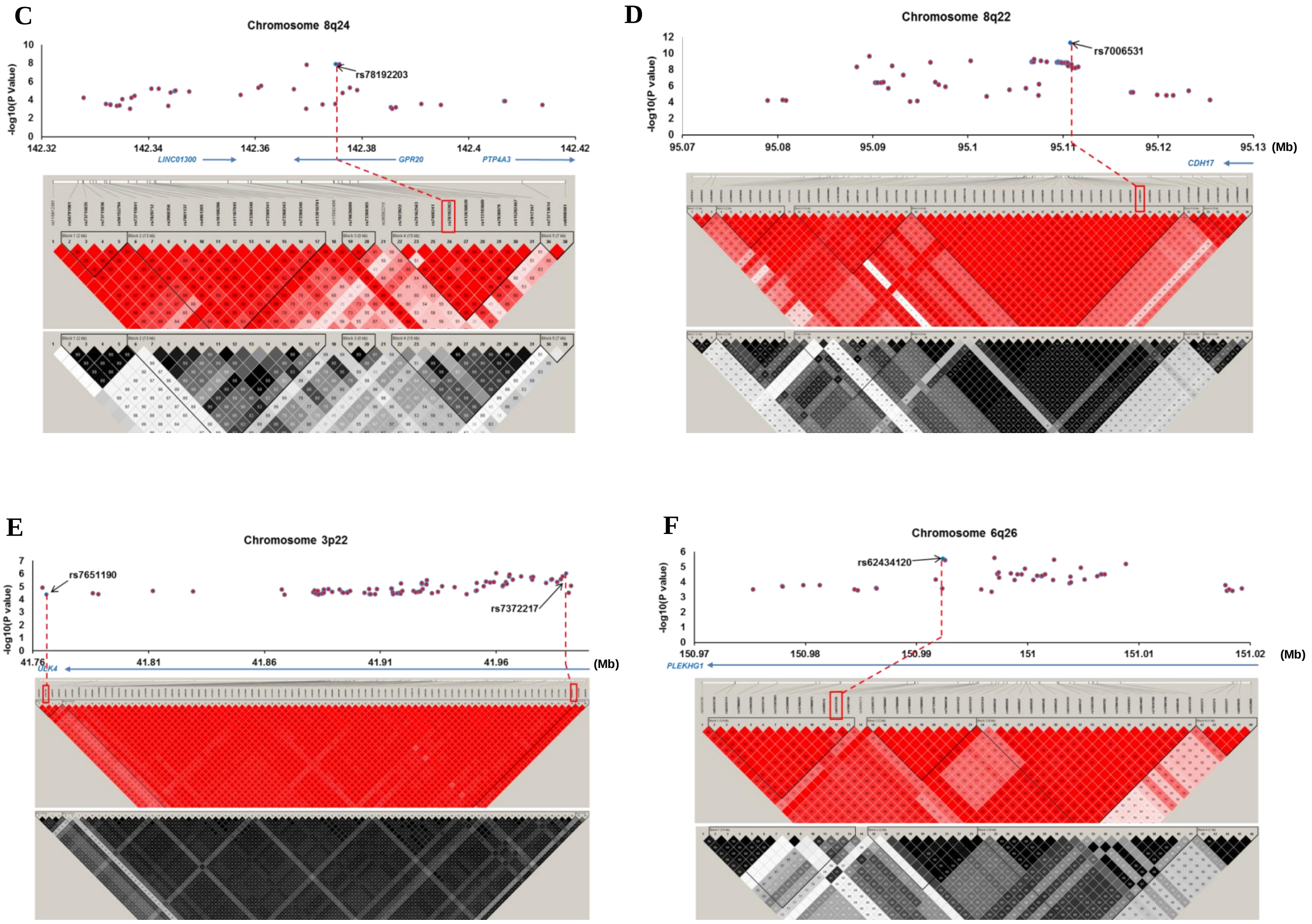
C
Chromosome 8q24
-log10(P Value)
rs78192203
142.32
142.34
142.36
142.38
142.4
142.42
LINC01300
GPR20
PTP4A3
D
Chromosome 8q22
-log10(P value)
rs7006531
95.07
95.08
95.09
95.1
95.11
95.12
95.13
(Mb)
CDH17
E
Chromosome 3p22
-log10(P value)
rs7651190
rs7372217
41.76
41.81
41.86
41.91
41.96
(Mb)
ULK4
F
Chromosome 6q26
-log10(P value)
rs62434120
150.97
150.98
150.99
151
151.01
151.02
(Mb)
PLEKHG1

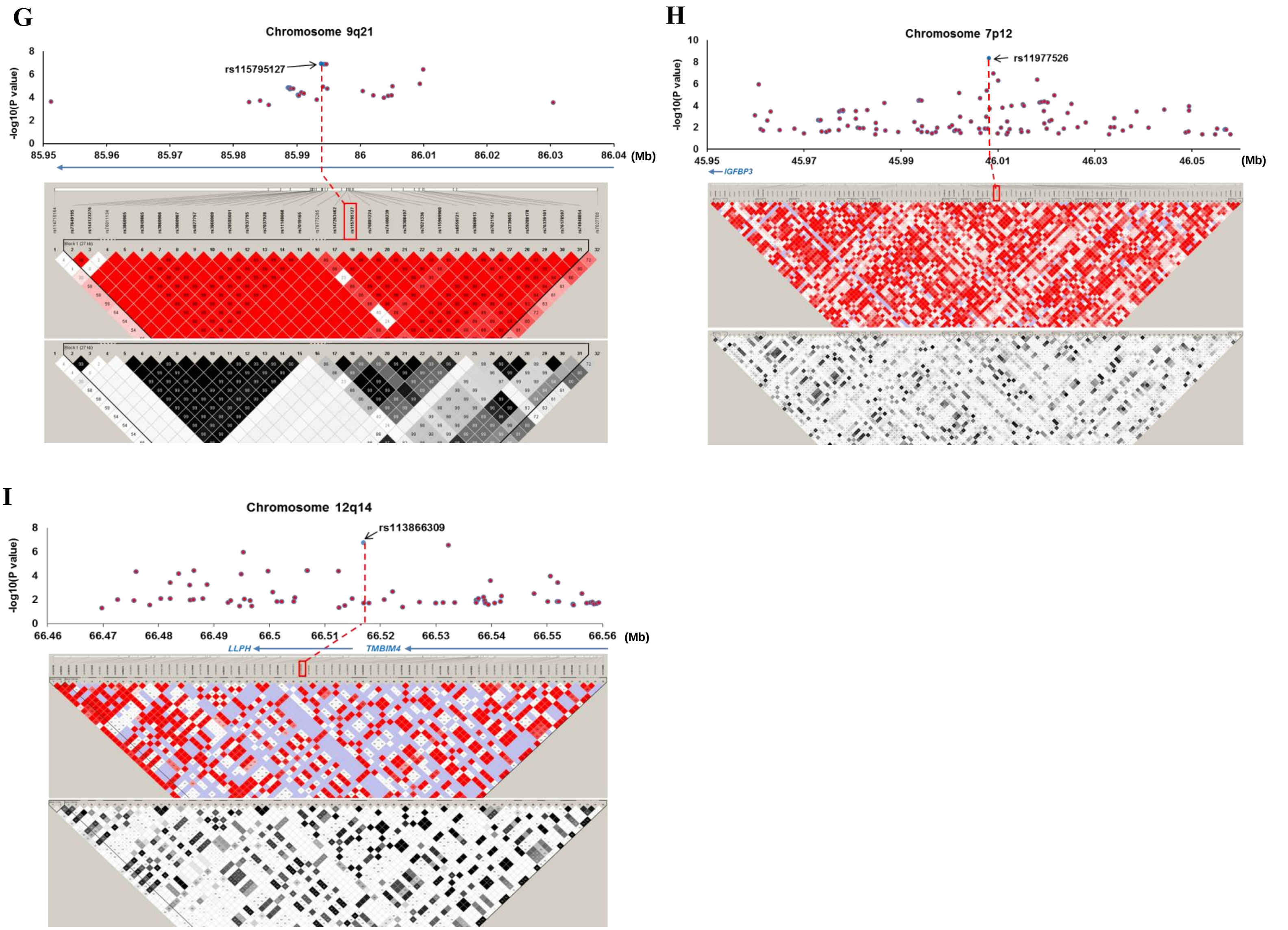
G
Chromosome 9q21
-log10(P value)
rs115795127
0
2
4
6
8
85.95
85.96
85.97
85.98
85.99
86
86.01
86.02
86.03
86.04
(Mb)
H
Chromosome 7p12
-log10(P value)
rs11977526
0
2
4
6
8
10
45.95
45.97
45.99
46.01
46.03
46.05
(Mb)
IGFBP3
I
Chromosome 12q14
-log10(P value)
rs113866309
0
2
4
6
8
66.46
66.47
66.48
66.49
66.5
66.51
66.52
66.53
66.54
66.55
66.56
(Mb)
LLPH
TMBIM4
