# Supplementary material for: Single-trait and multi-trait genome-wide association analyses identify novel loci for blood pressure in African-ancestry populations
Source: PLoS Genet. 2017 May 12;13(5):e1006728. doi: 10.1371/journal.pgen.1006728 (PMC5446189; doi:10.1371/journal.pgen.1006728)

S5 Fig. A. Enrichment of DNase hypersensitive (DHS) sites in 34 tissue categories for variants in $r^2 > 0.1$ with 11 replicated SNPs reaching genome-wide significance using FGWAS **B**. Causal probabilities of variants at the *LLPH/TMBIM4* locus. The most probably causal variant rs12426813 overlaps a DHS site active in blood and cardiovascular cells

A

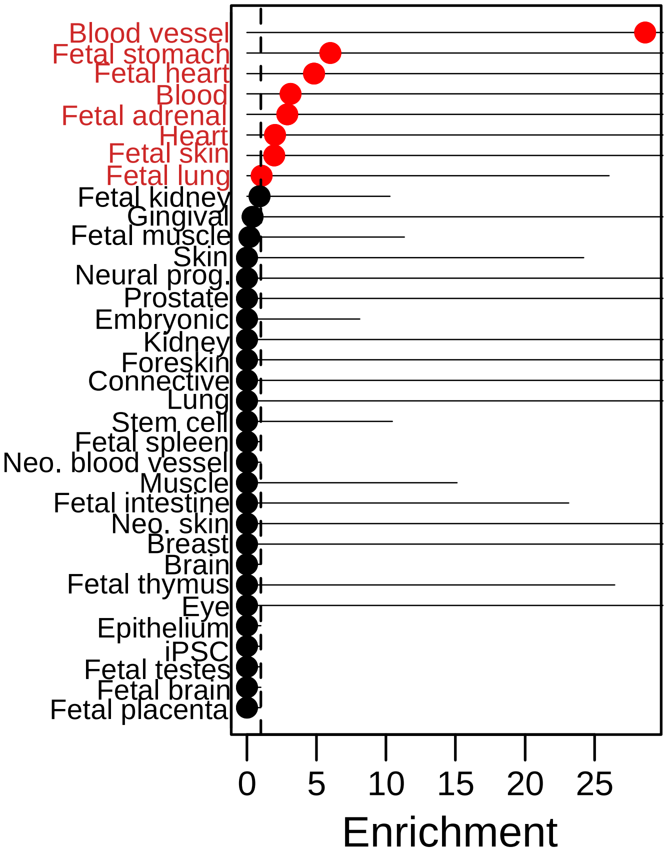

B

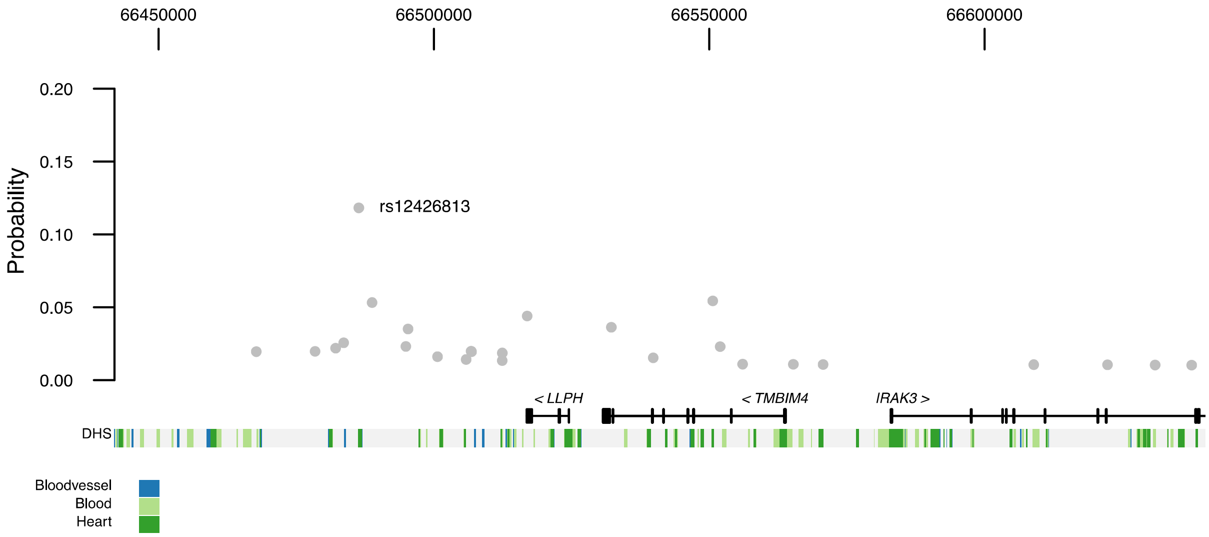

Supplement: S5 Fig — (PDF) [file pgen.1006728.s005.pdf]
