# Supplementary material for: Single-trait and multi-trait genome-wide association analyses identify novel loci for blood pressure in African-ancestry populations
Source: PLoS Genet. 2017 May 12;13(5):e1006728. doi: 10.1371/journal.pgen.1006728 (PMC5446189; doi:10.1371/journal.pgen.1006728)
Supplement: S1 Table — (PDF) [file pgen.1006728.s006.pdf]

S1 Table. Descriptive Characteristics of the Discovery Studies

Study	N	Age (SD)	No. of Males (%)	BMI (SD)	No. with HTN (%)	No. with Hypertensive Medication (%)	SBP, mmHg (SD)	DBP, mmHg (SD)	PP in mmHg
**Discovery Cohorts**									
ARIC_AA	2502	53 (6)	926 (37)	30 (6)	1451 (58)	1076 (43)	135 (23)	84 (13)	51 (16)
CARDIA	826	39 (4)	339 (41)	31 (7)	206 (25)	107 (13)	118 (18)	78 (13)	41 (11)
CFS	608	39 (20)	258 (42)	32 (10)	182 (13)	109 (18)	126 (20)	76 (13)	50 (13)
JHS	2135	55 (13)	807 (38)	32 (7)	1328 (62)	1153 (54)	134 (21)	84 (12)	51 (17)
MESA	1646	62 (10)	746 (45)	30 (6)	1022 (62)	839 (51)	139 (25)	80 (12)	60 (18)
CHS	2064	76 (5)	834 (40)	26 (4)	1132 (55)	979 (47)	133 (20)	69 (11)	63 (18)
GeneSTAR	1129	48 (12)	428 (38)	32 (8)	616 (55)	462 (41)	129 (19)	81 (11)	48 (14)
GENOA	996	56 (11)	295 (30)	31 (7)	688 (69)	550 (56.9)	136 (22)	78 (12)	57 (12)
HANDLS	950	49 (9)	424 (45)	30 (8)	519 (53)	348 (36)	128 (21)	77 (13)	51 (14)
HyperGEN	1256	45 (13)	408 (33)	33 (8)	769 (61)	--	136 (25)	79 (13)	57 (17)
Maywood-Loyola	743	42 (8)	465 (63)	27 (8)	158 (21)	6 (1)	121 (20)	77 (13)	44 (13)
Nigeria-Loyola	1614	49 (15)	674 (42)	24 (5)	797 (49)	399 (25)	135 (30)	84 (19)	52 (16)
Loyola	967	53 (14)	737 (76)	28 (7)	660 (68)	155 (16)	149 (30)	92 (18)	57 (17)
WHI-SHARe	7989	61 (7)	0	32 (7)	4435 (56)	3692 (46)	132 (18)	78 (9)	54 (15)
HUFS	1192	46 (14)	477 (40)	31 (9)	688 (58)	442 (37)	132 (22)	82 (14)	50 (15)
BioMe Biobank	2464	49 (14)	870 (35)	30 (8)	1126 (46)	854 (35)	132 (22)	80 (14)	52 (16)
HRS	1337	67 (10)	483 (36)	31 (7)	1073 (80)	849 (64)	144 (24)	86 (13)	58 (16)
FBPP-AXIOM	917	50 (14)	367 (40)	31 (7)	642 (70)	596 (65)	129 (22)	74 (12)	55 (16)
BioVU eMERGE I AA	1048	49 (16)	330 (31)	32 (9)	510 (49)	246 (23)	135 (22)	81 (13)	54 (17)
BioVU eMERGE II AA 1M	427	48 (15)	204 (48)	30 (8)	264 (62)	169 (40)	138 (24)	83 (14)	56 (17)
BioVU Fibroids AA	407	44 (15)	0 (0)	33 (9)	174 (43)	81 (20)	134 (24)	82 (14)	53 (17)
**Replication Cohorts**									
Jamaica_GXE	613	40 (8)	141 (23)	13 (8)	142 (23)	0	118 (14)	71 (10)	47 (11)
Jamaica_SPT	905	47 (14)	351 (39)	27 (6)	285 (31)	123 (14)	122 (23)	71 (15)	52 (16)
Uganda	2668	35 (14)	774 (29)	24 (5)	1948 (73)	0	126 (21)	80 (12)	46 (15)
WHI_GARNET	4279	65 (7)	0	30 (6)	1721 (40)	1311 (31)	131 (18)	76 (9)	55 (15)
WHI_WHIMS	5478	68 (6)	0	28 (6)	1859 (34)	1403 (26)	130 (18)	75 (9)	56 (15)
ARIC_EA	9687	54 (6)	4650 (48)	27 (5)	2626 (27)	--	118 (16)	72 (10)	47 (13)
BioVU eMERGE II EA 1M	3428	54 (17)	1598 (47)	29 (7)	2500 (73)	1041 (30)	129 (20)	75 (19)	53 (16)
BioVU eMERGE II EA 5M	1045	51 (18)	499 (48)	30 (10)	642 (61)	239 (23)	128 (20)	75 (12)	54 (17)
Korea_hexa	3702	53 (8)	1651 (45)	24 (3)	665 (18)	0	122 (14)	78 (10)	45 (9)
Korea_kare	8773	52 (9)	4117 (47)	25 (3)	2284 (26)	0	122 (19)	80 (11)	41 (12)
Korea_nc2	1814	61 (7)	858 (47)	25 (3)	796 (44)	0	134 (18)	84 (11)	50 (13)
HCHS/SOL	12278	46 (14)	5019 (41)	30 (6)	3445 (28)	2070 (17)	125 (20)	75 (12)	50 (13)
