# Supplementary material for: Single-trait and multi-trait genome-wide association analyses identify novel loci for blood pressure in African-ancestry populations
Source: PLoS Genet. 2017 May 12;13(5):e1006728. doi: 10.1371/journal.pgen.1006728 (PMC5446189; doi:10.1371/journal.pgen.1006728)
Supplement: S2 Table — (PDF) [file pgen.1006728.s007.pdf]

S2 Table: Genotyping, pre-imputation quality control, imputation and analysis methods in the participating studies

	Genotyping Platform	Genotyping algorithm	Pre-imputation exclusive criteria				Variants included for imputation	Impuation Software	Imputation Reference	Variants after imputation	Covariates
			Individual	Call rate	MAF	HWE P-value					
**Discovery Cohorts**											
ARIC	Affymetrix	BirdSeed	> 90%	< 90%	< 1%	< 1.00E-6	796,384	MACH-Admix	1000 Genome Phase I integrated variant set (March 2012)	30,072,738	Age + age*age + sex + bmi + 10PC
CARDIA	Affymetrix	BirdSeed	> 90%	< 90%	< 1%	< 1.00E-6	839,912	MACH-Admix	1000 Genome Phase I integrated variant set (March 2012)	30,072,738	Age + age*age + sex + bmi + 10PC
CFS	Affymetrix	BirdSeed	> 90%	< 90%	< 1%	< 1.00E-6	867,495	MACH-Admix	1000 Genome Phase I integrated variant set (March 2012)	30,072,738	Age + age*age + sex + bmi + 10PC
JHS	Affymetrix	BirdSeed	> 90%	< 90%	< 1%	< 1.00E-6	868,969	MACH-Admix	1000 Genome Phase I integrated variant set (March 2012)	30,072,738	Age + age*age + sex + bmi + 10PC
MESA	Affymetrix	BirdSeed	> 90%	< 90%	< 1%	< 1.00E-6	881,666	MACH-Admix	1000 Genome Phase I integrated variant set (March 2012)	30,072,738	Age + age*age + sex + bmi + 10PC
CHS	Illumina	BeadStudio	>95%	< 97%	none	< 1.00E-5	306,651	MACH	1000 Genome Phase I integrated variant set (March 2012)	38,050,714	Age + age*age + sex + cohort + 10PC
GeneSTAR	Illumina	GenomeStudio	> 90%	< 90%	NA	< 1.00E-6	818,154	IMPUTE 2	1000 Genome Phase I integrated variant set (March 2012)	37,824,549	Age + age*age + sex + BMI + 4PCs
GENOA	Affymetrix+Illumina	BirdSeed and Beads	> 95%	< 95%	N/A	N/A	761,689 (Affy), 1,013,737 (Illumina)	IMPUTE 2	1000 Genome Phase I integrated variant set (March 2012)	30,072,738	Age + age*age + sex + bmi + 10PC
HANDLS	Illumina	Illumina GenomeStudio	> 95%	< 95%	< 1%	< 1.00E-7	907,763	MACH/minimac	1000 Genome Phase I integrated variant set (March 2012 release)	~38M	age + age*age + sex + bmi + 10PCs
HyperGEN	Affymetrix	BirdSeed	> 90%	< 90%	< 1%	< 1.00E-6	408,125	MACH/minimac	1000 Genome Phase I integrated variant set (March 2012)	37,824,549	Age + age*age + sex + bmi + 10PC
Maywood-Loyola	Affymetrix	BirdSeed	> 90%	< 90%	< 1%	< 1.00E-6	965,441	MACH-Admix	1000 Genome Phase I integrated variant set (March 2012)	30,072,738	Age + age*age + sex + BMI + PC1-PC3
Nigeria-Loyola	Affymetrix	BirdSeed	> 90%	< 90%	< 1%	< 1.00E-6	556,307	MACH-Admix	1000 Genome Phase I integrated variant set (March 2012)	30,072,738	Age + age*age + sex + BMI +PC1
Loyola	Axiom GW Human Array	Axiom GT1	> 90%	< 90%	< 1%	< 1.00E-6	607,982	MACH-Admix	1000 Genome Phase I integrated variant set (March 2012)	30,072,738	Age + age*age + sex + bmi + 10PC
WHI-SHARe	Affymetrix 6.0	Birdseed v2	>95%	<95%	<1%	<1.00E-6	728,334	MACH/minimac	1000 Genome Phase I integrated variant set (March 2012)	30,072,738	Age + age*age + bmi + center + 10PC
HUFS	Affymetrix	Birdseed	> 90%	< 90%	< 1%	< 1.00E-6	500,237	MACH-Admix	1000 Genome Phase I integrated variant set (March 2012)	30,072,738	Age + age*age + sex + bmi + 3PC
BioME Biobank	Illumina	GenomeStudio	> 90%	< 95%	< 1%	< 1.00E-5	845,969	IMPUTE2	1000 Genome Phase I integrated variant set (March 2012)	-	Age + Age*Age + Sex + BMI + PC1-PC2
HRS	Illumina	GenomeStudio	> 98%	< 98%	< 1%	< 1.00E-4	2,195,306	IMPUTE2	1000 Genome Phase I integrated variant set (March 2012)	30,072,738	Age + age*age + sex + bmi + 10PC
FBPP	Affymetrix	Birdseed	> 90%	< 90%	< 1%	< 1.00E-6	777,125	MACH-Admix	1000 Genome Phase I integrated variant set (March 2012)	30,072,738	Age + age*age + sex + bmi + 10PC
BioVU eMERGE I AA	Illumina 1M	GenomeStudio	>95%	>95%	< 1%	< 1.00E-6	1,001,273	IMPUTE version 2.3.0	1000 Genome Phase III (October, 2014)	81,682,398	Age + age*age + sex + bmi + 10PC
BioVU eMERGE II AA 1M	Illumina 1M	GenomeStudio	>95%	>95%	< 1%	< 1.00E-6	826,686	IMPUTE version 2.3.0	1000 Genome Phase III (October, 2014)	81,163,150	Age + age*age + sex + bmi + 10PC
BioVU Fibroids AA	Affymetrix Biobank array and World Array 2	BirdSeed	>95%	>95%	< 1%	< 1.00E-6	1,345,099	IMPUTE version 2.3.0	1000 Genome Phase III (October, 2014)	81,064,813	Age + age*age + sex + bmi + 10PC
**Replication Cohorts**											
Jamaica_GXE	Illumina Metabochip	GeneCall	> 90%	< 90%	< 1%	< 1.00E-6	400,931	MACH-Admix	1000 Genome Phase I integrated variant set (March 2012)	30,072,738	Age + age*age + sex + BMI + PC1-PC3
Jamaica_SPT	Illumina Metabochip	GeneCall	> 90%	< 90%	< 1%	< 1.00E-6	387,665	MACH-Admix	1000 Genome Phase I integrated variant set (March 2012)	30,072,738	Age + age*age + sex + BMI + PC1-PC3
Uganda	Gentra Puregene	GeneCall	> 90%	< 90%	< 1%	< 1.00E-6	-	-	-	-	Age + age*age + sex + BMI
WHI_GARNET	Illumina HumanOmni1-Quad v1-0 B	Birdseed v2	>97%	>98%	<1%	<1.00E-4	844,092	Beagle/MACH	1000 Genome Phase I integrated variant set (March 2012)	30,072,738	Age + age*age + bmi + center + 10PC
WHI_WHIMS	HumanOmniExpressExome-8v1_B	Birdseed v2	>98%	>98%	<1%	<1.00E-4	907,365	Beagle/MACH	1000 Genome Phase I integrated variant set (March 2012)	30,072,738	Age + age*age + bmi + center + 10PC
ARIC_white	Affymetrix	BirdSeed	> 90%	< 90%	< 1%	< 1.00E-6	776,305	MACH-Admix	1000 Genome Phase I integrated variant set (March 2012)	30,072,738	
BioVU eMERGE II EA 1M	Illumina 1M	GenomeStudio	>95%	>95%	< 1%	< 1.00E-6	826,686	IMPUTE version 2.3.0	1000 Genome Phase III (October, 2014)	81,422,501	Age + age*age + sex + bmi + 10PC
BioVU eMERGE II EA 5M	Illumina 5M	GenomeStudio	>95%	>95%	< 1%	< 1.00E-6	2,404,308	IMPUTE version 2.3.0	1000 Genome Phase III (October, 2014)	81,011,298	Age + age*age + sex + bmi + 10PC
Korea_hexa	Affymetrix	BirdSeed	> 5%	< 95%	< 5%	< 1.00E-5	4,228	SHAPEIT/IMPUTE2	1000 Genome Phase I integrated variant set (March 2012)	361,521	Age + age*age + sex + bmi + 10PC
Korea_kare	Affymetrix	BirdSeed	> 5%	< 95%	< 5%	< 1.00E-5	3,865	SHAPEIT/IMPUTE2	1000 Genome Phase I integrated variant set (March 2012)	361,476	Age + age*age + sex + bmi + 10PC
Korea_nc2	Affymetrix	BirdSeed	> 5%	< 95%	< 5%	< 1.00E-5	6,569	SHAPEIT/IMPUTE2	1000 Genome Phase I integrated variant set (March 2012)	361,680	Age + age*age + sex + bmi + 10PC
HCHS/SOL	Illumina Omni 2.5M + Custom	GenomeStudio	<98%	<98%	NA	<10E-5	2,294,032	IMPUTE2	1000 Genome phase 3	49,744,721	Age + age*age + sex + study center + bmi + 5PC + log(sampling weights)

PCs indicates principal components; QC indicated quality control
