# Supplementary material for: Single-trait and multi-trait genome-wide association analyses identify novel loci for blood pressure in African-ancestry populations
Source: PLoS Genet. 2017 May 12;13(5):e1006728. doi: 10.1371/journal.pgen.1006728 (PMC5446189; doi:10.1371/journal.pgen.1006728)
Supplement: S3 Table — (PDF) [file pgen.1006728.s008.pdf]

S3 Table: Genomic inflation factors by study and analysis.

**Study**	**N**	**SBP - λ_{GC}**	**DBP - λ_{GC}**	**HTN - λ_{GC}**	**PP - λ_{GC}**
ARIC	2502	1	1.02	1.01	1.01
CARDIA	826	0.99	0.99	1	1.01
CFS	608	1.05	1.03	1.02	1.04
CHS	815	1.01	1	1	1.02
FBPP	917	0.96	0.98	0.97	0.99
GENOA	996	0.99	0.99	1	0.99
JHS	2135	0.98	0.99	0.99	1.01
MESA	1646	0.98	1.01	1	0.99
HyperGen	1256	1.08	1.07	1.05	1.06
Loyola	967	0.99	1	0.98	0.99
HRS	1337	0.99	1.01	0.99	1
HUFS	1192	1.06	1.03	1.03	1.02
IPM	2464	0.98	0.99	1	1
Maywood	743	1.01	1.02	1.01	1
WHI	7989	1.01	1.03	1.03	1.02
HANDLS	950	0.99	1	1	1.02
Nigeria	1614	1.09	1.05	1.02	1.02
GeneSTAR	1129	1.02	1.06	1.04	1
eMERGE	1048	1.03	1.05	1.01	1.02
OMNI	427	1.01	1	0.99	1.01
FIBROID	407	0.99	1.02	1.04	1
Total	31968	1	1.02	1.01	1.01

λ indicates the genomic inflation factor. N indicates sample size.
