# Supplementary material for: Single-trait and multi-trait genome-wide association analyses identify novel loci for blood pressure in African-ancestry populations
Source: PLoS Genet. 2017 May 12;13(5):e1006728. doi: 10.1371/journal.pgen.1006728 (PMC5446189; doi:10.1371/journal.pgen.1006728)
Supplement: S4 Table — (PDF) [file pgen.1006728.s009.pdf]

S4 Table. Conditional analysis of SNPs with P < 1.0E-06 in discover stage for single trait analysis

Conditional SNP (Independent SNP for replication)	SNP	Chr	Pos	A1	A2	Gene	Effect	SE	P.value	Effect (conditional)	SE (conditional)	P.value (conditional)
						**SBP**						
	rs9918487	6	134042960	A	G		-1.2639	0.2555	7.55E-07	-0.2810	0.1957	0.1511
	rs57850577	6	134046495	A	G		1.2660	0.2489	3.64E-07	0.3393	0.1949	0.0817
	rs79889868	6	134052576	C	G		-1.2168	0.2440	6.13E-07	-0.3251	0.1932	0.0925
	rs80176668	6	134069965	A	G	*TARID/*	1.7082	0.2906	4.15E-09	0.1825	0.1395	0.1908
rs76987554	rs112734474	6	134073797	T	G	*TCF21**	1.7256	0.2910	3.02E-09	0.1921	0.1379	0.1638
	rs79590186	6	134075021	T	C		-1.3953	0.2749	3.87E-07	-0.1150	0.1726	0.5054
	rs80328223	6	134076947	T	C		-1.7232	0.2915	3.39E-09	-0.1808	0.1362	0.1844
	rs76987554	6	134080855	T	C		-1.8492	0.3090	2.17E-09	NA	NA	NA
	rs79030490	6	134087689	A	C		-1.8344	0.3091	2.96E-09	NA	NA	NA
	rs17428380	7	27328929	T	C		1.3794	0.2609	1.24E-07	0.0863	0.1347	0.5220
	rs12535894	7	27329173	C	G		-1.2687	0.2553	6.72E-07	-0.0601	0.1469	0.6825
	rs113318709	7	27332148	T	C		1.3424	0.2651	4.12E-07	0.0128	0.1323	0.9231
	rs148340546	7	27333162	A	G		-1.3761	0.2590	1.08E-07	-0.1058	0.1375	0.4418
	rs7777128	7	27337113	C	G		1.3821	0.2577	8.13E-08	0.1173	0.1366	0.3905
	rs17428471	7	27337867	T	G		1.3276	0.2522	1.41E-07	0.1690	0.1534	0.2706
rs11563582	rs17438166	7	27341976	T	C	*EVX1/HOXA*	1.3827	0.2581	8.43E-08	0.1100	0.1351	0.4158
	rs1009547	7	27342727	A	G		1.3847	0.2582	8.15E-08	0.1111	0.1351	0.4110
	rs55831032	7	27343535	T	C		1.3716	0.2593	1.23E-07	0.0878	0.1343	0.5134
	rs17438292	7	27347127	A	G		-1.3848	0.2644	1.62E-07	-0.0577	0.1317	0.6612
	rs17502580	7	27350607	A	G		-1.3376	0.2603	2.77E-07	-0.0581	0.1374	0.6724
	rs11563582	7	27351650	A	G		1.6125	0.2786	7.09E-09	NA	NA	NA
	rs7941648	11	5532222	T	G		-1.2323	2.21E-01	2.43E-08	NA	NA	NA
rs7941648	rs3763880	11	5533606	T	G	*HGB2*	1.4268	0.2679	1.01E-07	0.4701	0.2059	0.0224
	rs72887764	11	5545271	T	C		1.2862	0.2413	9.77E-08	0.4505	0.1893	0.0173
						**DBP**						
	rs7651190	3	41765955	A	G		-0.45	0.1099	4.20E-05	-0.3366	0.1824	0.0010
rs7372217	rs1716975	3	41960006	T	C	*ULK4*	0.5759	0.1176	9.67E-07	NA	NA	NA
	rs7372217	3	41990122	A	G		-0.5514	0.1125	9.50E-07	NA	NA	NA
	rs7676999	4	116987529	A	G		1.3123	2.38E-01	3.50E-08	NA	NA	NA
rs62312401	4:116968554:GTTT	4	116934079	T	C	*NDST4/TRAM1L1*	-1.2314	0.2311	9.86E-08	-0.1433	0.1203	0.2337
	rs62312401	4	116968554	G	GTTTAT		-1.2686	0.2344	6.19E-08	-0.2317	0.1400	0.0979
	rs2428433	7	27145517	T	C		-0.5765	0.1178	9.94E-07	-0.5602	0.1178	1.98E-06
	rs73071550	7	27149099	T	C		-0.5692	0.116	9.17E-07	-0.5541	0.1160	1.78E-06
	rs6461985	7	27150634	T	C		-0.5699	0.1158	8.60E-07	-0.5551	0.1158	1.64E-06
	rs7798733	7	27153281	C	G		-0.5691	0.1159	9.05E-07	-0.5541	0.1159	1.75E-06
	rs6969780	7	27159136	C	G		0.6214	0.1152	6.95E-08	0.5591	0.1148	1.11E-06
	rs6461987	7	27166956	C	G		0.6364	0.1238	2.73E-07	0.5453	0.1229	9.17E-06
	rs1801085	7	27168590	A	G		-0.5972	0.1134	1.40E-07	-0.5351	0.1130	2.17E-06
	rs6962314	7	27170159	T	C		0.6283	0.1218	2.46E-07	0.5416	0.1210	7.63E-06
	rs6976129	7	27177746	T	C		0.6462	0.1243	2.03E-07	0.5558	0.1235	6.73E-06
	rs17471520	7	27178790	T	C		-0.6073	0.1138	9.58E-08	-0.5381	0.1133	2.02E-06
	rs17502232	7	27323604	T	G		-0.8317	0.163	3.35E-07	0.0601	0.0738	0.4154
	rs17473410	7	27324196	T	G		-0.8396	0.1632	2.68E-07	0.0547	0.0735	0.4566
	rs17473424	7	27324369	A	G		0.7873	0.1592	7.65E-07	-0.0475	0.0827	0.5662
	rs73073487	7	27324984	A	G		0.8186	0.1672	9.83E-07	-0.0810	0.0804	0.3141
rs11563582	rs17502260	7	27325313	T	C		-0.7875	0.1592	7.49E-07	0.0474	0.0827	0.5664
	rs6961048	7	27328187	C	G		-0.7732	0.1563	7.49E-07	0.0381	0.0834	0.6473
	rs17428380	7	27328929	T	C		0.8543	0.1575	5.78E-08	0.0088	0.0763	0.9079
	rs12535894	7	27329173	C	G		-0.8122	0.1541	1.36E-07	-0.0222	0.0847	0.7933
	rs113318709	7	27332148	T	C		0.8363	0.1599	1.70E-07	-0.0321	0.0745	0.6667
	rs148340546	7	27333162	A	G		-0.846	0.1563	6.21E-08	-0.0159	0.0783	0.8392
	rs7777128	7	27337113	C	G		0.8556	0.1555	3.74E-08	0.0294	0.0778	0.7057
	rs17428471	7	27337867	T	G		0.8208	0.1521	6.85E-08	0.0648	0.0892	0.4675
	rs17438166	7	27341976	T	C		0.8739	0.1557	2.01E-08	0.0429	0.0769	0.5769
	rs1009547	7	27342727	A	G		0.8756	0.1558	1.90E-08	0.0435	0.0768	0.5705
	rs55831032	7	27343535	T	C		0.8894	0.1565	1.32E-08	0.0502	0.0761	0.5095
	rs17438292	7	27347127	A	G		-0.9136	0.1594	1.00E-08	-0.0481	0.0743	0.5173
	rs17502580	7	27350607	A	G		-0.8833	0.157	1.86E-08	-0.0478	0.0782	0.5412
	rs11563582	7	27351650	A	G		1.0151	0.1654	8.45E-10	NA	NA	NA
	rs917206	7	27385004	A	G	*EVX1/HOXA*	-1.2058	0.2447	8.32E-07	-0.7071	0.2310	0.0022
	rs2428433	7	27145517	T	C		-0.5765	0.1178	9.94E-07	-0.0402	0.0498	0.4194
	rs73071550	7	27149099	T	C		-0.5692	0.116	9.17E-07	-0.0353	0.0459	0.4421
	rs6461985	7	27150634	T	C		-0.5699	0.1158	8.60E-07	-0.0359	0.0453	0.4273
	rs7798733	7	27153281	C	G		-0.5691	0.1159	9.05E-07	-0.0336	0.0449	0.4538
	rs6969780	7	27159136	C	G		0.6214	0.1152	6.95E-08	NA	NA	NA
	rs6461987	7	27166956	C	G		0.6364	0.1238	2.73E-07	0.0514	0.0695	0.4594
	rs1801085	7	27168590	A	G		-0.5972	0.1134	1.40E-07	NA	NA	NA
	rs6962314	7	27170159	T	C		0.6283	0.1218	2.46E-07	0.0476	0.0660	0.4708
	rs6976129	7	27177746	T	C		0.6462	0.1243	2.03E-07	0.0635	0.0709	0.3701
	rs17471520	7	27178790	T	C		-0.6073	0.1138	9.58E-08	-0.0241	0.0411	0.5575
	rs17502232	7	27323604	T	G		-0.8317	0.163	3.35E-07	0.0679	0.0738	0.3575
	rs17473410	7	27324196	T	G		-0.8396	0.1632	2.68E-07	0.0623	0.0734	0.3964
	rs17473424	7	27324369	A	G		0.7873	0.1592	7.65E-07	-0.0552	0.0827	0.5045
	rs73073487	7	27324984	A	G		0.8186	0.1672	9.83E-07	-0.0858	0.0804	0.2862
rs11563582 rs6969780	rs17502260	7	27325313	T	C		-0.7875	0.1592	7.49E-07	0.0551	0.0827	0.5048
	rs6961048	7	27328187	C	G		-0.7732	0.1563	7.49E-07	0.0470	0.0833	0.5730
	rs17428380	7	27328929	T	C		0.8543	0.1575	5.78E-08	-0.0014	0.0763	0.9852
	rs12535894	7	27329173	C	G		-0.8122	0.1541	1.36E-07	-0.0119	0.0847	0.8884
	rs113318709	7	27332148	T	C		0.8363	0.1599	1.70E-07	-0.0405	0.0744	0.5863
	rs148340546	7	27333162	A	G		-0.846	0.1563	6.21E-08	-0.0061	0.0783	0.9375
	rs7777128	7	27337113	C	G		0.8556	0.1555	3.74E-08	0.0189	0.0778	0.8078
	rs17428471	7	27337867	T	G		0.8208	0.1521	6.85E-08	0.0469	0.0892	0.5990
	rs17438166	7	27341976	T	C		0.8739	0.1557	2.01E-08	0.0340	0.0768	0.6585
	rs1009547	7	27342727	A	G		0.8756	0.1558	1.90E-08	0.0347	0.0767	0.6511
	rs55831032	7	27343535	T	C		0.8894	0.1565	1.32E-08	0.0418	0.0761	0.5825
	rs17438292	7	27347127	A	G		-0.9136	0.1594	1.00E-08	-0.0410	0.0743	0.5808
	rs17502580	7	27350607	A	G		-0.8833	0.157	1.86E-08	-0.0447	0.0782	0.5673
	rs11563582	7	27351650	A	G		1.0151	0.1654	8.45E-10	NA	NA	NA
	rs917206	7	27385004	A	G		-1.2058	0.2447	8.32E-07	-0.6353	0.2306	0.0059
	rs2123202	9	28153553	A	C		0.6117	0.1229	6.46E-07	0.1099	0.0755	0.1454
	rs13294724	9	28157548	A	G		0.6036	0.1231	9.38E-07	0.1035	0.0762	0.1745

rs71512425	rs114334738	9	28165671	T	C		1.2762	0.2552	5.73E-07	0.9782	0.2490	0.0001
	rs71512425	9	28165694	A	G		0.6974	0.1348	2.32E-07	NA	NA	NA
	rs115412864	9	28165783	T	C	*LINGO2*	1.2684	0.2551	6.65E-07	0.9704	0.2488	9.63E-05
	rs2123202	9	28153553	A	C		0.6117	0.1229	6.46E-07	0.1080	0.0755	0.1524
	rs13294724	9	28157548	A	G		0.6036	0.1231	9.38E-07	0.1006	0.0762	0.1868
rs71512425 rs114334738	rs114334738	9	28165671	T	C		1.2762	0.2552	5.73E-07	NA	NA	NA
	rs71512425	9	28165694	A	G		0.6974	0.1348	2.32E-07	NA	NA	NA
	rs115412864	9	28165783	T	C		1.2684	0.2551	6.65E-07	NA	NA	NA
						**HTN**						
	rs17501431	7	27251549	T	C		-0.1876	0.0355	1.24E-07	-0.0690	0.0286	0.0157
	rs17472588	7	27252767	A	C		0.2005	0.0369	5.33E-08	0.0670	0.0283	0.0177
	rs17501438	7	27253688	C	G		-0.197	0.0364	6.18E-08	-0.0673	0.0282	0.0169
	rs57330666	7	27274187	A	T		-0.1757	0.0351	5.73E-07	-0.0448	0.0263	0.0880
	rs17473046	7	27287676	A	C		0.1691	0.0344	9.17E-07	0.0381	0.0253	0.1320
	rs17501898	7	27289410	A	G		-0.1682	0.0344	9.93E-07	-0.0376	0.0254	0.1381
	rs28570591	7	27319622	A	G		-0.1768	0.0351	4.66E-07	-0.0015	0.0161	0.9249
	rs10279895	7	27328210	A	G		0.1894	0.0337	1.84E-08	NA	NA	NA
	rs17473487	7	27331139	T	C		-0.1879	0.0337	2.54E-08	NA	NA	NA
rs10279895	rs185372147	7	27331552	T	G	*EVX1/HOXA*	0.1879	0.0337	2.54E-08	NA	NA	NA
	rs142887200	7	27333712	T	C		-0.1868	0.0336	2.68E-08	NA	NA	NA
	rs11564025	7	27335536	T	G		0.1843	0.0335	3.80E-08	NA	NA	NA
	rs11564024	7	27335561	T	G		0.179	0.0341	1.55E-07	NA	NA	NA
	rs10227075	7	27338363	T	C		0.1823	0.0335	5.45E-08	NA	NA	NA
	rs10270510	7	27338373	C	G		-0.1844	0.0335	3.87E-08	NA	NA	NA
	rs11564019	7	27338558	A	G		-0.1844	0.0335	3.87E-08	NA	NA	NA
	rs17473690	7	27342386	T	G		-0.183	0.0337	5.45E-08	NA	NA	NA
	rs11564010	7	27344629	C	G		-0.1802	0.0337	8.93E-08	NA	NA	NA
	rs17502552	7	27346216	T	G		-0.1801	0.0339	1.04E-07	NA	NA	NA
	rs12149202	16	85700360	A	G		-0.1877	0.0345	5.14E-08	NA	NA	NA
rs12149202	rs12149210	16	85700481	A	G	*GSE1*	-0.1608	0.032	4.90E-07	NA	NA	NA
	16:85708762:T_TTA	16	85708762	T	TTAAG		0.1576	0.0321	9.22E-07	0.0352	0.0229	0.1244
	rs3815795	16	85712105	T	C		-0.1659	0.0317	1.72E-07	-0.0213	0.0173	0.2173
						**PP**						
rs192457787	rs138594252	4	58730210	T	C	*IGFBP7/ROLR2B*	-7.3815	1.5072	9.70E-07	-1.5290	1.0133	0.1313
	rs192457787	4	58795698	A	C		-8.4329	1.6081	1.57E-07	NA	NA	NA
	rs1723953	7	45993668	A	C		1.9482	0.3976	9.61E-07	1.9547	0.3977	8.86E-07
rs150785606	rs11977526	7	46008110	A	G		-0.6674	0.1362	9.53E-07	-0.6658	0.1362	1.02E-06
	rs138317269	7	46989518	T	G		-8.0214	1.6386	9.82E-07	NA	NA	NA
	rs150785606	7	46990467	T	C	*IGFBP3*	8.0378	1.6358	8.94E-07	NA	NA	NA
	rs1723953	7	45993668	A	C		1.9482	0.3976	9.61E-07	NA	NA	NA
rs150785606, rs1723953	rs11977526	7	46008110	A	G		-0.6674	0.1362	9.53E-07	-0.6280	0.1360	3.88E-06
	rs138317269	7	46989518	T	G		-8.0214	1.6386	9.82E-07	NA	NA	NA
	rs150785606	7	46990467	T	C		8.0378	1.6358	8.94E-07	NA	NA	NA
	rs7831012	8	95088353	C	G		-0.9495	0.1622	4.78E-09	-0.0309	0.0928	0.7391
	rs7006922	8	95089653	A	C		1.0757	0.1701	2.56E-10	0.0046	0.0698	0.9472
	rs3018846	8	95090293	A	G		0.7509	0.1487	4.41E-07	0.0406	0.1074	0.7053
	rs2978160	8	95090481	A	C		-0.7518	0.1487	4.25E-07	-0.0415	0.1074	0.6991
	rs2978161	8	95090517	C	G		0.7516	0.1486	4.28E-07	0.0431	0.1075	0.6881
	rs2978165	8	95090906	C	G		-0.7519	0.1483	3.98E-07	-0.0439	0.1071	0.6819
	rs7845175	8	95091144	A	C		0.7526	0.1483	3.87E-07	0.0446	0.1071	0.6772
	8:95091340:T_TG	8	95091340	T	TG		-0.9808	0.1998	9.14E-07	-0.0412	0.1464	0.7782
	rs7013153	8	95092061	A	C		-1.0402	0.1761	3.46E-09	0.0215	0.0859	0.8025
	rs2978143	8	95093235	A	C		0.8404	0.154	4.82E-08	0.0176	0.0975	0.8567
	rs116136580	8	95096074	T	C		-1.0546	0.1738	1.30E-09	0.0406	0.0711	0.5673
	rs6997440	8	95096603	A	G		0.7975	0.1567	3.60E-07	-0.0257	0.1017	0.8007
	rs9987124	8	95096956	A	G		0.7387	0.1489	6.99E-07	-0.0051	0.1028	0.9604
	rs114684581	8	95100302	T	C		-1.0687	0.1745	9.11E-10	0.0343	0.0703	0.6251
	8:95106468:GACAA	8	95106468	G	GACAA		1.2208	0.1834	2.80E-11	NA	NA	NA
	rs2513761	8	95106732	A	T		0.8945	0.1472	1.24E-09	0.1848	0.1054	0.0795
	rs2446846	8	95106768	T	C		0.8956	0.1472	1.16E-09	0.1861	0.1054	0.0775
	rs2513762	8	95106775	A	C		0.8963	0.1472	1.13E-09	0.1868	0.1054	0.0764
	rs2513763	8	95106936	A	C		-0.8954	0.1472	1.18E-09	-0.1860	0.1054	0.0778
	rs2446845	8	95106957	T	G		0.9025	0.1458	6.06E-10	0.2137	0.1064	0.0445
rs7006531	rs2513764	8	95107477	A	C	*CDH17*	-0.6961	0.1402	6.92E-07	-0.1051	0.1110	0.3439
	rs2446844	8	95107693	A	G		-0.904	0.1473	8.38E-10	-0.1935	0.1054	0.0664
	rs2513765	8	95108327	T	C		-0.8948	0.1472	1.21E-09	-0.1842	0.1053	0.0802
	rs2446842	8	95109361	A	G		-0.8936	0.1473	1.31E-09	-0.1807	0.1051	0.0855
	rs2513767	8	95109404	A	G		0.8936	0.1473	1.31E-09	0.1807	0.1051	0.0855
	rs3101283	8	95109499	T	G		0.8936	0.1473	1.31E-09	0.1806	0.1051	0.0856
	rs2446840	8	95109531	T	G		0.8970	0.1472	1.11E-09	0.1850	0.1051	0.0783
	rs2446839	8	95109671	A	G		-0.8934	0.1473	1.33E-09	-0.1804	0.1051	0.0859
	rs2513769	8	95109812	T	C		-0.8928	0.1473	1.37E-09	-0.1799	0.1051	0.0869
	rs2513770	8	95109882	A	C		0.8934	0.1477	1.47E-09	0.1877	0.1067	0.0785
	rs2446838	8	95110200	T	G		0.8908	0.1474	1.53E-09	0.1712	0.1043	0.1006
	rs2446837	8	95110206	T	G		0.8909	0.1474	1.52E-09	0.1713	0.1043	0.1004
	rs2513771	8	95110421	A	T		0.8908	0.1475	1.53E-09	0.1702	0.1043	0.1026
	rs2446836	8	95110444	C	G		-0.8874	0.1474	1.75E-09	-0.1678	0.1043	0.1076
	rs2513772	8	95110454	A	C		-0.8905	0.1474	1.54E-09	-0.1705	0.1042	0.1018
	rs2513773	8	95110512	T	G		0.8904	0.1474	1.55E-09	0.1704	0.1042	0.1020
	rs2446835	8	95110552	T	G		-0.8733	0.1481	3.69E-09	-0.1588	0.1060	0.1339
	rs2446834	8	95110924	T	C		0.8799	0.1473	2.34E-09	0.1607	0.1042	0.1229
	rs2513774	8	95111025	T	C		-0.8516	0.1466	6.29E-09	-0.1490	0.1055	0.1582
	rs2446833	8	95111326	T	C		0.9017	0.1554	6.56E-09	0.0293	0.0905	0.7457
	rs2513775	8	95111617	C	G		0.8650	0.1477	4.68E-09	0.1511	0.1055	0.1521
	rs7006531	8	95110744	A	G		-1.1600	0.1680	5.03E-12	NA	NA	NA
rs113866309	rs113866309	12	66516948	T	C	*LLPH/TMBIM4*	-3.2809	0.6275	1.71E-07	NA	NA	NA
	rs145704323	12	66532537	A	C		3.3753	0.6589	3.01E-07	0.6762	0.4097	0.0989
	rs7403071	15	65048309	A	G		-0.6186	0.126	9.11E-07	-0.0299	0.0611	0.6242
	rs7180635	15	65048912	T	C		-0.6258	0.1246	5.07E-07	-0.0502	0.0625	0.4223
	rs1976112	15	65059220	A	T		-0.6287	0.1245	4.39E-07	-0.0540	0.0626	0.3886
	rs1008917	15	65059651	T	C		0.6188	0.1257	8.61E-07	0.0329	0.0614	0.5915
rs2414856	rs12899738	15	65060028	C	G	*RBPMS2*	0.6188	0.1251	7.48E-07	0.0386	0.0621	0.5335
	rs2414856	15	65072461	A	G		0.6783	0.127	9.18E-08	NA	NA	NA
	rs4777594	15	65076993	A	G		0.6543	0.1277	3.00E-07	NA	NA	NA
	rs2087027	15	65089675	A	G		0.6376	0.13	9.39E-07	0.0503	0.0693	0.4686
	rs11632928	15	65092537	T	C		-0.6381	0.1298	8.86E-07	-0.0805	0.0772	0.2967
