# Supplementary material for: Single-trait and multi-trait genome-wide association analyses identify novel loci for blood pressure in African-ancestry populations
Source: PLoS Genet. 2017 May 12;13(5):e1006728. doi: 10.1371/journal.pgen.1006728 (PMC5446189; doi:10.1371/journal.pgen.1006728)
Supplement: S5 Table — (PDF) [file pgen.1006728.s010.pdf]

S5 Table. 72 independent SNPs with $P < 1.0 \times 10^{-6}$ in discover stage for SBP, DBP, PP, HTN or CPASSOC analysis.

						SBP				DBP				HTN				PP				CPASSOC-SHom	CPASSOC-SHet		
**ID**	**chr**	**pos**	**A1**	**A2**	**EAF**	**Effect**	**StdErr**	**P.value**	**N**	**Effect**	**StdErr**	**P.value**	**N**	**Effect**	**StdErr**	**P.value**	**N**	**Effect**	**StdErr**	**P.value**	**N**	**P.value**	**P.value**	**1KG_African _freq**	**1KG_European _freq**
rs113025995	1	38880204	t	c	0.99	-7.38	4.21	7.90E-02	5212	-2.19	2.04	2.80E-01	10249	-0.96	0.62	1.20E-01	9282	-11.25	2.27	6.80E-07	10249	9.19E-02	2.52E-01	0.99	1
rs36064592	1	114462662	a	g	0.43	0.78	0.18	1.80E-05	31970	0.2	0.11	7.40E-02	31967	0.05	0.02	1.90E-02	28230	0.64	0.13	8.60E-07	31967	1.61E-03	1.51E-04	0.5	0.34
rs12063100	1	188834544	t	g	0.02	2.95	0.88	8.30E-04	19870	1.13	0.53	3.20E-02	19867	-0.19	0.11	7.70E-02	16108	1.88	0.63	3.00E-03	19867	2.37E-01	4.76E-07	0.02	0
rs59922837	2	154175435	a	g	0.01	4.55	1.21	1.70E-04	20703	3.54	0.7	4.50E-07	20700	0.36	0.14	1.10E-02	18451	0.67	0.83	4.20E-01	22314	1.39E-05	3.88E-06	0	0
rs1918172	2	156888500	c	g	0.36	-0.92	0.18	2.90E-07	30714	-0.51	0.11	2.60E-06	30711	-0.03	0.02	9.30E-02	27244	-0.44	0.13	6.00E-04	30711	1.82E-05	1.04E-06	0.41	0.11
rs7602674	2	184538648	t	c	0.39	-0.95	0.19	8.10E-07	30714	-0.36	0.12	2.00E-03	30711	-0.06	0.02	9.70E-03	27230	-0.61	0.14	1.20E-05	30711	6.63E-05	7.00E-06	0.34	0.47
rs62180365	2	192934117	t	g	0.77	1.05	0.2	2.40E-07	31970	0.47	0.12	1.00E-04	31967	0.1	0.02	1.30E-05	27923	0.62	0.14	1.90E-05	31967	2.63E-07	6.41E-07	0.79	0.47
**2:210033781:AT_A**[a]	2	210033781	a	at	0.06	-1.39	0.39	4.00E-04	28554	0.07	0.23	7.50E-01	28551	-0.1	0.05	3.20E-02	20616	**-1.62**	**0.29**	**3.10E-08**	**24802**	4.67E-02	1.68E-06	0.06	0.13
rs737126	2	231896779	a	g	0.24	0.77	0.23	7.20E-04	30841	0.61	0.13	6.00E-06	30838	0.12	0.03	3.30E-06	26789	0.16	0.16	3.30E-01	28703	7.79E-07	1.51E-06	0.28	0.25
rs17042306	3	5381728	a	g	0.88	-1.25	0.28	1.10E-05	31970	-0.79	0.17	3.00E-06	31967	-0.13	0.03	1.30E-04	27728	-0.54	0.21	8.80E-03	30918	6.58E-07	5.69E-06	0.89	0.9
**rs114821199**[a]	3	40965875	a	g	0.01	4.42	1.04	2.30E-05	22648	0.2	0.6	7.40E-01	22645	0.18	0.12	1.40E-01	20396	**4.21**	**0.73**	**1.00E-08**	**22645**	2.92E-02	1.87E-04	0.02	0
**rs7651190**[b]	3	41765955	a	g	0.35	0.04	0.18	8.10E-01	30356	-0.45	0.11	4.20E-05	30353	-0.06	0.02	8.80E-03	26906	0.43	0.13	1.30E-03	30918	7.47E-03	**6.87E-09**	0.28	0.81
**rs147428270**[b]	3	41868721	t	c	0.63	0.09	0.21	6.80E-01	31970	-0.51	0.13	4.60E-05	31967	-0.05	0.02	5.80E-02	28443	0.47	0.15	2.00E-03	30918	2.16E-02	**2.49E-08**	0.57	0.97
rs7372217	3	41990122	a	g	0.34	-0.14	0.19	4.60E-01	29585	-0.55	0.11	9.50E-07	29582	-0.07	0.02	1.40E-03	25731	0.33	0.14	1.60E-02	28533	3.55E-04	8.21E-06	0.29	0.8
rs9864989	3	52684365	t	g	0.03	2.95	0.63	3.20E-06	19178	1.78	0.37	1.90E-06	19175	0.31	0.08	9.70E-05	15416	1.3	0.47	6.00E-03	19740	2.99E-07	2.59E-06	0.05	0
rs7654819	4	11618714	a	c	0.27	-0.6	0.19	2.00E-03	31970	-0.4	0.12	5.20E-04	31967	0.04	0.02	1.10E-01	27987	-0.23	0.14	9.20E-02	31540	9.15E-02	5.77E-08	0.29	0.3
rs192457787	4	58795698	a	c	0.99	-9.35	2.33	6.10E-05	14601	-0.95	1.28	4.60E-01	15515	-0.4	0.29	1.70E-01	14605	-8.43	1.61	1.60E-07	15515	2.60E-02	4.83E-04	1	1
rs150834401	4	103164224	t	c	0.11	1.16	0.28	4.80E-05	31970	0.86	0.17	4.80E-07	31967	0.04	0.03	1.90E-01	27648	0.31	0.2	1.20E-01	31540	9.27E-05	4.11E-06	0.14	0
**rs62312401**[a]	4	116987529	a	g	0.94	1.64	0.4	3.80E-05	30356	**1.31**	**0.24**	**3.50E-08**	**30353**	0.13	0.05	5.00E-03	24655	0.61	0.29	3.50E-02	31540	1.91E-06	3.04E-07	0.96	0.77
rs182783477	4	123592734	c	g	0.98	-2.82	0.7	6.10E-05	29578	-0.45	0.43	2.90E-01	29575	-0.1	0.08	2.20E-01	24849	-2.51	0.51	7.00E-07	29575	2.24E-02	4.86E-04	0.99	1
rs115236533	5	19745715	c	g	0.01	-2.96	1.07	5.50E-03	22648	0.29	0.62	6.30E-01	22645	0.19	0.12	1.30E-01	20396	-2.92	0.74	7.70E-05	24259	9.37E-01	9.16E-07	0.01	0
rs35335431	5	31910483	a	t	0.43	-0.87	0.23	1.20E-04	30356	-0.07	0.14	6.20E-01	30353	-0.03	0.03	2.60E-01	26120	-0.82	0.16	3.50E-07	31967	4.97E-02	9.08E-04	0.44	0.5
rs143572614	5	62024475	c	g	0.99	7.93	2.8	4.60E-03	10582	6.21	1.54	5.80E-05	10579	1.44	0.29	7.80E-07	10636	2.06	1.98	3.00E-01	10579	2.81E-06	3.38E-06	0.99	1
rs189315540	5	108796312	t	c	0.01	6.05	1.8	7.80E-04	16865	0.84	0.99	3.90E-01	17688	0.32	0.18	7.10E-02	18871	6.48	1.21	9.40E-08	17688	2.50E-02	5.49E-03	0.02	0
rs2584077	6	46267046	a	c	0.01	-5.05	1.46	5.30E-04	17352	-4.16	0.83	4.90E-07	17349	-0.24	0.16	1.40E-01	17406	-0.86	1.04	4.10E-01	17349	1.64E-04	4.22E-06	0	0.06
rs9385284	6	123400105	a	g	0.8	-0.47	0.25	6.10E-02	29227	-0.43	0.15	3.20E-03	29224	0.08	0.03	6.40E-03	25126	0.04	0.18	8.40E-01	30838	5.58E-01	5.56E-08	0.82	0.76
rs4487596	6	123407496	t	c	0.8	-0.59	0.23	1.10E-02	30356	-0.4	0.14	4.10E-03	30353	0.05	0.03	5.20E-02	26266	-0.11	0.16	4.90E-01	31967	2.61E-01	3.92E-07	0.81	0.76
**rs76987554**[a,b]	6	134080855	t	c	0.09	**-1.85**	**0.31**	**2.20E-09**	**31970**	-0.91	0.19	1.10E-06	31967	-0.12	0.04	9.20E-04	27480	-0.99	0.22	5.70E-06	31967	8.69E-08	**1.84E-08**	0.09	0
**rs79030490**[a,b]	6	134087689	a	c	0.09	**-1.83**	**0.31**	**3.00E-09**	**31970**	-0.92	0.19	8.70E-07	31967	-0.12	0.04	7.00E-04	27480	-0.96	0.22	1.00E-05	31967	7.01E-08	**2.50E-08**	0.91	1
rs62434120	6	150992370	a	t	0.15	-1.19	0.24	1.10E-06	31970	-0.69	0.15	2.80E-06	31967	-0.1	0.03	4.70E-04	27742	-0.55	0.17	1.50E-03	31967	5.89E-07	2.23E-06	0.17	0.08
rs6969780	7	27159136	c	g	0.3	0.82	0.19	1.70E-05	31155	0.62	0.12	6.90E-08	31152	0.08	0.02	5.30E-04	26701	0.23	0.13	8.90E-02	31152	4.06E-07	5.97E-07	0.35	0.1
**rs10279895**[a,b]	7	27328210	a	g	0.9	1.09	0.29	1.40E-04	31970	0.83	0.17	1.80E-06	31967	**0.19**	**0.03**	**1.80E-08**	**26977**	0.36	0.2	8.10E-02	31967	**2.16E-08**	**3.24E-08**	0.89	1
**rs11563582**[a,b]	7	27351650	a	g	0.13	**1.61**	**0.28**	**7.10E-09**	**30841**	**1.02**	**0.17**	**8.40E-10**	**30838**	0.16	0.03	2.20E-06	26059	0.64	0.19	1.10E-03	30838	**1.51E-10**	**1.08E-09**	0.16	0.08
rs1723953	7	45993668	a	c	0.03	1.87	0.56	9.00E-04	30393	-0.03	0.34	9.20E-01	30390	0.13	0.07	4.60E-02	26631	1.95	0.4	9.60E-07	30390	5.25E-02	3.58E-05	0.03	0
**rs11977526**[b]	7	46008110	a	g	0.34	-0.24	0.19	2.10E-01	29227	0.39	0.12	7.50E-04	29224	0.01	0.02	8.40E-01	25372	-0.67	0.14	9.50E-07	30838	4.10E-01	**4.53E-09**	0.35	0.41
rs150785606	7	46990467	t	c	0.01	9.71	2.34	3.30E-05	10212	1.72	1.27	1.70E-01	11126	0.71	0.29	1.50E-02	10216	8.04	1.64	8.90E-07	11126	2.95E-03	2.69E-04	0	0
7:80293263:A_AAT	7	80293263	a	aatactc	0.85	-0.78	0.29	8.00E-03	25479	-0.86	0.17	5.70E-07	25476	-0.13	0.04	4.10E-04	20999	0.15	0.21	4.80E-01	23862	1.11E-05	4.96E-06	0.82	1
**rs115476423**[b]	7	149199964	c	g	0.94	0.32	0.38	4.10E-01	31970	0.58	0.23	1.20E-02	31967	-0.14	0.05	2.20E-03	27226	-0.22	0.27	4.20E-01	31967	9.42E-01	**4.99E-08**	0.92	1
rs115706913	8	14081817	t	c	0.95	0.77	0.43	7.10E-02	29227	0.36	0.25	1.50E-01	29224	0.26	0.05	2.70E-07	23954	0.33	0.3	2.80E-01	30838	6.40E-04	2.31E-06	0.93	0.97
rs10096908	8	41641557	t	c	0.2	1.03	0.21	1.40E-06	31970	0.47	0.13	2.30E-04	31967	0.11	0.02	1.00E-05	27871	0.55	0.15	2.30E-04	31967	6.21E-07	1.63E-06	0.19	0.07
**rs7006531**[a,b]	8	95110744	a	g	0.85	-0.85	0.24	3.40E-04	31970	0.31	0.14	3.00E-02	31967	-0.02	0.03	5.80E-01	27734	**-1.16**	**0.17**	**5.00E-12**	**31967**	5.63E-01	**7.56E-14**	0.81	1
rs186208701	8	99580116	t	c	0.99	-0.91	0.88	3.00E-01	23777	1.29	0.52	1.20E-02	23774	-0.18	0.1	7.20E-02	21525	-2.12	0.62	6.80E-04	25388	8.82E-01	7.77E-07	0.98	1
rs187821766	8	99741278	t	c	0.02	0.67	0.93	4.70E-01	14176	-1.68	0.56	2.70E-03	14173	0.19	0.1	6.50E-02	12891	2.24	0.7	1.30E-03	14173	8.91E-01	9.25E-08	0.02	0
rs79455834	8	122045324	a	c	0.96	1.75	0.45	1.00E-04	31155	1.1	0.27	5.90E-05	31152	0.25	0.05	1.50E-06	26392	0.73	0.32	2.20E-02	31152	6.55E-07	4.93E-06	0.96	1
rs3866719	8	134932570	t	c	0.82	1.08	0.24	4.00E-06	30714	0.58	0.14	4.20E-05	30711	0.11	0.03	2.20E-05	183238	0.54	0.17	1.30E-03	30711	5.87E-07	5.08E-06	0.87	0.45
**rs78192203**[a]	8	142375073	a	t	0.2	-0.82	0.23	2.60E-04	31970	**-0.77**	**0.14**	**1.30E-08**	**31967**	-0.08	0.03	3.60E-03	27821	-0.15	0.16	3.60E-01	31967	2.33E-06	1.09E-07	0.22	0
rs114334738	9	28165671	t	c	0.94	1.65	0.42	1.00E-04	31970	1.28	0.26	5.70E-07	31967	0.11	0.05	3.40E-02	27226	0.38	0.3	2.00E-01	31967	2.88E-05	4.94E-06	0.92	1
rs71512425	9	28165694	a	g	0.8	0.81	0.23	3.10E-04	31970	0.7	0.13	2.30E-07	31967	0.05	0.03	8.10E-02	27879	0.12	0.16	4.40E-01	31967	6.47E-05	1.99E-06	0.8	0.81
rs115795127	9	85993901	t	c	0.89	0.99	0.28	5.00E-04	31970	0.48	0.17	5.50E-03	31967	0.17	0.03	1.20E-07	27676	0.59	0.2	3.70E-03	31967	5.10E-06	1.05E-06	0.86	1
rs190531342	9	114719568	t	c	0.92	-1.54	0.42	2.10E-04	30356	-1.02	0.25	4.10E-05	30353	-0.23	0.05	2.60E-06	25877	-0.51	0.29	8.50E-02	31967	9.63E-07	5.19E-06	0.93	0.99
rs10123054	9	128452054	t	c	0.65	-0.69	0.18	1.40E-04	31003	-0.57	0.11	2.10E-07	31000	-0.07	0.02	1.50E-03	27582	-0.09	0.13	4.70E-01	31000	2.70E-06	1.85E-06	0.69	0.43
rs28687694	9	128483092	t	c	0.26	0.87	0.2	2.10E-05	28472	0.57	0.12	4.40E-06	28469	0.1	0.02	4.70E-05	24483	0.3	0.15	4.30E-02	30083	5.85E-07	5.06E-06	0.25	0.22
rs78687626	9	133280171	a	g	0.01	-6.13	1.77	5.50E-04	14481	-1.36	1.04	1.90E-01	14478	-0.53	0.2	6.50E-03	13196	-7.35	1.45	4.20E-07	12013	4.33E-03	3.42E-03	0	0.03
10:4699372:GTGTT	10	4699372	g	gtgtt	0.08	-1.55	0.41	1.30E-04	28554	-1.23	0.24	3.20E-07	28551	-0.12	0.05	1.50E-02	20645	-0.29	0.3	3.30E-01	26937	1.43E-05	2.75E-06	0.08	0
10:14646769:T_TCA	10	14646769	t	tca	0.59	-0.91	0.22	3.40E-05	26671	-0.18	0.13	1.60E-01	26668	-0.04	0.03	1.30E-01	20184	-0.79	0.16	6.00E-07	25054	9.92E-03	2.77E-04	0.54	0.59
rs77932157	10	69457644	a	g	0.19	-0.21	0.23	3.50E-01	31970	0.4	0.14	3.50E-03	31967	-0.03	0.03	3.30E-01	27847	-0.58	0.16	2.60E-04	31967	6.77E-01	4.61E-07	0.21	0.02
10:69514659:AAAG_	10	69514659	a	aaag	0.09	-1.14	0.35	1.30E-03	28554	0.17	0.21	4.10E-01	28551	-0.08	0.04	6.40E-02	21024	-1.16	0.26	5.50E-06	26937	1.21E-01	5.40E-07	0.09	0.03
rs11196093	10	114524327	c	g	0.34	-0.72	0.18	7.40E-05	31970	-0.11	0.11	3.00E-01	31967	-0.05	0.02	1.90E-02	28131	-0.64	0.13	6.30E-07	31967	6.10E-03	5.86E-04	0.34	0.1
**rs7941684**[a]	11	5532222	t	g	0.8	**-1.23**	**0.22**	**2.40E-08**	**31970**	-0.54	0.13	4.80E-05	31967	-0.07	0.03	5.00E-03	27872	-0.68	0.16	1.40E-05	31967	3.85E-06	5.82E-07	0.81	0.78
rs145171075	11	80139405	t	g	0.98	4.46	0.91	9.50E-07	24262	1.56	0.52	2.90E-03	24259	0.4	0.1	5.80E-05	22010	3.02	0.64	2.20E-06	24259	5.16E-06	3.92E-06	0.98	1
rs10842715	12	26487183	t	g	0.46	-0.33	0.18	5.70E-02	30841	-0.22	0.1	3.40E-02	30838	-0.1	0.02	4.80E-07	26649	-0.13	0.12	3.00E-01	30838	2.50E-04	4.26E-06	0.5	0.34
12:53049050:AC_A	12	53049050	a	ac	0.86	-1.7	0.43	8.10E-05	17426	-1.18	0.27	1.30E-05	17426	-0.24	0.06	1.10E-05	11700	-0.46	0.31	1.40E-01	15812	7.67E-07	5.49E-06	0.89	0.83
rs113866309	12	66516948	t	c	0.98	-3.73	0.87	1.70E-05	23760	-0.67	0.52	2.00E-01	23757	-0.22	0.1	3.00E-02	22043	-3.28	0.63	1.70E-07	23757	3.91E-03	1.40E-04	0.98	1
rs144058433	13	73013077	t	c	0.14	1.19	0.28	2.40E-05	31970	0.85	0.17	5.00E-07	31967	0.11	0.03	6.30E-04	27732	0.32	0.2	1.10E-01	31967	1.08E-06	4.35E-06	0.12	0.05
rs2414856	15	65072461	a	g	0.51	0.87	0.18	1.00E-06	30714	0.26	0.11	1.80E-02	30711	0.07	0.02	1.10E-03	26575	0.68	0.13	9.20E-08	30711	6.89E-05	8.70E-06	0.61	0.1
rs12445099	16	57890233	a	g	0.67	0.6	0.19	2.00E-03	31970	0.25	0.12	3.10E-02	31967	0.12	0.02	2.20E-07	27573	0.36	0.14	8.70E-03	31967	3.40E-05	1.86E-06	0.61	0.85
rs12149202	16	85700360	a	g	0.11	-1.34	0.3	9.20E-06	31970	-0.69	0.18	1.50E-04	31967	-0.19	0.03	5.10E-08	27644	-0.7	0.22	1.20E-03	31967	9.24E-08	2.98E-07	0.1	0.27
rs17721557	17	27260017	t	c	0.21	1.05	0.21	5.80E-07	30086	0.58	0.13	4.90E-06	30083	0.06	0.02	1.50E-02	25498	0.52	0.15	4.90E-04	30083	6.13E-06	2.22E-06	0.2	0.13
rs114296860	17	51285996	t	c	0.02	2.55	0.65	8.30E-05	30321	1.61	0.39	3.80E-05	30318	-0.04	0.08	5.90E-01	25456	1.11	0.46	1.50E-02	30318	7.16E-03	2.65E-07	0.03	0
rs2832976	21	32037484	t	c	0.16	-1.2	0.26	4.20E-06	31970	-0.33	0.16	3.30E-02	31967	-0.07	0.03	1.70E-02	27732	-0.95	0.19	3.90E-07	31967	6.57E-04	3.54E-05	0.13	0.43
rs62225706	22	26680705	a	c	0.03	-3.97	0.83	1.70E-06	16221	-2.62	0.49	1.10E-07	15169	-0.26	0.1	6.90E-03	12891	-1.61	0.61	8.40E-03	16218	1.17E-06	4.62E-07	0.01	0.11
rs6006767	22	45927045	t	c	0.94	-1.04	0.37	5.20E-03	31970	-1.16	0.23	4.40E-07	30918	-0.08	0.05	9.40E-02	25742	0.05	0.27	8.50E-01	31967	2.63E-04	3.76E-06	0.06	0.11

[a] variants identified with single-trait analysis reaching genome-wide significant threshold of 5.0×10^{-8}

[b] variants identified with multi-traits analysis reaching genome-wide significant threshold of 5.0×10^{-8}

Bold statistics are the corresponding significant statistics for significants of identified variants
