# Supplementary material for: Single-trait and multi-trait genome-wide association analyses identify novel loci for blood pressure in African-ancestry populations
Source: PLoS Genet. 2017 May 12;13(5):e1006728. doi: 10.1371/journal.pgen.1006728 (PMC5446189; doi:10.1371/journal.pgen.1006728)
Supplement: S6 Table — (PDF) [file pgen.1006728.s011.pdf]

S6 Table. Trans-ethnic replication of 72 independent SNPs with P < 1.0 × 10^{-6} in discover stage for SBP, DBP, PP, HTN or CPASSOC analysis.

ID	Chromosome	Physical Position	Allele1	Allele2	EAF	Trait	Discovery				Trans-ethnic Replication				META Analysis (All)			
							Effect	SE	P Value	N	Effect	SE	P Value	N	Effect	SE	P Value	N
rs113025995	1	38880204	t	c	0.99	SBP	-7.3798	4.2054	7.93E-02	5212	NA	NA	NA	NA	NA	NA	NA	NA
						DBP	-2.192	2.0445	2.84E-01	10249	NA	NA	NA	NA	NA	NA	NA	NA
						PP	-11.2545	2.2652	6.75E-07	10249	NA	NA	NA	NA	NA	NA	NA	NA
						HTN	-0.9586	0.6153	1.19E-01	9282	NA	NA	NA	NA	NA	NA	NA	NA
						CPASSOC (SHom)	NA	NA	9.19E-02	5212/10249/9282	NA	NA	NA	NA	NA	NA	9.19E-02	NA
						CPASSOC (SHet)	NA	NA	2.52E-01	5212/10249/9282	NA	NA	NA	NA	NA	NA	2.52E-01	NA
rs36064592	1	114462662	a	g	0.43	SBP	0.7792	0.1819	1.85E-05	31970	-0.0888	0.1512	5.57E-01	35270	0.2658	0.1163	2.22E-02	67240
						DBP	0.1954	0.1094	7.41E-02	31967	-0.0482	0.0926	6.03E-01	35270	0.0535	0.0707	4.49E-01	67237
						PP	0.6374	0.1295	8.64E-07	31967	-0.018	0.1093	8.69E-01	35270	0.2546	0.0835	2.30E-03	67237
						HTN	0.0494	0.021	1.88E-02	28229.5	-0.0336	0.0197	8.85E-02	35532	0.0053	0.0144	7.12E-01	63761.5
						CPASSOC (SHom)	NA	NA	1.61E-03	31970/31976/28229	NA	NA	2.49E-01	35270/35270/35532	NA	NA	5.62E-03	67240/67237/63762
						CPASSOC (SHet)	NA	NA	1.51E-04	31970/31976/28229	NA	NA	2.27E-01	35270/35270/35532	NA	NA	7.21E-04	67240/67237/63762
rs12063100	1	188834544	t	g	0.02	SBP	2.9461	0.8816	8.33E-04	19869.9	NA	NA	NA	NA	NA	NA	NA	NA
						DBP	1.1281	0.5251	3.17E-02	19866.9	NA	NA	NA	NA	NA	NA	NA	NA
						PP	1.8752	0.6315	2.99E-03	19866.9	NA	NA	NA	NA	NA	NA	NA	NA
						HTN	-0.19	0.1074	7.67E-02	16108	NA	NA	NA	NA	NA	NA	NA	NA
						CPASSOC (SHom)	NA	NA	2.37E-01	19869/19869/16108	NA	NA	NA	NA	NA	NA	2.37E-01	NA
						CPASSOC (SHet)	NA	NA	4.76E-07	19869/19869/16108	NA	NA	NA	NA	NA	NA	4.76E-07	NA
rs59922837	2	154175435	a	g	0.01	SBP	4.5541	1.2119	1.71E-04	20703	NA	NA	NA	NA	NA	NA	NA	NA
						DBP	3.5366	0.7007	4.48E-07	20700	NA	NA	NA	NA	NA	NA	NA	NA
						PP	0.6653	0.827	4.21E-01	22314	NA	NA	NA	NA	NA	NA	NA	NA
						HTN	0.3643	0.1433	1.10E-02	18451	NA	NA	NA	NA	NA	NA	NA	NA
						CPASSOC (SHom)	NA	NA	1.39E-05	20703/20700/18451	NA	NA	NA	NA	NA	NA	1.39E-05	NA
						CPASSOC (SHet)	NA	NA	3.88E-06	20703/20700/18451	NA	NA	NA	NA	NA	NA	3.88E-06	NA
rs1918172	2	156888500	c	g	0.36	SBP	-0.9154	0.1785	2.90E-07	30714	-0.1877	0.1908	3.25E-01	37930	-0.5758	0.1303	1.00E-05	68644
						DBP	-0.5079	0.1081	2.61E-06	30711	-0.0854	0.118	4.69E-01	37930	-0.3151	0.0797	7.70E-05	68641
						PP	-0.4356	0.127	6.04E-04	30711	-0.1969	0.1319	1.35E-01	37930	-0.3207	0.0915	4.54E-04	68641
						HTN	-0.0346	0.0206	9.26E-02	27243.7	0.0023	0.0233	9.21E-01	38203	-0.0184	0.0154	2.33E-01	65446.7
						CPASSOC (SHom)	NA	NA	1.82E-05	30714/30711/27243	NA	NA	8.43E-01	37930/37930/37930	NA	NA	7.22E-04	68644/68641/65447
						CPASSOC (SHet)	NA	NA	1.04E-06	30714/30711/27243	NA	NA	8.82E-01	37930/37930/37930	NA	NA	6.84E-05	68644/68641/65447
rs7602674	2	184538648	t	c	0.39	SBP	-0.954	0.1934	8.13E-07	30714	-0.118	0.1458	4.18E-01	36162	-0.4211	0.1164	2.99E-04	66876
						DBP	-0.3614	0.1167	1.96E-03	30711	-0.1669	0.0893	6.18E-02	36162	-0.2387	0.0709	7.64E-04	66873
						PP	-0.6061	0.1387	1.23E-05	30711	0.038	0.1043	7.15E-01	36162	-0.1945	0.0833	1.96E-02	66873
						HTN	-0.0578	0.0223	9.66E-03	27230.4	-0.0077	0.0187	6.81E-01	36416	-0.0284	0.0143	4.76E-02	63646.4
						CPASSOC (SHom)	NA	NA	6.63E-05	30714/30711/27230	NA	NA	9.78E-02	36162/36162/36416	NA	NA	1.40E-04	66876/66873/63646
						CPASSOC (SHet)	NA	NA	7.00E-06	30714/30711/27230	NA	NA	1.73E-01	36162/36162/36416	NA	NA	3.75E-05	66876/66873/63646
rs62180365	2	192934117	t	g	0.77	SBP	1.0454	0.2024	2.41E-07	31970	-0.0247	0.1277	8.47E-01	38978	0.2799	0.108	9.54E-03	70948
						DBP	0.4723	0.1217	1.05E-04	31967	0.0638	0.0785	4.16E-01	38978	0.1838	0.066	5.32E-03	70945
						PP	0.6162	0.1439	1.86E-05	31967	-0.1154	0.0912	2.06E-01	38978	0.0942	0.077	2.22E-01	70945
						HTN	0.1016	0.0233	1.31E-05	27922.8	-0.0192	0.0168	2.51E-01	39247	0.022	0.0136	1.06E-01	67169.8
						CPASSOC (SHom)	NA	NA	2.63E-07	31970/31967/27922	NA	NA	5.44E-01	38978/38978/39247	NA	NA	2.36E-05	70948/70945/67170
						CPASSOC (SHet)	NA	NA	6.41E-07	31970/31967/27922	NA	NA	4.19E-01	38978/38978/39247	NA	NA	3.22E-05	70948/70945/67170
2:210033781:AT_A	2	210033781	a	at	0.06	SBP	-1.3903	0.3929	4.03E-04	28554	-0.0632	0.2028	7.55E-01	49649	-0.3424	0.1802	5.75E-02	78203
						DBP	0.0739	0.2321	7.50E-01	28551	-0.0529	0.1233	6.68E-01	49649	-0.025	0.1089	8.18E-01	78200
						PP	-1.6191	0.2926	3.13E-08	24802	-0.2198	0.1308	9.30E-02	46093	-0.453	0.1194	1.49E-04	70895
						HTN	-0.1021	0.0475	3.17E-02	20615.5	-0.0153	0.0275	5.79E-01	36192	-0.0371	0.0238	1.19E-01	56807.5
						CPASSOC (SHom)	NA	NA	4.67E-02	28554/28551/20615	NA	NA	4.25E-01	49649/49649/36192	NA	NA	1.25E-01	78203/78200/56808
						CPASSOC (SHet)	NA	NA	1.68E-06	28554/28551/20615	NA	NA	7.56E-01	49649/49649/36192	NA	NA	7.61E-05	78203/78200/56808
rs737126	2	231896779	a	g	0.24	SBP	0.7711	0.2281	7.23E-04	30841	0.1842	0.1775	3.00E-01	35171	0.4056	0.1401	3.79E-03	66012
						DBP	0.6105	0.1348	5.97E-06	30838	0.0421	0.1087	6.98E-01	35171	0.2661	0.0846	1.66E-03	66009
						PP	0.1599	0.1628	3.26E-01	28703	0.1227	0.1261	3.30E-01	35171	0.1367	0.0997	1.70E-01	63874
						HTN	0.1225	0.0263	3.27E-06	26789	0.0207	0.0225	3.58E-01	35399	0.0638	0.0171	1.93E-04	62188
						CPASSOC (SHom)	NA	NA	7.79E-07	30841/30838/26789	NA	NA	3.30E-01	35171/35171/35399	NA	NA	1.18E-05	66012/66009/62188
						CPASSOC (SHet)	NA	NA	1.51E-06	30841/30838/26789	NA	NA	6.77E-01	35171/35171/35399	NA	NA	5.76E-05	66012/66009/62188
rs17042306	3	5381728	a	g	0.88	SBP	-1.2546	0.2849	1.06E-05	31970	0.397	0.254	1.18E-01	35125	-0.3344	0.1896	7.78E-02	67095
						DBP	-0.7937	0.17	3.05E-06	31967	0.1412	0.1565	3.67E-01	35125	-0.2877	0.1151	1.25E-02	67092
						PP	-0.538	0.2053	8.77E-03	30918	0.3106	0.1873	9.73E-02	35125	-0.0749	0.1384	5.88E-01	66043
						HTN	-0.126	0.0329	1.30E-04	27727.9	-0.0011	0.0332	9.74E-01	35383	-0.0641	0.0234	6.08E-03	63110.9
						CPASSOC (SHom)	NA	NA	6.58E-07	31970/31967/27727	NA	NA	7.03E-01	35125/35125/35383	NA	NA	2.96E-05	67095/67092/63111
						CPASSOC (SHet)	NA	NA	5.69E-06	31970/31967/27727	NA	NA	4.46E-01	35125/35125/35383	NA	NA	9.94E-05	67095/67092/63111
rs114821199	3	40965875	a	g	0.01	SBP	4.4192	1.0433	2.28E-05	22648	-1.7438	2.3296	4.54E-01	13817	3.3896	0.9522	3.71E-04	36465
						DBP	0.2012	0.6004	7.38E-01	22645	-0.569	1.4464	6.94E-01	13817	0.088	0.5545	8.74E-01	36462
						PP	4.2099	0.7349	1.01E-08	22645	-0.3834	0.8188	6.40E-01	16476	2.1606	0.5469	7.80E-05	39121
						HTN	0.1781	0.121	1.41E-01	20396	-0.2443	0.1603	1.28E-01	16692	0.0248	0.0966	7.97E-01	37088
						CPASSOC (SHom)	NA	NA	2.92E-02	22648/22645/20396	NA	NA	5.10E-01	13817/13817/16692	NA	NA	1.10E-01	36465/36462/37088
						CPASSOC (SHet)	NA	NA	1.87E-04	22648/22645/20396	NA	NA	7.50E-01	13817/13817/16692	NA	NA	4.00E-03	36465/36462/37088
**rs7651190[a]**	3	41765955	a	g	0.35	SBP	0.0447	0.1825	8.07E-01	30356	-0.0501	0.1392	7.19E-01	51662	-0.0152	0.1107	8.91E-01	82018
						DBP	-0.4501	0.1099	4.20E-05	30353	-0.3808	0.0863	1.02E-05	51662	-0.4072	0.0679	1.97E-09	82015
						PP	0.4271	0.1323	1.25E-03	30918	0.125	0.099	2.07E-01	51662	0.2335	0.0793	3.23E-03	82580
						HTN	-0.0563	0.0215	8.79E-03	26905.7	-0.0179	0.0188	3.39E-01	51935	-0.0345	0.0141	1.46E-02	78840.7
						CPASSOC (SHom)	NA	NA	7.47E-03	30356/30353/26905	NA	NA	1.35E-01	51662/51662/51935	NA	NA	1.42E-02	82018/82015/78841
						**CPASSOC (SHet)**	**NA**	**NA**	**6.87E-09**	**30356/30353/26905**	**NA**	**NA**	**1.65E-04**	**51662/51662/51935**	**NA**	**NA**	**9.85E-11**	**82018/82015/78841**
rs147428270	3	41868721	t	c	0.63	SBP	0.0861	0.2097	6.81E-01	31969.9	-0.265	0.2884	3.58E-01	40344	-0.0354	0.1696	8.35E-01	72313.9
						DBP	-0.5125	0.1258	4.59E-05	31966.9	-0.5587	0.1837	2.35E-03	40344	-0.5273	0.1038	3.78E-07	72310.9
						PP	0.4715	0.153	2.05E-03	30917.9	-0.4352	0.2109	3.90E-02	40344	0.1588	0.1238	2.00E-01	71261.9
						HTN	-0.0465	0.0245	5.79E-02	28442.7	-0.0216	0.0401	5.89E-01	40581	-0.0397	0.0209	5.73E-02	69023.7
						CPASSOC (SHom)	NA	NA	2.16E-02	31969/31966/28442	NA	NA	2.81E-01	40344/40344/40581	NA	NA	6.52E-02	72314/72311/69024
						CPASSOC (SHet)	NA	NA	2.49E-08	31969/31966/28442	NA	NA	2.47E-01	40344/40344/40581	NA	NA	1.94E-06	72314/72311/69024
**rs7372217[b]**	3	41990122	a	g	0.34	SBP	-0.1391	0.1887	4.61E-01	29585	-0.0317	0.1393	8.20E-01	51725	-0.0696	0.1121	5.35E-01	81310
						DBP	-0.5514	0.1125	9.50E-07	29582	**-0.4255**	**0.0863**	**8.11E-07**	**51725**	**-0.4721**	**0.0685**	**5.32E-12**	**81307**
						PP	0.3276	0.1354	1.55E-02	28533	0.1578	0.099	1.11E-01	51725	0.2169	0.0799	6.64E-03	80258
						HTN	-0.0697	0.0218	1.39E-03	25730.9	-0.0205	0.0188	2.74E-01	51998	-0.0414	0.0142	3.57E-03	77728.9
						CPASSOC (SHom)	NA	NA	3.55E-04	29585/29582/25730	NA	NA	1.42E-01	51725/51725/51998	NA	NA	1.74E-03	81310/81307/77729
						CPASSOC (SHet)	NA	NA	8.21E-06	29585/29582/25730	**NA**	**NA**	**6.53E-08**	**51725/51725/51998**	**NA**	**NA**	**1.41E-11**	**81310/81307/77729**
rs9864989	3	52684365	t	g	0.03	SBP	2.9529	0.6338	3.18E-06	19178	1.6138	1.5936	3.11E-01	11311	2.77	0.5889	2.56E-06	30489
						DBP	1.7846	0.3749	1.93E-06	19175	0.2876	1.1603	8.04E-01	11311	1.6431	0.3567	4.11E-06	30486
						PP	1.3032	0.4743	6.01E-03	19740	1.504	1.2418	2.26E-01	11311	1.3288	0.4431	2.71E-03	31051
						HTN	0.3123	0.0801	9.70E-05	15416	0.6501	0.2395	6.64E-03	11311	0.3463	0.076	5.15E-06	26727
						CPASSOC (SHom)	NA	NA	2.99E-07	19178/19175/15416	NA	NA	3.48E-01	11311/11311/11311	NA	NA	1.27E-05	30489/30486/26727
						CPASSOC (SHet)	NA	NA	2.59E-06	19178/19175/15416	NA	NA	8.59E-02	11311/11311/11311	NA	NA	1.25E-05	30489/30486/26727
rs7654819	4	11618714	a	c	0.27	SBP	-0.5974	0.193	1.97E-03	31970	-0.0398	0.1612	8.05E-01	37297	-0.269	0.1237	2.97E-02	69267
						DBP	-0.4027	0.1161	5.25E-04	31967	-0.0347	0.098	7.23E-01	37297	-0.1877	0.0749	1.22E-02	69264
						PP	-0.2326	0.138	9.21E-02	31540	0.011	0.1194	9.27E-01	37297	-0.0933	0.0903	3.02E-01	68837
						HTN	0.0358	0.0222	1.06E-01	27987.3	-0.0114	0.0212	5.91E-01	37570	0.0111	0.0153	4.68E-01	65557.3
						CPASSOC (SHom)	NA	NA	9.15E-02	31970/31967/27987	NA	NA	7.05E-01	37297/37297/37570	NA	NA	3.25E-01	69267/69264/65557
						CPASSOC (SHet)	NA	NA	5.77E-08	31970/31967/27987	NA	NA	9.68E-01	37297/37297/37570	NA	NA	7.08E-06	69267/69264/65557
rs192457787	4	58795698	a	c	0.99	SBP	-9.3475	2.3309	6.07E-05	14601	NA	NA	NA	NA	NA	NA	NA	NA
						DBP	-0.9472	1.2754	4.58E-01	15515	NA	NA	NA	NA	NA	NA	NA	NA
						PP	-8.4329	1.6081	1.57E-07	15515	NA	NA	NA	NA	NA	NA	NA	NA
						HTN	-0.3983	0.2886	1.68E-01	14605	NA	NA	NA	NA	NA	NA	NA	NA
						CPASSOC (SHom)	NA	NA	2.60E-02	14601/15515/14605	NA	NA	1.00E+00	NA	NA	NA	2.60E-02	NA
						CPASSOC (SHet)	NA	NA	4.83E-04	14601/15515/14605	NA	NA	1.00E+00	NA	NA	NA	4.83E-04	NA
rs150834401	4	103164224	t	c	0.11	SBP	1.156	0.2842	4.75E-05	31970	0.0365	0.845	9.66E-01	24420	1.0422	0.2694	1.09E-04	56390
						DBP	0.8647	0.1717	4.77E-07	31967	0.1027	0.6102	8.66E-01	24420	0.8088	0.1653	9.91E-07	56387
						PP	0.3146	0.2016	1.19E-01	31540	-0.1388	0.6593	8.33E-01	24420	0.2758	0.1928	1.53E-01	55960
						HTN	0.0434	0.0329	1.88E-01	27648.3	0.0609	0.1311	6.42E-01	24420	0.0444	0.0319	1.64E-01	52068.3
						CPASSOC (SHom)	NA	NA	9.27E-05	31970/31967/27648	NA	NA	9.50E-01	24420/24420/24420	NA	NA	9.56E-03	56390/56387/52068
						CPASSOC (SHet)	NA	NA	4.11E-06	31970/31967/27648	NA	NA	4.90E-01	24420/24420/24420	NA	NA	8.51E-05	56390/56387/52068
rs62312401	4	116987529	a	g	0.94	SBP	1.6367	0.3976	3.85E-05	30356	0.1851	0.173	2.85E-01	46511	0.4162	0.1587	8.71E-03	76867
						DBP	1.3123	0.238	3.50E-08	30353	0.0806	0.1065	4.49E-01	46511	0.2861	0.0972	3.25E-03	76864
						PP	0.6084	0.2881	3.47E-02	31540	0.1673	0.1268	1.87E-01	46511	0.2389	0.116	3.95E-02	78051
						HTN	0.1321	0.0471	5.00E-03	24654.7	0.0187	0.0229	4.14E-01	46726	0.0403	0.0206	5.01E-02	71380.7
						CPASSOC (SHom)	NA	NA	1.91E-06	30356/30353/24654	NA	NA	1.09E-01	46511/46511/46726	NA	NA	1.29E-05	76867/76864/71381
						CPASSOC (SHet)	NA	NA	3.04E-07	30356/30353/24654	NA	NA	4.97E-01	46511/46511/46726	NA	NA	2.27E-05	76867/76864/71381
rs182783477	4	123592734	c	g	0.98	SBP	-2.8224	0.7041	6.12E-05	29578	-2.2084	17.0184	8.97E-01	3428	-2.8214	0.7035	6.06E-05	33006
						DBP	-0.4527	0.4314	2.94E-01	29575	-0.6944	9.9927	9.45E-01	3428	-0.4531	0.431	2.93E-01	33003
						PP	-2.5118	0.5062	6.97E-07	29575	-1.514	13.9172	9.13E-01	3428	-2.5105	0.5059	6.95E-07	33003

[illegible]	4	[illegible]	c	g	[illegible]	HTN	-0.1031	0.0835	2.17E-01	24849	-1.3067	32.4195	9.68E-01	3428	-0.1031	0.0835	2.17E-01	28277
						CPASSOC (SHom)	NA	NA	2.24E-02	29578/29575/24849	NA	NA	9.32E-01	3428/3428/3428	NA	NA	1.02E-01	33006/33003/28277
						CPASSOC (SHet)	NA	NA	4.86E-04	29578/29575/24849	NA	NA	9.97E-01	3428/3428/3428	NA	NA	4.18E-03	33006/33003/28277
rs115236533	5	19745715	c	g	0.01	SBP	-2.9626	1.0665	5.47E-03	22648	NA	NA	NA	NA	NA	NA	NA	NA
						DBP	0.2942	0.617	6.34E-01	22645	NA	NA	NA	NA	NA	NA	NA	NA
						PP	-2.9243	0.7398	7.72E-05	24259	NA	NA	NA	NA	NA	NA	NA	NA
						HTN	0.1867	0.1245	1.34E-01	20396	NA	NA	NA	NA	NA	NA	NA	NA
						CPASSOC (SHom)	NA	NA	9.37E-01	22648/22645/20396	NA	NA	NA	NA	NA	NA	9.37E-01	NA
						CPASSOC (SHet)	NA	NA	9.16E-07	22648/22645/20396	NA	NA	NA	NA	NA	NA	9.16E-07	NA
rs35335431	5	31910483	a	t	0.43	SBP	-0.8718	0.2263	1.17E-04	30355.7	-0.1061	0.1677	5.27E-01	34243	-0.3777	0.1348	5.07E-03	64598.7
						DBP	-0.0673	0.136	6.21E-01	30352.7	-0.0646	0.102	5.26E-01	34243	-0.0656	0.0816	4.22E-01	64595.7
						PP	-0.8169	0.1603	3.46E-07	31966.7	-0.0615	0.1244	6.21E-01	34243	-0.3455	0.0983	4.40E-04	66209.7
						HTN	-0.0302	0.027	2.64E-01	26120.3	0.0087	0.0213	6.81E-01	34423	-0.0062	0.0167	7.12E-01	60543.3
						CPASSOC (SHom)	NA	NA	4.97E-02	30355/30352/26120	NA	NA	9.50E-01	34423/34423/34423	NA	NA	4.69E-01	64599/64596/60543
						CPASSOC (SHet)	NA	NA	9.08E-04	30355/30352/26120	NA	NA	9.99E-02	34423/34423/34423	NA	NA	1.78E-03	64599/64596/60543
rs143572614	5	62024475	c	g	0.99	SBP	7.9343	2.7977	4.57E-03	10582	NA	NA	NA	NA	NA	NA	NA	NA
						DBP	6.2089	1.5436	5.76E-05	10579	NA	NA	NA	NA	NA	NA	NA	NA
						PP	2.0587	1.9795	2.98E-01	10579	NA	NA	NA	NA	NA	NA	NA	NA
						HTN	1.4411	0.2917	7.81E-07	10636	NA	NA	NA	NA	NA	NA	NA	NA
						CPASSOC (SHom)	NA	NA	2.81E-06	10582/10579/10636	NA	NA	NA	NA	NA	NA	2.81E-06	NA
						CPASSOC (SHet)	NA	NA	3.38E-06	10582/10579/10636	NA	NA	NA	NA	NA	NA	3.38E-06	NA
rs189315540	5	108796312	t	c	0.01	SBP	6.0518	1.8012	7.80E-04	16865	0.1749	2.0122	9.31E-01	13718	3.4375	1.3421	1.04E-02	30583
						DBP	0.8433	0.9855	3.92E-01	17688	0.5288	1.3344	6.92E-01	13718	0.7323	0.7927	3.56E-01	31406
						PP	6.4812	1.2143	9.44E-08	17688	-0.3142	1.2874	8.07E-01	13718	3.2819	0.8834	2.03E-04	31406
						HTN	0.3229	0.1787	7.08E-02	18871	0.0832	0.321	7.96E-01	13974	0.2662	0.1561	8.82E-02	32845
						CPASSOC (SHom)	NA	NA	2.50E-02	16865/17699/18871	NA	NA	7.64E-01	13718/13718/13974	NA	NA	9.46E-02	30583/31406/32845
						CPASSOC (SHet)	NA	NA	5.49E-03	16865/17699/18871	NA	NA	NA	13718/13718/13974	NA	NA	5.49E-03	30583/31406/32845
rs2584077	6	46267046	a	c	0.01	SBP	-5.0528	1.4592	5.35E-04	17352	0.4931	0.4712	2.95E-01	36925	-0.0305	0.4484	9.46E-01	54277
						DBP	-4.1562	0.8261	4.87E-07	17349	0.5131	0.2855	7.23E-02	36925	0.0149	0.2698	9.56E-01	54274
						PP	-0.857	1.0407	4.10E-01	17349	0.1266	0.3656	7.29E-01	36925	0.0185	0.345	9.57E-01	54274
						HTN	-0.236	0.1614	1.44E-01	17406	0.1471	0.0594	1.33E-02	37187	0.1014	0.0558	6.91E-02	54593
						CPASSOC (SHom)	NA	NA	1.64E-04	17352/17349/17406	NA	NA	5.81E-02	36925/36925/37187	NA	NA	1.76E-04	54277/54274/54593
						CPASSOC (SHet)	NA	NA	4.22E-06	17352/17349/17406	NA	NA	2.11E-01	36925/36925/37187	NA	NA	3.02E-05	54277/54274/54593
rs9385284	6	123400105	a	g	0.80	SBP	-0.4677	0.2499	6.13E-02	29227	0.0645	0.1321	6.25E-01	48897	-0.0517	0.1168	6.58E-01	78124
						DBP	-0.4332	0.1471	3.24E-03	29224	0.0065	0.0817	9.36E-01	48897	-0.0971	0.0714	1.74E-01	78121
						PP	0.0352	0.1762	8.42E-01	30838	0.0606	0.0927	5.13E-01	48897	0.0551	0.0821	5.02E-01	79735
						HTN	0.0784	0.0287	6.40E-03	25125.7	0.0151	0.0186	4.17E-01	49143	0.0338	0.0156	3.02E-02	74268.7
						CPASSOC (SHom)	NA	NA	5.58E-01	29227/29224/25125	NA	NA	1.22E-01	48897/48897/49143	NA	NA	1.89E-01	78124/78121/74269
						CPASSOC (SHet)	NA	NA	2.56E-08	29227/29224/25125	NA	NA	2.74E-01	48897/48897/49143	NA	NA	1.09E-06	78124/78121/74269
rs4487596	6	123407496	t	c	0.80	SBP	-0.585	0.2296	1.08E-02	30356	-0.1053	0.1538	4.94E-01	37389	-0.2539	0.1278	4.69E-02	67745
						DBP	-0.3962	0.1381	4.11E-03	30353	-0.0794	0.0947	4.02E-01	37389	-0.1807	0.0781	2.07E-02	67742
						PP	-0.1145	0.1642	4.85E-01	31967	-0.015	0.1079	8.89E-01	37389	-0.045	0.0902	6.18E-01	69356
						HTN	0.0516	0.0265	5.18E-02	26266.3	-0.0106	0.0193	5.83E-01	37645	0.0109	0.0156	4.84E-01	63911.3
						CPASSOC (SHom)	NA	NA	2.61E-01	30356/30353/26266	NA	NA	6.79E-01	37389/37389/37645	NA	NA	5.44E-01	67745/67742/63911
						CPASSOC (SHet)	NA	NA	3.92E-07	30356/30353/26266	NA	NA	9.65E-01	37389/37389/37645	NA	NA	3.79E-05	67745/67742/63911
**rs76987554**[a,c]	6	134080855	t	c	0.09	**SBP**	**-1.8492**	**0.309**	**2.17E-09**	**31969.9**	**-1.2266**	**0.5259**	**1.97E-02**	**30327**	**-1.6894**	**0.2664**	**2.28E-10**	**62296.9**
						DBP	-0.905	0.1859	1.12E-06	31966.9	-0.3548	0.3489	3.09E-01	29101	-0.7833	0.1641	1.80E-06	61067.9
						PP	-0.9899	0.2182	5.73E-06	31966.9	-0.4173	0.4025	3.00E-01	29101	-0.8598	0.1918	7.38E-06	61067.9
						HTN	-0.1184	0.0357	9.16E-04	27480	-0.1135	0.0781	1.46E-01	29036	-0.1176	0.0325	2.94E-04	56516
						CPASSOC (SHom)	NA	NA	8.69E-08	31969/31966/27480	NA	NA	1.95E-01	30327/29101/29036	NA	NA	8.66E-07	62297/61068/56516
						CPASSOC (SHet)	NA	NA	1.84E-08	31969/31966/27480	NA	NA	1.22E-01	30327/29101/29036	NA	NA	1.22E-07	62297/61068/56516
rs79030490	6	134087689	a	c	0.09	SBP	-1.8344	0.3091	2.96E-09	31969.9	-6.4041	4.0623	1.15E-01	14157	-1.8607	0.3082	1.57E-09	46126.9
						DBP	-0.9154	0.186	8.65E-07	31966.9	-4.2993	2.5996	9.82E-02	14157	-0.9326	0.1855	4.98E-07	46123.9
						PP	-0.9636	0.2185	1.03E-05	31966.9	-4.4041	3.2621	1.77E-01	14157	-0.979	0.218	7.11E-06	46123.9
						HTN	-0.1212	0.0357	6.98E-04	27479.7	0.0771	0.7941	9.23E-01	14157	-0.1208	0.0357	7.06E-04	41636.7
						CPASSOC (SHom)	NA	NA	7.01E-08	31969/31966/27479	NA	NA	2.85E-01	14157/14157/14157	NA	NA	3.74E-07	46127/46124/41637
						CPASSOC (SHet)	NA	NA	2.50E-08	31969/31966/27479	NA	NA	1.67E-01	14157/14157/14157	NA	NA	8.49E-08	46127/46124/41637
**rs62434120**[b]	6	150992370	a	t	0.15	SBP	-1.1882	0.2434	1.05E-06	31970	**-0.7336**	**0.276**	**2.70E-03**	**37744**	**-0.9925**	**0.1726**	**5.72E-09**	**69714**
						DBP	-0.686	0.1465	2.82E-06	31967	-0.4249	0.1683	1.16E-02	37744	-0.5734	0.1105	2.11E-07	69711
						PP	-0.5495	0.1728	1.48E-03	31967	-0.2061	0.2021	3.08E-01	37744	-0.4045	0.1313	2.07E-03	69711
						HTN	-0.0967	0.0276	4.70E-04	27741.9	-0.0817	0.0367	2.61E-02	38015	-0.0913	0.0221	3.51E-05	65756.9
						CPASSOC (SHom)	NA	NA	5.89E-07	31970/31967/27742	NA	NA	2.42E-02	37744/37744/38015	NA	NA	4.75E-07	69714/69711/69757
						CPASSOC (SHet)	NA	NA	2.23E-06	31970/31967/27742	NA	NA	1.57E-01	37744/37744/38015	NA	NA	1.23E-05	69714/69711/69757
**rs6969780**[b]	7	27159136	c	g	0.30	SBP	0.8198	0.1903	1.65E-05	31155	**0.6075**	**0.1521**	**6.46E-05**	**54245**	**0.6902**	**0.1188**	**6.23E-09**	**85400**
						DBP	0.6214	0.1152	6.95E-08	31152	**0.3501**	**0.0945**	**2.12E-04**	**54245**	**0.4593**	**0.0731**	**3.27E-10**	**85397**
						PP	0.2282	0.1343	8.94E-02	31152	0.3158	0.1123	4.90E-03	51578	0.2798	0.0861	1.16E-03	82730
						HTN	0.0779	0.0225	5.26E-04	26700.8	0.0719	0.0209	5.84E-04	54452	0.0747	0.0153	1.08E-06	81152.8
						CPASSOC (SHom)	NA	NA	4.06E-07	31155/31152/26700	**NA**	**NA**	**4.29E-04**	**54245/54245/54452**	**NA**	**NA**	**9.92E-09**	**85400/85397/81153**
						CPASSOC (SHet)	NA	NA	5.97E-07	31155/31152/26700	NA	NA	5.80E-03	54245/54245/54452	NA	NA	2.43E-07	85400/85397/81153
rs10279895	7	27328210	a	g	0.90	SBP	1.0947	0.2879	1.43E-04	31970	0.3865	0.6401	5.46E-01	29101	0.9755	0.2626	2.03E-04	61071
						DBP	0.8302	0.1738	1.78E-06	31967	0.3721	0.3931	3.44E-01	29101	0.7553	0.159	2.02E-06	61068
						PP	0.3565	0.2044	8.11E-02	31967	0.3871	0.6392	5.45E-01	26435	0.3593	0.1947	6.49E-02	58402
						HTN	0.1894	0.0337	1.84E-08	26977.3	0.1252	0.0885	1.57E-01	29036	0.1813	0.0315	8.63E-09	56013.3
						CPASSOC (SHom)	NA	NA	2.16E-08	31970/31967/26977	NA	NA	4.19E-01	29101/29101/29036	NA	NA	6.52E-07	61071/61068/56013
						CPASSOC (SHet)	NA	NA	3.24E-08	31970/31967/26977	NA	NA	4.93E-01	29101/29101/29036	NA	NA	1.18E-06	61071/61068/56013
**rs11563582**[a]	7	27351650	a	g	0.13	**SBP**	**1.6125**	**0.2786**	**7.09E-09**	**30841**	**0.7026**	**0.1991**	**4.17E-04**	**53978**	**1.0102**	**0.162**	**4.47E-10**	**84819**
						**DBP**	**1.0151**	**0.1654**	**8.45E-10**	**30838**	**0.4707**	**0.1233**	**1.35E-04**	**53978**	**0.6652**	**0.0989**	**1.71E-11**	**84816**
						PP	0.6381	0.1948	1.06E-03	30838	0.3345	0.1491	2.49E-02	51319	0.4466	0.1184	1.62E-04	82157
						HTN	0.1566	0.0331	2.24E-06	26058.5	0.0886	0.0266	8.82E-04	54179	0.1153	0.0208	2.74E-08	80237.5
						**CPASSOC (SHom)**	**NA**	**NA**	**1.51E-10**	**30841/30838/26058**	**NA**	**NA**	**7.98E-04**	**53978/53978/54179**	**NA**	**NA**	**1.88E-11**	**84819/84816/80238**
						**CPASSOC (SHet)**	**NA**	**NA**	**1.08E-09**	**30841/30838/26058**	**NA**	**NA**	**9.41E-03**	**53978/53978/54179**	**NA**	**NA**	**1.84E-09**	**84819/84816/80238**
rs1723953	7	45993668	a	c	0.03	SBP	1.8689	0.5628	8.98E-04	30393	2.8663	7.4379	7.00E-01	10729	1.8746	0.5612	8.37E-04	41122
						DBP	-0.0322	0.3358	9.24E-01	30390	4.5177	5.6277	4.22E-01	10729	-0.0161	0.3352	9.62E-01	41119
						PP	1.9482	0.3976	9.61E-07	30390	0.9821	6.7442	8.84E-01	10729	1.9449	0.3969	9.59E-07	41119
						HTN	0.1316	0.0658	4.56E-02	26631	1.7283	1.2979	1.83E-01	10729	0.1357	0.0657	3.89E-02	37360
						CPASSOC (SHom)	NA	NA	5.25E-02	30393/30390/26631	NA	NA	2.92E-01	10729/10729/10729	NA	NA	7.93E-02	41122/41119/37360
						CPASSOC (SHet)	NA	NA	3.58E-05	30393/30390/26631	NA	NA	5.53E-01	10729/10729/10729	NA	NA	2.34E-04	41122/41119/37360
**rs11977526**[a]	7	46008110	a	g	0.34	SBP	-0.2433	0.1943	2.11E-01	29227	-0.1507	0.122	2.17E-01	48305	-0.1769	0.1033	8.69E-02	77532
						DBP	0.3881	0.1152	7.52E-04	29224	0.2399	0.0736	1.11E-03	50971	0.2829	0.062	5.08E-06	80195
						PP	-0.6674	0.1362	9.53E-07	30838	-0.2346	0.0865	6.71E-03	50971	-0.359	0.073	8.84E-07	81809
						HTN	-0.0044	0.0224	8.44E-01	25371.9	-0.0064	0.0164	6.97E-01	49723	-0.0057	0.0132	6.67E-01	75094.9
						CPASSOC (SHom)	NA	NA	4.10E-01	29227/29224/25371	NA	NA	1.60E-01	48305/50971/49723	NA	NA	1.92E-01	77532/80195/75095
						**CPASSOC (SHet)**	**NA**	**NA**	**4.53E-09**	**29227/29224/25371**	**NA**	**NA**	**2.93E-09**	**48305/50971/49723**	**NA**	**NA**	**7.34E-16**	**77532/80195/75095**
rs150785606	7	46990467	t	c	0.01	SBP	9.7112	2.3396	3.31E-05	10212	NA	NA	NA	NA	NA	NA	NA	NA
						DBP	1.7188	1.2668	1.75E-01	11126	NA	NA	NA	NA	NA	NA	NA	NA
						PP	8.0378	1.6358	8.94E-07	11126	NA	NA	NA	NA	NA	NA	NA	NA
						HTN	0.706	0.2909	1.52E-02	10216	NA	NA	NA	NA	NA	NA	NA	NA
						CPASSOC (SHom)	NA	NA	2.95E-03	10212/11126/10216	NA	NA	NA	NA	NA	NA	2.95E-03	NA
						CPASSOC (SHet)	NA	NA	2.69E-04	10212/11126/10216	NA	NA	NA	NA	NA	NA	2.69E-04	NA
7:80293263:A_AAT	7	80293263	a	aatactc	0.85	SBP	-0.7788	0.2936	7.98E-03	25479	4.1393	4.2589	3.31E-01	18445	-0.7555	0.2929	9.90E-03	43924
						DBP	-0.8585	0.1717	5.70E-07	25476	3.408	2.5957	1.89E-01	18445	-0.8399	0.1713	9.47E-07	43921
						PP	0.1506	0.2118	4.77E-01	23862	1.9541	3.1301	5.32E-01	18445	0.1588	0.2113	4.52E-01	42307
						HTN	-0.1255	0.0355	4.08E-04	20999	1.244	0.919	1.76E-01	9684	-0.1235	0.0355	5.01E-04	30683
						CPASSOC (SHom)	NA	NA	1.11E-05	25479/25476/20999	NA	NA	2.31E-01	18445/18445/9684	NA	NA	3.56E-05	43924/43921/30683
						CPASSOC (SHet)	NA	NA	4.96E-06	25479/25476/20999	NA	NA	5.39E-01	18445/18445/9684	NA	NA	3.70E-05	43924/43921/30683
rs115476423	7	149199964	c	g	0.94	SBP	0.3162	0.3841	4.10E-01	31970	1.1005	1.3607	4.19E-01	16751	0.3741	0.3697	3.12E-01	48721
						DBP	0.5803	0.2305	1.18E-02	31967	0.1553	0.8469	8.55E-01	16751	0.551	0.2224	1.32E-02	48718
						PP	-0.2183	0.2719	4.22E-01	31967	0.4542	0.9641	6.38E-01	16751	-0.1688	0.2617	5.19E-01	48718
						HTN	-0.1376	0.045	2.21E-03	27226.2	0.2006	0.193	2.99E-01	16751	-0.1202	0.0438	6.11E-03	43977.2
						CPASSOC (SHom)	NA	NA	9.42E-01	31970/31967/27226	NA	NA	5.98E-01	16751/16751/16751	NA	NA	8.23E-01	48721/48718/43977
						CPASSOC (SHet)	NA	NA	4.99E-08	31970/31967/27226	NA	NA	9.15E-01	16751/16751/16751	NA	NA	5.17E-06	48721/48718/43977
rs115706913	8	14081817	t	c	0.95	SBP	0.7716	0.4281	7.15E-02	29227	0.0828	0.558	8.82E-01	23914	0.5164	0.3397	1.28E-01	53141
						DBP	0.3639	0.2528	1.50E-01	29224	0.1031	0.334	7.58E-01	23914	0.2689	0.2016	1.82E-01	53138
						PP	0.325	0.2986	2.76E-01	30838	-0.2151	0.4311	6.18E-01	23914	0.1499	0.2455	5.41E-01	54752
						HTN	0.2609	0.0507	2.69E-07	23954.4	0.1124	0.0733	1.25E-01	23914	0.2128	0.0417	3.32E-07	47868.4
						CPASSOC (SHom)	NA	NA	6.40E-04	29227/29224/23954	NA	NA	3.73E-01	23914/23914/23914	NA	NA	2.23E-03	53141/53148/47868
						CPASSOC (SHet)	NA	NA	2.31E-06	29227/29224/23954	NA	NA	4.29E-01	23914/23914/23914	NA	NA	1.47E-05	53141/53148/47868
rs10096908	8	41641557	t	c	0.20	SBP	1.0253	0.2124	1.39E-06	31970	0.4149	0.3087	1.79E-01	37570	0.8292	0.175	2.15E-06	69540
						DBP	0.4726	0.1282	2.27E-04	31967	0.2285	0.1869	2.22E-01	37570	0.3945	0.1057	1.90E-04	69537
						PP	0.5538	0.1504	2.31E-04	31967	0.1631	0.2307	4.80E-01	37570	0.4373	0.126	5.19E-04	69537
						HTN	0.1078	0.0244	1.02E-05	27870.7	0.0595	0.04	1.37E-01	37839	0.0947	0.0208	5.47E-06	65709.7
						CPASSOC (SHom)	NA	NA	6.21E-07	31970/31967/27870	NA	NA	9.56E-02	37570/37570/37839	NA	NA	2.23E-06	69540/69537/65910

						CPASSOC (SHet)	NA	NA	1.63E-06	31970/31967/27870	NA	NA	4.15E-01	37570/37570/37839	NA	NA	3.03E-05	69540/69537/65910
**rs7006531[a]**	8	95110744	a	g	0.85	SBP	-0.8494	0.2372	3.43E-04	31970	-0.7612	0.4429	8.57E-02	40920	-0.8297	0.2091	7.25E-05	72890
						DBP	0.3108	0.1433	3.01E-02	31967	0.457	0.3875	2.38E-01	38254	0.3284	0.1344	1.46E-02	70221
						**PP**	**-1.1601**	**0.168**	**5.03E-12**	**31967**	-0.5275	0.3176	9.67E-02	40920	**-1.0218**	**0.1485**	**5.96E-12**	**72887**
						HTN	-0.0152	0.0274	5.78E-01	27733.8	0.0403	0.0882	6.48E-01	38526	-0.0103	0.0262	6.94E-01	66259.8
						CPASSOC (SHom)	NA	NA	5.63E-01	31970/31967/27733	NA	NA	9.11E-01	40920/38254/38526	NA	NA	9.22E-01	72890/70221/66260
						**CPASSOC (SHet)**	**NA**	**NA**	**7.56E-14**	**31970/31967/27733**	**NA**	**NA**	**6.13E-03**	**40920/38254/38526**	**NA**	**NA**	**2.19E-13**	**72890/70221/66260**
rs186208701	8	99580116	t	c	0.99	SBP	-0.9056	0.8754	3.01E-01	23777	NA	NA	NA	NA	NA	NA	NA	NA
						DBP	1.291	0.5168	1.25E-02	23774	NA	NA	NA	NA	NA	NA	NA	NA
						PP	-2.117	0.623	6.79E-04	25388	NA	NA	NA	NA	NA	NA	NA	NA
						HTN	-0.1806	0.1002	7.15E-02	21525	NA	NA	NA	NA	NA	NA	NA	NA
						CPASSOC (SHom)	NA	NA	8.82E-01	23777/23774/21525	NA	NA	NA	NA	NA	NA	8.82E-01	NA
						CPASSOC (SHet)	NA	NA	7.77E-07	23777/23774/21525	NA	NA	NA	NA	NA	NA	7.77E-07	NA
rs187821766	8	99741278	t	c	0.02	SBP	0.6681	0.9348	4.75E-01	14176	NA	NA	NA	NA	NA	NA	NA	NA
						DBP	-1.6757	0.5577	2.66E-03	14173	NA	NA	NA	NA	NA	NA	NA	NA
						PP	2.2392	0.6959	1.29E-03	14173	NA	NA	NA	NA	NA	NA	NA	NA
						HTN	0.1939	0.1049	6.45E-02	12891	NA	NA	NA	NA	NA	NA	NA	NA
						CPASSOC (SHom)	NA	NA	8.91E-01	14176/14173/12891	NA	NA	NA	NA	NA	NA	8.91E-01	NA
						CPASSOC (SHet)	NA	NA	9.25E-08	14176/14173/12891	NA	NA	NA	NA	NA	NA	9.25E-08	NA
rs79455834	8	122045324	a	c	0.96	SBP	1.7549	0.4515	1.02E-04	31155	0.1733	1.275	8.92E-01	27691	1.5787	0.4256	2.08E-04	58846
						DBP	1.1009	0.274	5.85E-05	31152	0.8366	0.8333	3.15E-01	27691	1.0751	0.2603	3.62E-05	58843
						PP	0.7324	0.319	2.17E-02	31152	-0.7314	0.9673	4.50E-01	27691	0.5888	0.3029	5.20E-02	58843
						HTN	0.2535	0.0527	1.48E-06	26392.1	0.125	0.1893	5.09E-01	27958	0.2443	0.0508	1.50E-06	54350.1
						CPASSOC (SHom)	NA	NA	6.55E-07	31155/31152/26392	NA	NA	3.26E-01	27691/27691/27958	NA	NA	9.95E-06	58846/58843/54350
						CPASSOC (SHet)	NA	NA	4.93E-06	31155/31152/26392	NA	NA	7.52E-01	27691/27691/27958	NA	NA	1.91E-04	58846/58843/54350
rs3866719	8	134932570	t	c	0.82	SBP	1.084	0.235	3.98E-06	30714	-0.0146	0.1309	9.11E-01	36781	0.2456	0.1144	3.18E-02	67495
						DBP	0.583	0.1423	4.21E-05	30711	0.0827	0.0802	3.03E-01	36781	0.2033	0.0699	3.62E-03	67492
						PP	0.5419	0.1685	1.30E-03	30711	-0.0892	0.0937	3.41E-01	36781	0.06	0.0819	4.64E-01	67492
						HTN	0.1148	0.0271	2.22E-05	18238	-0.0102	0.0172	5.53E-01	37024	0.0256	0.0145	7.71E-02	55262
						CPASSOC (SHom)	NA	NA	5.87E-07	30714/30711/18238	NA	NA	8.24E-01	36781/36781/37024	NA	NA	3.58E-05	67495/67492/55262
						CPASSOC (SHet)	NA	NA	5.08E-06	30714/30711/18238	NA	NA	7.18E-01	36781/36781/37024	NA	NA	1.81E-04	67495/67492/55262
**rs78192203[a]**	8	142375073	a	t	0.20	SBP	-0.8242	0.2257	2.61E-04	31970	-2.0961	0.5973	4.50E-04	30714	-0.9831	0.2111	3.22E-06	62684
						**DBP**	**-0.7722**	**0.1357**	**1.26E-08**	**31967**	**-1.3428**	**0.3686**	**2.69E-04**	**30714**	**-0.8403**	**0.1273**	**4.15E-11**	**62681**
						PP	-0.1477	0.1597	3.55E-01	31967	-1.3663	0.4247	1.29E-03	39423	-0.2987	0.1495	4.57E-02	71390
						HTN	-0.0756	0.026	3.58E-03	27820.6	-0.2519	0.0857	3.28E-03	30714	-0.0905	0.0249	2.77E-04	58534.6
						CPASSOC (SHom)	NA	NA	2.33E-06	31970/31967/27820	NA	NA	1.66E-03	30714/30714/30714	NA	NA	1.42E-07	62684/62681/58535
						CPASSOC (SHet)	NA	NA	1.09E-07	31970/31967/27820	NA	NA	2.85E-03	30714/30714/30714	NA	NA	1.69E-08	62684/62681/58535
rs114334738	9	28165671	t	c	0.94	SBP	1.6453	0.4236	1.03E-04	31970	3.7745	5.6727	5.06E-01	20901	1.6571	0.4224	8.75E-05	52871
						DBP	1.2762	0.2552	5.73E-07	31967	1.7709	3.7499	6.37E-01	20901	1.2785	0.2546	5.13E-07	52868
						PP	0.3841	0.2996	2.00E-01	31967	2.7247	4.3212	5.28E-01	20901	0.3953	0.2989	1.86E-01	52868
						HTN	0.1069	0.0505	3.42E-02	27225.6	-0.2018	0.9575	8.33E-01	21170	0.106	0.0504	3.55E-02	48395.6
						CPASSOC (SHom)	NA	NA	2.88E-05	31970/31967/27225	NA	NA	8.99E-01	20901/20901/21170	NA	NA	1.22E-03	52871/52868/48396
						CPASSOC (SHet)	NA	NA	4.94E-06	31970/31967/27225	NA	NA	7.72E-01	20901/20901/21170	NA	NA	2.01E-04	52871/52868/48396
rs71512425	9	28165694	a	g	0.80	SBP	0.8122	0.225	3.07E-04	31970	0.0033	0.1894	9.86E-01	35538	0.3389	0.1449	1.94E-02	67508
						DBP	0.6974	0.1348	2.32E-07	31967	-0.0322	0.1102	7.70E-01	38168	0.2599	0.0853	2.31E-03	70135
						PP	0.124	0.1605	4.40E-01	31967	-0.0253	0.1422	8.59E-01	35538	0.0403	0.1064	7.05E-01	67505
						HTN	0.0456	0.0262	8.12E-02	27878.6	0.0318	0.0252	2.06E-01	35798	0.0384	0.0182	3.42E-02	63676.6
						CPASSOC (SHom)	NA	NA	6.47E-05	31970/31967/27879	NA	NA	6.75E-01	35538/38168/35798	NA	NA	1.42E-03	67508/70135/63677
						CPASSOC (SHet)	NA	NA	1.99E-06	31970/31967/27879	NA	NA	3.84E-01	35538/38168/35798	NA	NA	3.27E-05	67508/70135/63677
**rs115795127[b,c]**	9	85993901	t	c	0.89	SBP	0.9906	0.2847	5.03E-04	31970	8.7717	9.0475	3.32E-01	4473	0.9983	0.2846	4.51E-04	36443
						DBP	0.4763	0.1716	5.52E-03	31967	0.7026	6.6522	9.16E-01	4473	0.4765	0.1715	5.48E-03	36440
						PP	0.5871	0.2022	3.69E-03	31967	9.2572	8.6147	2.83E-01	4473	0.5919	0.2021	3.41E-03	36440
						HTN	0.172	0.0325	1.23E-07	27675.5	**4.4894**	**1.6767**	**7.42E-03**	**4473**	**0.1736**	**0.0325**	**1.13E-08**	**32148.5**
						CPASSOC (SHom)	NA	NA	5.10E-06	31970/31967/27675	NA	NA	1.07E-01	4473/4473/4473	NA	NA	8.42E-06	36443/36440/32149
						CPASSOC (SHet)	NA	NA	1.05E-06	31970/31967/27675	**NA**	**NA**	**8.41E-06**	**4473/4473/4473**	**NA**	**NA**	**7.30E-09**	**36443/36440/32149**
rs190531342	9	114719568	t	c	0.92	SBP	-1.5414	0.4156	2.09E-04	30356	3.5014	1.4213	1.38E-02	28014	-1.1442	0.3989	4.13E-03	58370
						DBP	-1.0192	0.2484	4.09E-05	30353	1.8065	0.8716	3.82E-02	28014	-0.807	0.2389	7.30E-04	58367
						PP	-0.5053	0.2939	8.55E-02	31967	1.6605	1.151	1.49E-01	28014	-0.3727	0.2848	1.91E-01	59981
						HTN	-0.2325	0.0495	2.65E-06	25877.2	0.2763	0.171	1.06E-01	28286	-0.1932	0.0475	4.86E-05	54163.2
						CPASSOC (SHom)	NA	NA	9.63E-07	30356/30353/25877	NA	NA	6.07E-02	28014/28014/28286	NA	NA	1.99E-06	58370/58367/54163
						CPASSOC (SHet)	NA	NA	5.19E-06	30356/30353/25877	NA	NA	2.10E-01	28014/28014/28286	NA	NA	3.61E-05	58370/58367/54163
rs10123054	9	128452054	t	c	0.65	SBP	-0.6921	0.182	1.43E-04	31003	-0.2167	0.1298	9.51E-02	37754	-0.377	0.1057	3.61E-04	68757
						DBP	-0.5706	0.11	2.13E-07	31000	-0.0705	0.0798	3.77E-01	37754	-0.2428	0.0646	1.70E-04	68754
						PP	-0.0933	0.1302	4.74E-01	31000	-0.159	0.0927	8.61E-02	37754	-0.1369	0.0755	6.97E-02	68754
						HTN	-0.0667	0.021	1.48E-03	27582.1	-0.0131	0.0173	4.50E-01	38026	-0.0348	0.0134	9.22E-03	65608.1
						CPASSOC (SHom)	NA	NA	2.70E-06	31003/31000/27582	NA	NA	3.23E-01	37754/37754/38026	NA	NA	3.42E-05	68757/68754/65608
						CPASSOC (SHet)	NA	NA	1.85E-06	31003/31000/27582	NA	NA	6.78E-01	37754/37754/38026	NA	NA	6.89E-05	68757/68754/65608
rs28687694	9	128483092	t	c	0.26	SBP	0.8674	0.2036	2.05E-05	28472	-0.146	0.142	3.04E-01	37494	0.1856	0.1165	1.11E-01	65966
						DBP	0.5658	0.1233	4.43E-06	28469	-0.1233	0.0877	1.60E-01	37494	0.1081	0.0715	1.30E-01	65963
						PP	0.296	0.1461	4.28E-02	30083	0.026	0.0996	7.94E-01	37494	0.1117	0.0823	1.75E-01	67577
						HTN	0.0967	0.0238	4.75E-05	24483.4	-0.0157	0.0187	4.01E-01	37763	0.0271	0.0147	6.48E-02	62246.4
						CPASSOC (SHom)	NA	NA	5.85E-07	28472/28469/24483	NA	NA	3.43E-01	37494/37494/37763	NA	NA	9.59E-06	65966/65963/62246
						CPASSOC (SHet)	NA	NA	5.06E-06	28472/28469/24483	NA	NA	6.22E-01	37494/37494/37763	NA	NA	1.44E-04	65966/65963/62246
rs78687626	9	133280171	a	g	0.01	SBP	-6.1309	1.7746	5.51E-04	14481	-0.4687	0.6024	4.37E-01	26512	-1.0537	0.5704	6.47E-02	40993
						DBP	-1.362	1.0386	1.90E-01	14478	-0.1279	0.3553	7.19E-01	26512	-0.2572	0.3362	4.44E-01	40990
						PP	-7.3536	1.4531	4.18E-07	12013	-0.2388	0.4874	6.24E-01	26512	-0.9582	0.4621	3.81E-02	38525
						HTN	-0.5341	0.1962	6.48E-03	13196	0.0703	0.0685	3.05E-01	26512	0.0046	0.0647	9.43E-01	39708
						CPASSOC (SHom)	NA	NA	4.33E-03	14481/14478/13196	NA	NA	9.84E-01	26512/26512/26512	NA	NA	8.06E-02	40993/40990/39708
						CPASSOC (SHet)	NA	NA	3.42E-03	14481/14478/13196	NA	NA	2.13E-01	26512/26512/26512	NA	NA	5.99E-03	40993/40990/39708
10:4699372:GTGTT	10	4699372	g	gtgtt	0.08	SBP	-1.5547	0.4072	1.34E-04	28554	-1.6356	5.1122	7.49E-01	10729	-1.5552	0.4059	1.27E-04	39283
						DBP	-1.2281	0.2402	3.19E-07	28551	-3.0155	3.3472	3.68E-01	10729	-1.2373	0.2396	2.42E-07	39280
						PP	-0.2925	0.2981	3.27E-01	26937	0.1823	4.0482	9.64E-01	10729	-0.2899	0.2973	3.29E-01	37666
						HTN	-0.1231	0.0508	1.54E-02	20645.3	-0.8704	1.009	3.88E-01	10729	-0.125	0.0507	1.38E-02	31374.3
						CPASSOC (SHom)	NA	NA	1.43E-05	28554/28551/20645	NA	NA	3.96E-01	10729/10729/10729	NA	NA	7.41E-05	39283/39280/31374
						CPASSOC (SHet)	NA	NA	2.75E-06	28554/28551/20645	NA	NA	7.52E-01	10729/10729/10729	NA	NA	2.91E-05	39283/39280/31374
10:14646769:T_TCA	10	14646769	t	tca	0.59	SBP	-0.9063	0.2187	3.43E-05	26670.9	-0.0948	0.1531	5.36E-01	31011	-0.3618	0.1254	3.93E-03	57681.9
						DBP	-0.1799	0.1292	1.64E-01	26667.9	0.1292	0.0911	1.56E-01	31011	0.0265	0.0745	7.22E-01	57678.9
						PP	-0.7905	0.1584	6.01E-07	25053.9	-0.168	0.1071	1.17E-01	31011	-0.3633	0.0887	4.22E-05	56064.9
						HTN	-0.0397	0.0265	1.35E-01	20184	0.0287	0.0248	2.47E-01	19441	-0.0032	0.0181	8.58E-01	39625
						CPASSOC (SHom)	NA	NA	9.92E-03	26671/26668/20184	NA	NA	3.78E-01	31011/31011/19441	NA	NA	2.47E-02	57682/57679/39625
						CPASSOC (SHet)	NA	NA	2.77E-04	26671/26668/20184	NA	NA	5.12E-04	31011/31011/19441	NA	NA	2.38E-06	57682/57679/39625
rs77932157	10	69457644	a	g	0.19	SBP	-0.2091	0.225	3.53E-01	31970	-0.2711	0.4968	5.85E-01	23914	-0.2197	0.205	2.84E-01	55884
						DBP	0.3957	0.1353	3.46E-03	31967	-0.1187	0.298	6.90E-01	23914	0.3078	0.1232	1.25E-02	55881
						PP	-0.5841	0.16	2.62E-04	31967	-0.3265	0.3864	3.98E-01	23914	-0.5464	0.1478	2.19E-04	55881
						HTN	-0.0254	0.0261	3.31E-01	27846.5	-0.0996	0.0648	1.24E-01	23914	-0.0358	0.0242	1.40E-01	51760.5
						CPASSOC (SHom)	NA	NA	6.77E-01	31970/31967/27846	NA	NA	2.94E-01	23914/23914/23914	NA	NA	5.20E-01	55884/55881/51761
						CPASSOC (SHet)	NA	NA	4.61E-07	31970/31967/27846	NA	NA	4.27E-01	23914/23914/23914	NA	NA	3.24E-06	55884/55881/51761
10:69514659:AAAG_	10	69514659	a	aaag	0.09	SBP	-1.1361	0.3537	1.32E-03	28554	-0.4742	0.5137	3.56E-01	23914	-0.9232	0.2913	1.53E-03	52468
						DBP	0.171	0.2076	4.10E-01	28551	-0.1574	0.3086	6.10E-01	23914	0.0687	0.1723	6.90E-01	52465
						PP	-1.1622	0.2557	5.50E-06	26937	-0.5098	0.4006	2.03E-01	23914	-0.9733	0.2155	6.30E-06	50851
						HTN	-0.0793	0.0428	6.42E-02	21024.3	-0.1336	0.0672	4.68E-02	23914	-0.095	0.0361	8.52E-03	44938.3
						CPASSOC (SHom)	NA	NA	1.21E-01	28554/28551/21024	NA	NA	1.55E-01	23914/23914/23914	NA	NA	9.30E-02	52468/52465/44938
						CPASSOC (SHet)	NA	NA	5.40E-07	28554/28551/21024	NA	NA	2.01E-01	23914/23914/23914	NA	NA	1.85E-06	52468/52465/44938
rs11196093	10	114524327	c	g	0.34	SBP	-0.7212	0.182	7.40E-05	31969.9	0.1129	0.1886	5.49E-01	36992	-0.319	0.131	1.49E-02	68961.9
						DBP	-0.1127	0.1096	3.04E-01	31966.9	0.1234	0.1164	2.89E-01	36992	-0.0017	0.0798	9.83E-01	68958.9
						PP	-0.6431	0.1291	6.31E-07	31966.9	-0.0413	0.1323	7.55E-01	36992	-0.3495	0.0924	1.55E-04	68958.9
						HTN	-0.0491	0.021	1.93E-02	28131	0.045	0.0258	8.18E-02	33824	-0.0117	0.0163	4.73E-01	61955
						CPASSOC (SHom)	NA	NA	6.10E-03	31969/31969/28131	NA	NA	2.61E-01	36992/36992/33824	NA	NA	1.69E-02	68962/68959/61955
						CPASSOC (SHet)	NA	NA	5.86E-04	31969/31969/28131	NA	NA	5.73E-01	36992/36992/33824	NA	NA	6.76E-03	68962/68959/61955
rs7941684	11	5532222	t	g	0.80	SBP	-1.2323	0.2209	2.44E-08	31969.9	-0.0909	0.1483	5.40E-01	47955	-0.4455	0.1231	2.96E-04	79924.9
						DBP	-0.5388	0.1325	4.79E-05	31966.9	0.0151	0.0829	2.13E-01	47955	-0.2256	0.0703	1.32E-03	79921.9
						PP	-0.6802	0.1567	1.42E-05	31966.9	0.0536	0.0955	5.74E-01	47955	-0.145	0.0815	7.54E-02	79921.9
						HTN	-0.071	0.0253	5.03E-03	27872.2	-0.0081	0.0183	6.60E-01	48190	-0.0297	0.0148	4.52E-02	76062.2
						CPASSOC (SHom)	NA	NA	3.85E-06	31969/31969/27827	NA	NA	6.80E-01	47955/47955/48190	NA	NA	3.29E-04	79925/79922/76062
						CPASSOC (SHet)	NA	NA	5.82E-07	31969/31969/27827	NA	NA	4.71E-01	47955/47955/48190	NA	NA	3.58E-05	79925/79922/76062
rs145171075	11	80139405	t	g	0.98	SBP	4.4592	0.9098	9.52E-07	24262	-10.96	8.3346	1.89E-01	8735	4.2776	0.9044	2.25E-06	32997
						DBP	1.558	0.5227	2.88E-03	24259	-3.968	5.1286	4.39E-01	8735	1.5012	0.52	3.89E-03	32994
						PP	3.0231	0.6382	2.17E-06	24259	-6.992	5.164	1.76E-01	8735	2.8724	0.6334	5.76E-06	32994
						HTN	0.401	0.0997	5.81E-05	22010	-0.6397	0.9131	4.84E-01	8735	0.3887	0.0991	8.77E-05	30745
						CPASSOC (SHom)	NA	NA	5.16E-06	24262/24259/22010	NA	NA	3.01E-01	8735/8735/8735	NA	NA	2.23E-05	32997/32994/30745
						CPASSOC (SHet)	NA	NA	3.92E-06	24262/24259/22010	NA	NA	5.43E-01	8735/8735/8735	NA	NA	2.99E-05	32997/32994/30745
						SBP	-0.3334	0.1754	5.74E-02	30841	-0.0635	0.1296	6.24E-01	38701	-0.1588	0.1042	1.28E-01	69542
						DBP	-0.2203	0.1041	3.43E-02	30838	-0.1062	0.08	1.85E-01	38701	-0.1486	0.0634	1.92E-02	69539

rs10842715	12	26487183	t	g	0.46	PP	-0.1279	0.1228	2.98E-01	30838	-0.0402	0.0926	6.64E-01	38701	-0.072	0.0739	3.30E-01	69539
						HTN	-0.1026	0.0204	4.79E-07	26649	-0.0133	0.0171	4.37E-01	38971	-0.0502	0.0131	1.29E-04	65620
						CPASSOC (SHom)	NA	NA	2.50E-04	30841/30838/26649	NA	NA	7.30E-01	38701/38701/38971	NA	NA	9.63E-03	69542/69539/65620
						CPASSOC (SHet)	NA	NA	4.26E-06	30841/30838/26649	NA	NA	4.64E-01	38701/38701/38971	NA	NA	1.81E-04	69542/69539/65620
12:53049050:AC_A	12	53049050	a	ac	0.86	SBP	-1.7032	0.4322	8.11E-05	17426	0.6576	0.2759	1.72E-02	28247	-0.0259	0.2326	9.11E-01	45673
						DBP	-1.1805	0.2708	1.30E-05	17426	0.0525	0.1626	7.47E-01	28247	-0.2741	0.1394	4.92E-02	45673
						PP	-0.46	0.3141	1.43E-01	15812	0.6155	0.1932	1.45E-03	28247	0.3202	0.1646	5.17E-02	44059
						HTN	-0.2433	0.0554	1.15E-05	11700	-0.0151	0.0431	7.26E-01	19441	-0.1011	0.034	2.95E-03	31141
						CPASSOC (SHom)	NA	NA	7.67E-07	17426/17426/11700	NA	NA	9.72E-01	28247/28247/19441	NA	NA	1.13E-05	45673/45673/31141
						CPASSOC (SHet)	NA	NA	5.49E-06	17426/17426/11700	NA	NA	1.21E-01	28247/28247/19441	NA	NA	1.01E-05	45673/45673/31141
**rs113866309[b,c]**	12	66516948	t	c	0.98	SBP	-3.7312	0.8675	1.70E-05	23760	-12.0824	10.7806	2.60E-01	10729	-3.7821	0.8633	1.24E-05	34489
						DBP	-0.6665	0.5167	1.97E-01	23757	8.3116	7.3912	2.63E-01	10729	-0.2559	0.4539	2.33E-01	34486
						PP	-3.2809	0.6275	1.71E-07	23757	**-9.7833**	**3.3063**	**1.50E-03**	**9684**	**-2.2172**	**0.5424**	**8.24E-09**	**33441**
						HTN	-0.2244	0.1033	2.99E-02	22043	-3.5217	3.3044	2.83E-01	10729	-0.1475	0.0943	2.74E-02	32772
						CPASSOC (SHom)	NA	NA	3.91E-03	23760/23757/22043	NA	NA	3.50E-01	10729/10729/10729	NA	NA	1.87E-02	34489/34486/32772
						CPASSOC (SHet)	NA	NA	1.40E-04	23760/23757/22043	NA	NA	1.60E-02	10729/10729/10729	NA	NA	4.78E-05	34489/34486/32772
rs144058433	13	73013077	t	c	0.14	SBP	1.1947	0.2826	2.37E-05	31969.9	-0.1628	0.255	5.23E-01	36119	0.4465	0.1893	1.84E-02	68088.9
						DBP	0.8518	0.1695	5.03E-07	31966.9	0.023	0.1622	8.87E-01	36119	0.4192	0.1172	3.48E-04	68085.9
						PP	0.3197	0.2002	1.10E-01	31966.9	-0.1381	0.1718	4.21E-01	36119	0.0561	0.1304	6.67E-01	68085.9
						HTN	0.1119	0.0327	6.29E-04	27732.3	-0.0032	0.0337	9.24E-01	36364	0.056	0.0235	1.69E-02	64096.3
						CPASSOC (SHom)	NA	NA	1.08E-06	31969/31966/27732	NA	NA	5.35E-01	36119/36119/36364	NA	NA	3.00E-05	68089/68086/64096
						CPASSOC (SHet)	NA	NA	4.35E-06	31969/31966/27732	NA	NA	9.11E-01	36119/36119/36364	NA	NA	2.55E-04	68089/68086/64096
rs2414856	15	65072461	a	g	0.51	SBP	0.8733	0.1786	1.02E-06	30713.9	0.3052	0.2249	1.75E-01	38481	0.6535	0.1399	2.97E-06	69194.9
						DBP	0.2552	0.1081	1.83E-02	30710.9	0.1833	0.1384	1.85E-01	38481	0.228	0.0852	7.46E-03	69191.9
						PP	0.6783	0.127	9.18E-08	30710.9	0.1527	0.17	3.69E-01	38481	0.49	0.1017	1.46E-06	69191.9
						HTN	0.0686	0.021	1.10E-03	26574.6	0.0256	0.0301	3.95E-01	38754	0.0545	0.0172	1.55E-03	65328.6
						CPASSOC (SHom)	NA	NA	6.89E-05	30713/30710/26574	NA	NA	5.06E-01	38481/38481/38754	NA	NA	1.91E-03	69195/69192/65329
						CPASSOC (SHet)	NA	NA	8.70E-06	30713/30710/26574	NA	NA	6.72E-01	38481/38481/38754	NA	NA	6.17E-04	69195/69192/65329
rs12445099	16	57890233	a	g	0.67	SBP	0.6013	0.1944	1.98E-03	31970	0.2477	0.2368	2.95E-01	35367	0.4589	0.1502	2.25E-03	67337
						DBP	0.2529	0.117	3.07E-02	31967	0.0723	0.1436	6.14E-01	35367	0.1808	0.0907	4.62E-02	67334
						PP	0.36	0.1373	8.74E-03	31967	0.2399	0.1813	1.86E-01	35367	0.3162	0.1095	3.86E-03	67334
						HTN	0.1193	0.023	2.16E-07	27573.2	-0.0317	0.0303	2.94E-01	35599	0.064	0.0183	4.74E-04	63172.2
						CPASSOC (SHom)	NA	NA	3.40E-05	31970/31967/27573	NA	NA	7.75E-01	35367/35367/35599	NA	NA	1.04E-03	67337/67334/63172
						CPASSOC (SHet)	NA	NA	1.86E-06	31970/31967/27573	NA	NA	1.89E-01	35367/35367/35599	NA	NA	1.29E-05	67337/67334/63172
rs12149202	16	85700360	a	g	0.11	SBP	-1.3354	0.3011	9.21E-06	31970	0.0767	0.1592	6.30E-01	36873	-0.2319	0.1408	9.94E-02	68843
						DBP	-0.6867	0.1813	1.53E-04	31967	0.0948	0.0976	3.32E-01	36873	-0.0809	0.086	3.47E-01	68840
						PP	-0.7034	0.2168	1.18E-03	31967	-0.0135	0.1142	9.06E-01	36873	-0.1633	0.101	1.06E-01	68840
						HTN	-0.1877	0.0345	5.14E-08	27643.5	-0.0224	0.0205	2.73E-01	37140	-0.0655	0.0176	2.01E-04	64783.5
						CPASSOC (SHom)	NA	NA	9.24E-08	31970/31967/27643	NA	NA	9.99E-01	36973/36873/37140	NA	NA	1.33E-05	68843/68840/64784
						CPASSOC (SHet)	NA	NA	2.98E-07	31970/31967/27643	NA	NA	4.49E-01	36973/36873/37140	NA	NA	7.52E-06	68843/68840/64784
rs17721557	17	27260017	t	c	0.21	SBP	1.0488	0.2099	5.82E-07	30086	0.0711	0.2085	7.33E-01	37483	0.5566	0.1479	1.68E-04	67569
						DBP	0.5784	0.1266	4.87E-06	30083	0.1056	0.1271	4.06E-01	37483	0.343	0.0897	1.32E-04	67566
						PP	0.5216	0.1497	4.93E-04	30083	-0.063	0.1532	6.81E-01	37483	0.236	0.1071	2.75E-02	67566
						HTN	0.0597	0.0245	1.47E-02	25498.2	0.0076	0.0273	7.81E-01	37756	0.0365	0.0182	4.55E-02	63254.2
						CPASSOC (SHom)	NA	NA	6.13E-06	30086/30083/25498	NA	NA	6.14E-01	37483/37483/37756	NA	NA	1.66E-04	67569/67566/63254
						CPASSOC (SHet)	NA	NA	2.22E-06	30086/30083/25498	NA	NA	8.58E-01	37483/37483/37756	NA	NA	1.24E-04	67569/67566/63254
rs114296860	17	51285996	t	c	0.02	SBP	2.5521	0.6484	8.29E-05	30321	-11.985	14.626	4.13E-01	9684	2.5236	0.6478	9.79E-05	40005
						DBP	1.6074	0.3904	3.84E-05	30318	-5.247	8.863	5.54E-01	9684	1.5941	0.39	4.37E-05	40002
						PP	1.1088	0.4557	1.50E-02	30318	-6.88	10.815	5.25E-01	9684	1.0946	0.4553	1.62E-02	40002
						HTN	-0.042	0.0772	5.86E-01	25456	-0.676	4.305	8.75E-01	9684	-0.0422	0.0772	5.85E-01	35140
						CPASSOC (SHom)	NA	NA	7.16E-03	30321/30318/25456	NA	NA	5.80E-01	9684/9684/9684	NA	NA	2.69E-02	40005/40002/35140
						CPASSOC (SHet)	NA	NA	2.65E-07	30321/30318/25456	NA	NA	8.47E-01	9684/9684/9684	NA	NA	3.66E-06	40005/40002/35140
rs2832976	21	32037484	t	c	0.16	SBP	-1.1975	0.2602	4.19E-06	31969.9	-0.0669	0.1387	6.30E-01	39617	-0.3172	0.1224	9.58E-03	71586.9
						DBP	-0.3333	0.1564	3.31E-02	31966.9	-0.0361	0.0862	6.75E-01	37007	-0.1054	0.0755	1.63E-01	68973.9
						PP	-0.9467	0.1865	3.86E-07	31966.9	0.038	0.0992	7.02E-01	39617	-0.179	0.0876	4.09E-02	71583.9
						HTN	-0.0719	0.0302	1.72E-02	27732.2	-0.0097	0.0179	5.89E-01	39832	-0.0259	0.0154	9.27E-02	67564.2
						CPASSOC (SHom)	NA	NA	6.57E-04	31969/31969/27732	NA	NA	5.47E-01	39617/37007/39832	NA	NA	6.97E-03	71587/68974/67564
						CPASSOC (SHet)	NA	NA	3.54E-05	31969/31969/27732	NA	NA	8.52E-01	39617/37007/39832	NA	NA	1.29E-03	71587/68974/67564
rs62225706	22	26680705	a	c	0.03	SBP	-3.9669	0.8283	1.68E-06	16221	-0.0782	0.4006	8.45E-01	26143	-0.8154	0.3607	2.38E-02	42364
						DBP	-2.624	0.4943	1.10E-07	15169	-0.028	0.2441	9.09E-01	26143	-0.537	0.2189	1.42E-02	41312
						PP	-1.6075	0.6099	8.40E-03	16218	0.1566	0.3286	6.34E-01	26143	-0.2403	0.2893	4.06E-01	42361
						HTN	-0.2607	0.0964	6.85E-03	12891	-0.0344	0.0464	4.58E-01	26387	-0.077	0.0418	6.55E-02	39278
						CPASSOC (SHom)	NA	NA	1.17E-06	16221/15169/12891	NA	NA	5.17E-01	26143/26143/26387	NA	NA	3.06E-05	42364/41312/39278
						CPASSOC (SHet)	NA	NA	4.62E-07	16221/15169/12891	NA	NA	7.73E-01	26143/26143/26387	NA	NA	2.57E-05	42364/41312/39278
rs6006767	22	45927045	t	c	0.94	SBP	-1.0401	0.3723	5.21E-03	31970	-0.3444	0.2025	8.90E-02	37910	-0.5033	0.1779	4.67E-03	69880
						DBP	-1.1597	0.2295	4.37E-07	30918	-0.2582	0.1245	3.81E-02	38497	-0.4632	0.1094	2.31E-05	69415
						PP	0.0491	0.2651	8.53E-01	31967	-0.1627	0.1418	2.51E-01	37910	-0.1156	0.125	3.55E-01	69877
						HTN	-0.0763	0.0455	9.35E-02	25742.1	-0.0151	0.0266	5.70E-01	38183	-0.0307	0.023	1.81E-01	63925.1
						CPASSOC (SHom)	NA	NA	2.63E-04	31970/30918/25742	NA	NA	2.63E-01	37910/38497/38183	NA	NA	1.36E-03	69880/69415/63925
						CPASSOC (SHet)	NA	NA	3.76E-06	31970/30918/25742	NA	NA	2.77E-01	37910/38497/38183	NA	NA	3.79E-05	69880/69415/63925

N: sample size for CPASSOC refers the sample sizes of SBP/DBP/HTN

[a] Variants identified reaching genome-wide significant threshold of 5.0 x 10^{-8} in discovery stage and successfully replicated with experimental significance in replication

[b] Variants identified not reaching genome-wide significant threshold of 5.0 x 10^{-8} in discovery stage but replicated with experimental significance in replication

[c] Novel blood pressure loci identified in current study

Bold statistics are the corresponding significant statistics for significants of identified variants
