# Supplementary material for: Single-trait and multi-trait genome-wide association analyses identify novel loci for blood pressure in African-ancestry populations
Source: PLoS Genet. 2017 May 12;13(5):e1006728. doi: 10.1371/journal.pgen.1006728 (PMC5446189; doi:10.1371/journal.pgen.1006728)
Supplement: S7 Table — (PDF) [file pgen.1006728.s012.pdf]

S7 Table. Summary of iHS signals in significant loci with frequency differences across ancestry populations

						Derived Allele Frequency				
**SNP**	**Chr**	**Pos**	**Gene**	**Derived Allele**	**Ancestry Allele**	**African American**	**African**	**European**	**Asian**	**iHS**
rs78192203	8	142375073	*GPR20*	A	T	0.2	0.21	0	0	-2.678
rs7006531	8	95110744	*CDH17*	G	T	0.15	0.19	0	0	-1.567
rs113866309	12	66516948	*LLPH*	C	T	0.02	0.02	0	0	-1.776
rs76987554	6	134080855	*TCF21*	T	C	0.09	0.09	0	0	-1.723
rs115795127	9	85993901	*FRMD3*	T	C	0.89	0.86	1	1	2.702
