# Supplementary material for: Single-trait and multi-trait genome-wide association analyses identify novel loci for blood pressure in African-ancestry populations
Source: PLoS Genet. 2017 May 12;13(5):e1006728. doi: 10.1371/journal.pgen.1006728 (PMC5446189; doi:10.1371/journal.pgen.1006728)
Supplement: S8 Table — (PDF) [file pgen.1006728.s013.pdf]

S8 Table. MAGENTA analysis

Rank	Database	Gene Set	Original number of genes	Effective number of genes	Nominal GSEA P value	FDR	Trait
1	BIOCARTA	GSK3_PATHWAY	27	26	0.0012	0.163	SBP
2	BIOCARTA	TH1TH2_PATHWAY	19	16	0.0016	0.157	DBP
3	BIOCARTA	SHH_PATHWAY	16	16	0.0021	0.156	SHOM
4	BIOCARTA	CSK_PATHWAY	24	19	0.0023	0.113	SHOM
5	BIOCARTA	EIF2_PATHWAY	11	10	0.0039	0.116	SHOM
6	BIOCARTA	CTLA4_PATHWAY	21	15	0.0043	0.228	DBP
7	BIOCARTA	VIP_PATHWAY	27	26	0.0044	0.137	SHOM
8	BIOCARTA	MEF2D_PATHWAY	21	18	0.0051	0.279	HTN
1	GOTERM	dendrite	111	107	3.00E-05	0.233	SBP
2	GOTERM	regulation of blood pressure	52	46	1.00E-04	0.200	PP
3	GOTERM	filopodium	25	24	1.00E-04	0.226	PP
4	GOTERM	response to nicotine	12	12	1.00E-04	0.273	SHOM
5	GOTERM	cellular defense response	60	49	3.00E-04	0.201	SHOM
6	GOTERM	nuclear chromosome telomeric region	15	15	4.00E-04	0.220	SHOM
7	GOTERM	recycling endosome membrane	18	15	4.00E-04	0.216	SHOM
8	GOTERM	membrane depolarization	16	15	5.00E-04	0.163	PP
9	GOTERM	histone deacetylation	17	15	6.00E-04	0.168	PP
10	GOTERM	high-density lipoprotein particle	19	15	7.00E-04	0.195	SHET
11	GOTERM	lipid transporter activity	19	18	7.00E-04	0.104	SHET
12	GOTERM	membrane fraction	479	437	9.00E-04	0.152	SHET
13	GOTERM	triglyceride metabolic process	24	23	0.001	0.196	SHET
14	GOTERM	activity	29	26	0.0013	0.224	PP
15	GOTERM	iron ion binding	65	58	0.0015	0.274	PP
16	GOTERM	negative regulation of gene-specific transcription from RNA polymerase II promoter	43	40	0.0017	0.280	PP
17	GOTERM	actin cytoskeleton organization	119	104	0.0018	0.277	PP
18	GOTERM	promoter binding	74	68	0.002	0.262	PP
19	GOTERM	phosphate metabolic process	21	21	0.002	0.202	PP
20	GOTERM	myeloid cell differentiation	15	15	0.0023	0.259	PP
21	GOTERM	Rho GTPase activator activity	22	18	0.0037	0.293	PP
22	GOTERM	DNA damage response signal transduction by p53 class mediator resulting in induction of apoptosis	13	13	0.0051	0.270	PP
23	GOTERM	sarcoplasmic reticulum	21	21	0.0053	0.297	PP
24	GOTERM	transcription repressor binding	12	11	0.0066	0.278	PP
25	GOTERM	polysaccharide binding	11	11	0.007	0.280	PP
26	GOTERM	methylation	12	11	0.0079	0.292	PP
27	GOTERM	heart development	123	120	0.0082	0.141	PP
1	Panther	Pyrimidine_Metabolism	6	6	0.004	0.137	DBP
2	Panther	Apoptosis_signaling_pathway	53	47	0.006	0.186	SBP
3	Panther	B_cell_activation	24	21	0.006	0.210	SBP
4	Panther	T_cell_activation	31	21	0.0064	0.263	SHOM
1	Ingenuity	JAK.Stat.Signaling	10	10	1.00E-04	0.011	DBP
2	Ingenuity	T.Cell.Receptor.Signaling	34	33	0.0021	0.160	SHOM
3	Ingenuity	B.Cell.Receptor.Signaling	35	33	0.0024	0.073	DBP
4	Ingenuity	Aryl.Hydrocarbon.Receptor.Signaling	52	50	0.0029	0.168	HTN
6	Ingenuity	Integrin.Signaling	38	37	0.0098	0.200	PP
1	KEGG	KEGG_CYTOKINE_CYTOKINE_RECEPTOR_INTERACTION	267	192	0.0013	0.263	DBP
2	KEGG	KEGG_VASCULAR_SMOOTH_MUSCLE_CONTRACTION	115	104	0.0015	0.256	PP
3	KEGG	KEGG_CARDIAC_MUSCLE_CONTRACTION	80	66	0.0018	0.278	PP
4	KEGG	KEGG_BASAL_CELL_CARCINOMA	55	53	0.0023	0.180	SHOM

6	KEGG	KEGG_STARCH_AND_SUCROSE_METABOLISM	52	37	0.0044	0.247	DBP
7	KEGG	KEGG_GLYCEROLIPID_METABOLISM	49	43	0.0047	0.255	SHET
1	PANTHER_BIOLOGICAL_PROCESS	Other_neuronal_activity	136	120	0.001	0.2364	HTN
2	PANTHER_BIOLOGICAL_PROCESS	T-cell_mediated_immunity	138	103	0.0018	0.2359	PP
3	PANTHER_BIOLOGICAL_PROCESS	Tumor_suppressor	102	72	0.0023	0.1611333	PP
4	PANTHER_BIOLOGICAL_PROCESS	DNA_metabolism	32	30	0.0023	0.14215	HTN
5	PANTHER_BIOLOGICAL_PROCESS	Phagocytosis	39	36	0.0026	0.1248	HTN
6	PANTHER_BIOLOGICAL_PROCESS	DNA_repair	169	138	0.0031	0.15365	HTN
8	PANTHER_BIOLOGICAL_PROCESS	Transport	509	384	0.0042	0.18848	HTN
9	PANTHER_BIOLOGICAL_PROCESS	Fatty_acid_beta-oxidation	27	23	0.0044	0.2383	PP
12	PANTHER_BIOLOGICAL_PROCESS	Cytokinesis	115	82	0.0056	0.1951833	HTN
13	PANTHER_BIOLOGICAL_PROCESS	Protein_phosphorylation	660	556	0.0082	0.247925	HTN
1	PANTHER_MOLECULAR_FUNCTION	Non-motor_actin_binding_protein	165	136	5.00E-04	0.150	SHET
2	PANTHER_MOLECULAR_FUNCTION	Transcription_cofactor	168	137	0.002	0.254	SHOM
3	PANTHER_MOLECULAR_FUNCTION	Dehydrogenase	225	183	0.004	0.298	PP
