# Supplementary material for: Single-trait and multi-trait genome-wide association analyses identify novel loci for blood pressure in African-ancestry populations
Source: PLoS Genet. 2017 May 12;13(5):e1006728. doi: 10.1371/journal.pgen.1006728 (PMC5446189; doi:10.1371/journal.pgen.1006728)
Supplement: S9 Table — (PDF) [file pgen.1006728.s014.pdf]

S9 Table. eQTL analysis of significant SNPs in tissues

SNP	Proxy	Correlation R2	Gene Symbol	P-Value	Effect Size	Tissue
rs6969780	rs6969780	NA	HOTAIRM1	2.50E-14	0.54	Esophagus - Mucosa
rs6969780	rs6969780	NA	HOXA2	8.10E-13	-0.6	Artery - Tibial
rs6969780	rs6969780	NA	HOTAIRM1	6.30E-12	0.59	Esophagus - Muscularis
rs6969780	rs6969780	NA	HOTAIRM1	1.80E-10	0.33	Lung
rs6969780	rs6969780	NA	HOTAIRM1	5.00E-10	0.58	Artery - Tibial
rs6969780	rs6969780	NA	HOTAIRM1	1.30E-09	0.64	Skin - Sun Exposed (Lower leg)
rs6969780	rs6969780	NA	HOXA2	1.60E-09	-0.62	Nerve - Tibial
rs6969780	rs6969780	NA	HOTAIRM1	5.70E-09	0.66	Cells - Transformed fibroblasts
rs6969780	rs6969780	NA	HOTAIRM1	2.90E-08	0.48	Adipose - Subcutaneous
rs6969780	rs6969780	NA	HOXA2	3.10E-07	-0.49	Adipose - Subcutaneous
rs6969780	rs6969780	NA	HOTAIRM1	4.70E-07	0.46	Muscle - Skeletal
rs6969780	rs6969780	NA	HOXA5	7.50E-07	-0.5	Cells - Transformed fibroblasts
rs6969780	rs6969780	NA	HOTAIRM1	0.0000023	0.53	Skin - Not Sun Exposed (Suprapubic)
rs6969780	rs6969780	NA	HOTAIRM1	0.0000025	0.36	Stomach
rs6969780	rs6969780	NA	HOXA7	0.0000032	-0.4	Artery - Tibial
rs7651190	rs7651190	NA	ULK4	1.10E-44	0.99	Cells - Transformed fibroblasts
rs7651190	rs7651190	NA	ULK4	1.70E-31	0.87	Artery - Aorta
rs7651190	rs7651190	NA	ULK4	1.80E-27	0.68	Whole Blood
rs7651190	rs7651190	NA	ULK4	8.50E-27	0.74	Thyroid
rs7651190	rs7651190	NA	ULK4	8.20E-25	0.67	Nerve - Tibial
rs7651190	rs7651190	NA	ULK4	5.10E-24	0.94	Esophagus - Mucosa
rs7651190	rs7651190	NA	ULK4	2.40E-21	0.62	Artery - Tibial
rs7651190	rs7651190	NA	ULK4	3.00E-19	0.67	Muscle - Skeletal
rs7651190	rs7651190	NA	ULK4	3.10E-18	0.85	Cells - EBV-transformed lymphocytes
rs7651190	rs7651190	NA	ULK4	6.70E-18	0.64	Esophagus - Muscularis
rs7651190	rs7651190	NA	ULK4	2.30E-15	0.59	Skin - Sun Exposed (Lower leg)
rs7651190	rs7651190	NA	ULK4	3.80E-15	1.2	Brain - Cortex
rs7651190	rs7651190	NA	ULK4	7.10E-14	0.76	Stomach
rs7651190	rs7651190	NA	ULK4	1.30E-13	0.45	Adipose - Subcutaneous
rs7651190	rs7651190	NA	ULK4	9.70E-13	1.2	Pituitary
rs7651190	rs7651190	NA	ULK4	1.10E-12	1.2	Brain - Anterior cingulate cortex (BA24)
rs7651190	rs7651190	NA	ULK4	2.30E-12	1.1	Brain - Frontal Cortex (BA9)
rs7651190	rs7651190	NA	ULK4	1.10E-11	0.88	Adrenal Gland
rs7651190	rs7651190	NA	ULK4	1.10E-11	0.64	Adipose - Visceral (Omentum)
rs7651190	rs7651190	NA	ULK4	1.80E-11	0.59	Colon - Transverse
rs7651190	rs7651190	NA	ULK4	6.50E-11	0.44	Lung
rs7651190	rs7651190	NA	ULK4	6.90E-11	1.1	Brain - Putamen (basal ganglia)
rs7651190	rs7651190	NA	ULK4	1.20E-10	0.89	Brain - Nucleus accumbens (basal ganglia)
rs7651190	rs7651190	NA	ULK4	1.30E-10	0.69	Pancreas
rs7651190	rs7651190	NA	RPL36P20	2.30E-09	0.55	Testis
rs7651190	rs7651190	NA	ULK4	1.20E-08	0.71	Colon - Sigmoid
rs7651190	rs7651190	NA	ULK4	2.20E-08	0.8	Spleen
rs7651190	rs7651190	NA	ULK4	2.90E-08	0.73	Brain - Caudate (basal ganglia)
rs7651190	rs7651190	NA	ULK4	8.60E-08	0.63	Brain - Hypothalamus
rs7651190	rs7651190	NA	ULK4	1.20E-07	0.63	Heart - Left Ventricle
rs7651190	rs7651190	NA	ULK4	1.30E-07	0.49	Skin - Not Sun Exposed (Suprapubic)
rs7651190	rs7651190	NA	ULK4	3.70E-07	0.55	Breast - Mammary Tissue
rs7651190	rs7651190	NA	ULK4	4.20E-07	0.91	Brain - Cerebellar Hemisphere
rs7651190	rs7651190	NA	ULK4	0.0000014	0.79	Brain - Cerebellum
rs7651190	rs7651190	NA	ULK4	0.0000031	0.57	Artery - Coronary
rs7651190	rs7651190	NA	ULK4	0.0000032	0.62	Esophagus - Gastroesophageal Junction
rs62434120	rs4869927	0.9	PLEKHG1	0.0000068	-0.27	Testis
rs62434120	rs9480528	0.86	PLEKHG1	0.0000068	-0.27	Testis
