# Supplementary material for: Single-trait and multi-trait genome-wide association analyses identify novel loci for blood pressure in African-ancestry populations
Source: PLoS Genet. 2017 May 12;13(5):e1006728. doi: 10.1371/journal.pgen.1006728 (PMC5446189; doi:10.1371/journal.pgen.1006728)
Supplement: S10 Table — (PDF) [file pgen.1006728.s015.pdf]

S10 Table. Primers of candidate genes.

The primers of candidate genes for real-time polymerase chain reaction (RT-PCR) primers were designed based on the latest mouse genome (GRCm38/mm10) using Primer3 (http://bioinfo.ut.ee/primer3-0.4.0/) online. Primers were also designed for isoforms of certain candidate genes.

Mouse Genes	Forward Primer	Reverse Primer
Ulk4	TCTTGGAAAGCCTCAAGAACA	AAAGGATGGTGTGGGATCTG
Eya4	GCTTTGAGCGAATAATGCAA	GTGCTTGATGTAGAGCCAAGAG
Tcf21	CTCCAAGCTGGACACTCTCA	TCACCACTTCCTTCAGGTCA
Evx1	CTTTACCCGGGAGCAGATT	GCTGACGCTTGTCCTTCAT
Hoxa1-1	GCAGACCTTTGACTGGATGA	GCGCTCGTGTAAGGTACTTG
Hoxa1-2	CCCAGACGGCTACTTACCAG	GGAGAAGACGTCTCTGAAGCA
Hoxa5	GCGCAAGCTGCACATTAG	GGCATGAGCTATTTCGATCC
Hoxa7	AAGCCAGTTTCCGCATCTAC	GCTCTTTCTTCCACTTCATGC
Hoxa9	CCACGCTTGACACTCACACT	AGCGAGCATGTAGCCAGTT
Hoxa10-1	TCCAGCCCCTTCAGAAAACA	GCTACGGCTGATCTCTAGGC
Hoxa10-2	TCAAGGCAGTTCCAAAGG	TCACTTGTCTGTCCGTGAGG
Hoxa11	GTCTTCCGGCCACACTGA	CAGTTGCAGACGCTTCTCTTT
Igfbp3	CGCAGAGAAATGGAGGACA	ACTTGTCCACACACCAGCAG
Cdh17	CCAGTTACTTTCTGCCAGTGTG	CCAGTTACTTTCTGCCAGTGTG
Gpr20	GCGTGGAGAAGAATTCAAGC	TCCTAGAGCCTTGACCTTTGA
Plekhg1	GTCAGCATAGGCCCAGTCA	CAGCCATCCTTCTGAGCTTT
Frmd3-1	TCAGACACCAGAGTTTGAGCA	TCTTGACAACTGAAGGCCAAT
Frmd3-2	AATCCTGACCGGCCATATC	GGATGTGTCCTCCATGTGC
Llph	GAGATAGCAACCGTGGTGGT	TCATCCACACTGGGTACTGG
Tmbim4	TCTGGTTCTGCAAGCGTTTA	ACCAGCTCCATCGTCTCACT
Hprt	CAAACTTTGCTTTCCCTGGT	CAAGGGCATATCCAACAACA
