# Supplementary material for: Single-trait and multi-trait genome-wide association analyses identify novel loci for blood pressure in African-ancestry populations
Source: PLoS Genet. 2017 May 12;13(5):e1006728. doi: 10.1371/journal.pgen.1006728 (PMC5446189; doi:10.1371/journal.pgen.1006728)
Supplement: S1 Note — (DOCX) [file pgen.1006728.s016.docx]

Single-trait and multi-trait genome wide association analyses identify novel loci for blood pressure in African-ancestry populations

**Supplementary Notes**

**AUTHOR CONTRIBUTIONS**

***Principal investigators (alphabetically for study names)***

***Manuscript writing***

***Phenotyping***

***Genotyping***

***Quality control***

***Software development***

***Statistical analysis***

***GWAS Look-ups in other Consortia***

**DESCRIPTION OF STUDY SAMPLES**

1. **Discovery COGENT BP studies**

***CARe***

**Candidate Gene Association Resource**  CARe samples were collected from five NHLBI-funded cohort studies where GWAS African American samples were available. (<http://public.nhlbi.nih.gov/GeneticsGenomics/home/care.aspx>)

***ARIC***

**Atherosclerosis Risk Communities Study**  The ARIC study is a population-based, biracial prospective cohort study of cardiovascular disease and its risk factors sponsored by National Heart, Lung and Blood Institute (NHLBI)[^1^](#_ENREF_1). ARIC included 15,792 European ancestry and African American individuals aged 45-64 years at baseline (1987-89), chosen by probability sampling from four US communities. Cohort members completed four clinic examinations, conducted three years apart between 1987 and 1998. Follow-up for clinical events was annual. The current analysis included only African American individuals with BP measures at baseline examinations. The IRB at each of the study sites approved the study protocols, and written informed consent was obtained from all participants.

BP was measured using a standardized Hawksley random-zero mercury column sphygmomanometer with participants in a sitting position after a resting period of 5 minutes. The size of the cuff was chosen according to the arm circumference. Three sequential recordings for SBP and DBP were obtained; the mean of the last two measurements was used in this analysis, discarding the first reading. BP lowering medication used was recorded from the medication history. Outliers (> 4 SDs from the mean) with respect to the SBP and DBP distribution were excluded from the analysis.

***CARDIA***

**The Coronary Artery Risk Development in Young Adults Study** The CARDIA study is a population based, prospective cohort examining the development and determinants of clinical and subclinical cardiovascular disease and its risk factors[^2^](#_ENREF_2). The CARDIA study initial enrollment consisted of 5,155 European Americans and African American men and women between 18 and 30 years old (52% African American and 55% women). The study is multicenter with recruitment in Birmingham, AL; Chicago, IL; Minneapolis, MN; and Oakland, CA. The IRB at each of the study sites approved the study protocols, and written informed consent was obtained from all participants. Baseline measurements were repeated, and additional measurements performed, at Years 2, 5, 7, 10, 15 and 20[^2^](#_ENREF_2). The current analysis included data measured at Year 15 (2000-2001) and only African American male and females.

Seated BP was measured on the right arm following 5 minutes rest using a random-zero sphygmomanometer. SBP and DBP were recorded as Phase I and Phase V Korotkoff sounds. Three measurements were taken at 1 minute intervals with the average of the second and third measurements taken for the BP values.

***CFS***

**The Cleveland Family Study** CFS participants consist of first or selected second-degree relatives of a proband with either laboratory diagnosed obstructive sleep apnea or neighborhood control of an affected proband. Families were selected for genotyping on the bases of genetic informativness, including multigenerational data or individuals from the extremes of the distribution of apnea phenotype[^3^](#_ENREF_3). These families include 59 African-American families with 176 individuals (100 females and 76 males) and 66 European-American families with 262 individuals (120 females and 142 males) with genotype and phenotype information. The IRB approved the study and written informed consent was obtained from all participants.

Participants had three supine BP measurements each performed after lying quietly for 10 minutes, before bed (10:00 PM) and upon awakening (7:00 AM), and another three sitting at 11:00 AM, following standardized guidelines using a calibrated sphygmomanometer. BP phenotypes were determined from the average of the nine measurements.

***JHS***

**Jackson Heart Study** JHS was initiated in 2000 to investigate prospectively the epidemiology and determinants of cardiovascular disease in African Americans[^4^](#_ENREF_4). JHS recruited 5,302 participants after completion of data adjustment, representing more than 5% of African American 35-84 years old living in the Jackson, Mississippi tri-county area. Of this number, ~30% were prior Jackson participants in the Atherosclerosis Risk in Communities Study. Of the remaining, 23% were recruited by random selection from a commercial listing that represents the overall tri-county population and an additional 23% volunteer sample, in which recruitment was distributed among defined demographic cells in proportions designed to mirror those in the overall population[^5^](#_ENREF_5). Those who were overlapping ARIC participants and those with previous MI were excluded from the GWAS. The IRB approved the study protocol, and written informed consent was obtained from all participants.

Seated BP was measured with a random-zero sphygmomanometer three times with the last two measurements averaged.

***MESA***

**The Multi-Ethnic Study of Atherosclerosis** The MESA is a multicenter prospective cohort study initiated to study the development of subclinical cardiovascular disease. A total of 6,814 women and men between the age of 45 and 84 year were recruited for the first examination between 2000 and 2002. Participants were recruited in six US cities (Baltimore, MD; Chicago, IL; Forsyth County, NC; Los Angele County, CA; Norther Manhattan, NY; and St. Paul, MN). Those with a history of CVD (defined as physician-diagnosed myocardial infarction, angina, heart failure, stroke, transient ischemic attack or history of invasive procedure for CVD) were excluded from participation. 38% are of European ancestry, 28% African-American, 22% Hispanic, and 12% Asian, predominantly of Chinese descent. This study was approved by the IRB of each study site, and written informed consent was obtained from all participants[^6^](#_ENREF_6). The manuscript utilizes data from African-American MESA participants, genotyped through the CARe project.

BP was measured three times at 1 minute interval after a 5 minute initial rest using a Dinamap PRO 100 automated oscillometric device (Critikon, Tampa, FL) with the subject in seated, and the average of the second and third BP measurements was used in the analysis.

***FBPP***

**Family Blood Pressure Program - AXIOM** These 872 African-American subjects were included from HyperGEN and GENOA studies but whom were not genotyped with conventional GWAS platforms. The sample schemes are the same as HyperGEN and GENOA. For BP measures, see HyperGEN and GENOA descriptions. These African-Americans were genotyped using Affymetrix Axiom chips, which included 808,558 SNPs. SNPs were called using Affymetrix Genotyping Console (GTC) by analyzing CEL files from Affymetrix AXIOM arrays ([www.affymetrix.com](http://www.affymetrix.com)).

***HANDLS***

**The Healthy Aging in Neighborhoods of Diversity across the Life Span Study** The Healthy Aging in Neighborhoods of Diversity across the Life Span study (HANDLS) is an interdisciplinary, community-based, prospective longitudinal epidemiologic study examining the influences of race and socioeconomic status (SES) on the development of age‐related health disparities among socioeconomically diverse African Americans and European ancestry individuals in Baltimore, Maryland, USA[^7^](#_ENREF_7). The HANDLS design is an area probability sample of Baltimore based on the 2000 Census. The study protocol facilitated our ability to recruit 3,720 participants from Baltimore. Among those who completed their examinations, there were no age differences associated with sex and poverty status, but African Americans were negligibly younger than individuals of European descent. The study is currently conducting Wave 4 designed as a second re-examination wave of all participants initially recruited at baseline (2004-2009). Wave 4 began in September of 2013 and will conclude in June of 2017. Genotyping was focused on a subset of participants self-reporting as African American was undertaken at the Laboratory of Neurogenetics, National Institute on Aging, National Institutes of Health. Genotype Imputation was performed using the 1000 Genomes Project phase 1 version 3 multi-ethnic reference panel, March 2012 release. BP was measured non-invasively using the brachial artery auscultation method with an aneroid manometer, a stethoscope, and an inflatable cuff in individuals resting for 5 minutes. For this analysis, the average of right and left sitting BP values was taken to represent each of SBP and DBP.

***CHS***

**Cardiovascular Health Study** The CHS is a population-based cohort study of risk factors for CHD and stroke in adults ≥65 years conducted across four field centers[^8^](#_ENREF_8). The original cohort, predominantly Americans of European Ancestry, comprised 5,201 persons who were recruited in 1989-1990 from random samples of the Medicare eligibility lists. Additional 687 individuals, predominantly African-Americans, were enrolled subsequently for a total sample of 5,888. DNA was extracted from blood samples drawn on all participants at their baseline examination in 1989-90 (original cohort) or 1992-93 (African American cohort). A sample of 823 African-Americans satisfying study design criteria, and with genome-wide association data, were used for analysis. Research staff with central training in BP measurement assessed repeated right-arm seated SBP and DBP levels at baseline with a Hawksley random-zero sphygmomanometer. The reported BP is the average of two measurements, which were taken after the participant had been sitting quietly for five minutes. First the technician determined the correct cuff size by measuring the arm circumference at the midpoint between the acromion and the olecranon. After applying the appropriate cuff, the maximum inflation level was determined by inflating the cuff until the radial pulse was no longer felt. The maximum inflation level was then determined to be the pulse obliteration pressure plus 30 mmHg plus the maximum zero level of the instrument. BP was measured by inflating the cuff to the maximum inflation level, waiting 5 seconds, then lowering by 2-3 mmHg per second. The first and fifth Korotkoff sounds were recorded. At least 30 seconds elapsed between each cuff inflation. Medication use was collected by interview. Information on prescription medication use in the previous two weeks was collected directly from the medications. A computer program developed by CHS was used to match the medication names with NDC numbers and then to group medications into analytic variables (e.g. beta blockers, lipid‐lowering medications)[^9^](#_ENREF_9). Means of the repeated BP measurements from the baseline examination were used for the analyses.

***GENOA***

**The Genetic Epidemiology Network of Arteriopathy (GENOA)**  GENOA is one of four networks in the Family Blood Pressure Program (FBPP) which recruited hypertensive African American and non‐Hispanic white sibships for linkage and family‐based association studies to investigate genetic contributions to BP in multiple racial groups[^10^](#_ENREF_10) . Recruitment (Exam 1, 1995-2000 and Exam 2, 2000-2005) was population-based in two geographic locations: Jackson, Mississippi and Rochester, Minnesota. African Americans were recruited solely at the Jackson field center. Hypertensive probands were ascertained from the Jackson cohort of the ARIC study if they were in a sibship with two individuals with essential hypertension (SBP =140 mmHg or DBP =90mmHg on the second and third clinic visit), diagnosed prior to age 60, and consented to participate. Index sib‐pairs with possible secondary hypertension, including sib‐pairs with previously diagnosed kidney disease (defined by serum creatinine level > 2 mg/dL), were excluded. After quality control procedures, and exclusion of all overlapping participants with ARIC, genotype data from a total of 996 African Americans was available for this study.

SBP and DBPs were measured using an automated oscillometric BP measurement device with a consistent protocol across the FBPP networks. BP was measured three times on each participant by trained and certified technicians and then averaged for use in this analysis.

***HRS***

**The Health and Retirement Study** The HRS is a longitudinal survey of a representative sample of Americans over age 50 sponsored by the National Institute on Aging (NIA) and conducted by the University of Michigan’s Institute for Social Research. The sample for this analysis includes 1,337 African Americans (N=483 males, 36.1%) interviewed in 2006 or 2008 with BP measured using an Omron HEM-780 Intellisence. Automated BP monitor with ComFit cuff. Participants that had missing values for both SBP and DBP, had missing values for covariates, and one individual that was > 5 SDs from the mean of BMI were excluded. Mean SBP and DBP from three measures. Genotyping was performed by the Center for Inherited Disease Research (CIDR) using Illumina’s Human Omni2.5-Quad BeadChip methodology. Genotyping quality control was performed by the Genetics Coordinating Center, Department of Biostatistics, University of Washington, Seattle.

***HyperGEN***

**The Hypertension Genetic Epidemiology Network**  HyperGEN is a multicenter family-based study to research the genetic causes of hypertension and related conditions[^11^](#_ENREF_11). HyperGEN recruited African American and Caucasian participants at five field centers, with recruitment based largely on ongoing population-based studies. Study participants were recruited as one of three main types of subjects: 1) as part of a hypertensive sibship with at least two siblings diagnosed with hypertension; 2) random subjects, who were age-matched with hypertensive sibs; or 3) unmedicated adult offspring of one or more of the hypertensive siblings. Subjects were brought into the clinic for a one day exam, and data were collected from questionnaires, a physical exam, and blood and urine samples. This study obtained informed consent from participants and approval from the appropriate IRBs. SBP and DBPs were measured using an automated oscillometric BP measurement device with a consistent protocol across the FBPP networks. BP was measured three times on each participant by trained and certified technicians and then averaged for use in this analysis.

***Maywood-Loyola Study***  Participants were self-identified African Americans from a working class suburb of Chicago, Illinois, USA who were enrolled in studies of BP at the Loyola University Medical Center in Maywood, Illinois, USA as part of the International Collaborative Study on Hypertension in Blacks (ICSHIB) which is described in detail elsewhere[^12^](#_ENREF_12). Briefly, nuclear families were identified through middle-aged probands who were not ascertained based on any phenotype. Thereafter all available first-degree relatives 18 years old and above were enrolled into the study cohort of families. A screening exam was completed by trained and certified research staff using a standardized protocol[^12^](#_ENREF_12)^,^[^13^](#_ENREF_13). Information was obtained on medical history, age, body weight and height. Protocols were reviewed and approved by the IRB at the Loyola University Chicago Stritch School of Medicine prior to recruitment activities. This present study included unrelated adults sampled and for whom information on anthropometrics, BP and use of antihypertensive medication was available. BP measurements were obtained using an oscillometric device, previously evaluated in our field settings[^13^](#_ENREF_13). Three measurements were taken three minutes apart and the average of the final two was used in the analysis. Individuals with SBP ≥140 mmHg, DBP ≥90 mmHg or on anti-hypertensive medication at time of exam were defined as hypertensive. Participants with hypertension were offered treatment after detection at the screening exam.

***Maywood-Nigeria Cohort-1 & Cohort-2***  The sampling frame for the Nigeria cohort was also provided by the International Collaborative Study on Hypertension in Blacks (ICSHIB) as described in detail elsewhere[^12^](#_ENREF_12). Study participants were recruited from Igbo-Ora and Ibadan in southwest Nigeria as part of a long-term study on the environmental and genetic factors underlying hypertension. The base cohort consists of over 15,000 participants with information available on anthropometrics, BP and use of antihypertensive medication. BP measurements followed the same protocol described in the Loyola-Maywood study. This present study included unrelated adults samples from the cohort and some hypertensive participants who were recruited as controls in the Africa-America Diabetes Mellitus (AADM) Study recruited from Ibadan in similar neighborhoods[^14^](#_ENREF_14). Both projects were reviewed and approved by the sponsoring US institutions (Loyola University Chicago and Howard University) and the University of Ibadan. All participants signed informed consent administered in either English or Yoruba. BP measurements were obtained using an oscillometric device, previously evaluated in our field settings[^13^](#_ENREF_13). Three measurements were taken three minutes apart and the average of the final two was used in the analysis. Individuals with SBP ≥140 mmHg, DBP ≥90 mmHg or on anti-hypertensive medication at time of exam were defined as hypertensive. Participants with hypertension were offered treatment after detection at the screening exam.

***HUFS***

**The Howard University Family Study** HUFS is population based family study of African Americans in the Washington metropolitan area. Investigators enrolled a randomly recruited set of families in addition to a set of unrelated individuals to study genetic and environmental factors of common complex diseases including hypertension. The IRB approved the study protocol, and written informed consent was obtained from all participants. A total of 1,192 unrelated individuals were included in this analysis.  Blood pressure (BP) was measured in the sitting position using an oscillometric device (Omron). Three BP readings were taken with a 10 minute interval between readings. The reported SBP and DBP readings were the average of the second and third readings.

***WHI***

**Women’s Health Initiative SNP Health Association Resource** WHI is a study of postmenopausal women (aged 50-79 years), comprising 161,808 women recruited from 40 U.S. clinical centers to participate in an observational study (WHI-OS) or in clinical trials (WHI-CT). Details of recruitment and follow-up are described elsewhere[^15^](#_ENREF_15)^,^[^16^](#_ENREF_16). BP was measured by certified staff using standardized procedures and instruments. Two BP measures were recorded after 5 minutes rest using a mercury sphygmomanometer. Appropriate cuff bladder size was determined at each visit based on arm circumference. Diastolic BP was taken from the phase V Korotkoff measures. The average of the two measurements, obtained 30 seconds apart, was used in analyses. Women were asked to bring all of their current prescription and over‐the‐counter medications to each visit. Demographic data, medical history and anthropometric measures were obtained at a baseline clinical visit.

The WHI SNP Health Association Resource (SHARe) minority cohort includes 8,515 self‐identified African American women from WHI who provided written informed consent for study participation and DNA analysis. WHI GARNET(Genome-wide Association Research Network into Effects of Treatment) is a nested case-control genetic study of gene-by-hormone therapy interaction on the risk of CVD events and incident diabetes, where CHD, stroke, venous thromboembolic (VTE) and diabetes cases were matched to controls based on age, race, hysterectomy, enrollment date, and length of follow-up. WHI_WHIMS+ (Women's Health Initiative Memory Study) is composed of hormone therapy trial participants aged ≥ 65 years at randomization and free of dementia at baseline. The Long Life Study is composed of 7,875 women aged 62 or older from the WHI Extension II, who participated in the Hormone Therapy Clinical Trials, and is focused on aging and health/disease conditions. Genotyping was performed using HumanOmniExpress Exome-8v1_B.

***GeneSTAR***

**Genetic Study of Atherosclerosis Risk** GeneSTAR is a 27 year prospective family-based study of incident CAD, diabetes, stroke, and other vascular diseases in initially healthy African American and European American adult relatives of probands with angiographically documented coronary disease prior to 60 years of age at the time of hospitalization for an acute CAD event in any of 10 Baltimore area hospitals[^17^](#_ENREF_17). The genotyped sample size is 3,200, with ~35 % African American (n=1,129).  Participants are siblings of the probands, offspring of the siblings and probands, and coparents of the offspring.  All participants were under 60 years of age at the time of enrollment (from 1983 to 2006). Demographic information, self‐reported medical history, medication use, and smoking information were obtained from a standardized interview[^18^](#_ENREF_18). BP was measured using a standard mercury sphygmomanometer, following the American Heart Association[^19^](#_ENREF_19) and JNC 6 guidelines[^20^](#_ENREF_20) . The mean of three resting BP readings, taken early morning, midday, and late afternoon during the screening day was used to characterize BP measurements. Hypertension was defined as the subject having a mean SBP of ≥140 mmHg, a mean DBP of ≥90 mmHg, and/or currently taking an antihypertensive medication.

***IPM***

**Mount Sinai IPM Biobank** The Bio*Me*Biobank is an ongoing, prospective, hospital- and outpatient- based population research program operated by The Charles Bronfman Institute for Personalized Medicine (IPM) at Mount Sinai and has enrolled over 33,000 participants since September 2007. Bio*Me* is an Electronic Medical Record (EMR)-linked biobank that integrates research data and clinical care information for consented patients at The Mount Sinai Medical Center, which serves diverse local communities of upper Manhattan with broad health disparities. Bio*Me* populations include 25% of African ancestry (AA), 36% of Hispanic Latino ancestry (HL), 30% of white European ancestry (EA), and 9% of other ancestry. The Bio*Me* disease burden is reflective of health disparities in the local communities. Bio*Me* operations are fully integrated in clinical care processes, including direct recruitment from clinical sites waiting areas and phlebotomy stations by dedicated recruiters independent of clinical care providers, prior to or following a clinician standard of care visit. Recruitment currently occurs at a broad spectrum of over 30 clinical care sites.  This present study included only unrelated, adult, self-reported African Americans.   Information on anthropometrics, demographics, BP and use of antihypertensive medication was derived from participants EMR. The Mount Sinai Biobank Project (IRB # 07-0529 0001 02 9 ME) operates under an IRB-approved research protocol with IRB-approved informed consent forms. All study participants provided written informed consent. The Mount Sinai IPM Biobank Program is supported by The Andrea and Charles Bronfman Philanthropies.

***BioVU***   BioVU is a DNA biorepository linked to a database of de-identified EMRs (electronic medical records), designed and implemented with the goal of supporting genetic association studies at Vanderbilt University, including the identification of factors that affect disease susceptibility, disease progression, and/or drug response. BioVU is an ongoing study with rapid accrual of DNA specimens, accumulating approximately 27,000 participants per year, and with a current size of over 200,000. The DNA samples were obtained from patients at the Vanderbilt University Hospital, including all clinics that are part of the hospital system. A detailed description of the human subjects protection applied to BioVU is described by Pulley *et al*[^21^](#_ENREF_21). The program is under continuous oversight by the IRB and was reviewed in detail by the federal Office for Human Research Protections (OHRP). Program planning for BioVU started in 2004, and sample accrual started in February 2007.Traits are constructed for BioVU using the Synthetic Derivative (SD) database. This database is only accessible to Vanderbilt investigators and available by IRB approval. The SD database is a research tool developed to enable studies with de-identified clinical data. The SD collection includes information extracted from the EMR systems, and indexed by the same one-way Research Unique Identifier (RUI) used to track samples. The SD contains 2.4 million total records, with highly detailed longitudinal clinical data for approximately one million subjects. The database incorporates data from multiple sources and includes diagnostic and procedure codes (ICD-9 and CPT), basic demographics (age, sex, race), text from clinical care including discharge summaries, nursing notes, progress notes, history and physical examination, problem lists and multi-disciplinary assessments, laboratory values, echocardiogram (ECG) diagnoses, imaging reports, electronically derived trace values, and inpatient medication orders. All clinical data are updated regularly to include new patients and append new data to clinical records of existing patients. BioVU uses discarded blood samples collected during routine patient care, linked to de-identified data extracted and continuously updated from the EMR.

For this blood pressure (BP) study, we used adult (age ≥ 18) BioVU participants with GWAS data. We used the first eligible outpatient measured BP in the EMR, and excluded measures at or after a diagnosis of secondary hypertension (ICD-9 405), chronic kidney or end-stage renal disease (ICD-9 group 585), thyroid disease (ICD-9 groups 240-246), diabetes (ICD-9 group 250), mental disorders (ICD-9 groups 290-319) or heart failure (ICD-9 group 428). We also excluded BP measures when they occurred at the same time as a diagnosis of atrial fibrillation (ICD-9 group 427), stroke (ICD-9 V17.1, 997.0, 992.0, V12.54), migraine (ICD-9 group 346), shock (ICD-9 group 785), myocardial infarction (ICD-9 group 410), poisoning (ICD-9 groups 960-989), and cancer (ICD-9 groups 140-239). We also censored measures taken within 1 year of death for any cause and measures taken in the inpatient clinical setting. To define hypertension cases,  participants’ measured systolic BP (SBP) or diastolic BP (DBP) ≥ 140mmHg or 90 mmHg respectively, have a diagnosis of hypertension (ICD-9 groups 401-404), or a prescription for antihypertensive medication prior to, or on the date, of BP measurement. Hypertension controls were defined by the absence of case criteria. BP measures were taken in outpatient clinics by sphygmomanometer for sitting patients.

1. **Replication multi-ethnic studies**

**East Asian Samples.** Three independent dataset from Korea were used for replication studies. They are called as KARE, HEXA and NC, respectively, and collected from population-based cohorts. The Korea Association Resource (KARE) cohort has 8,842 subjects with 352,228 SNPs genotyped by the Affymetrix Genome-Wide Human SNP Array 5.0. 3,703 subjects were recruited in Health Examinee (HEXA) cohort and genotyped for 646,062 SNPs with Affymetrix Genome-Wide Human SNP Array 6.0. Nong-Chon (NC) cohorts has collected 1,816 subjects who have 606,875 SNPs genotyped by Affymetrix Genome-Wide Human SNP Array 6.0.

In our imputation study for 72 variants, we excluded variants which the HWE p-values were less than 10^-5^, the minor allele frequencies (MAF) were less than 0.05 or the genotype missing rate were greater than 5% from the study panel. We also discarded subjects whose reported gender were discordant with sex chromosome, call rates were less than 95% or identity by state (IBS) was more than 0.8. We extracted only variants within +/- 500kb of 72 variants to construct study penal. Finally, we used 8,773 subjects with 3,865 SNPs for KARE, 3,702 subjects with 4,228 SNPs for HEXA and 1,814 subjects with 6,569 SNPs for NC as study panels, respectively. We utilized the 1000G Phase I Integrated Release Version 3 for reference panel.

By definition, there are 2,284 HTN patients in KARE, 665 HTN patients in HEXA and 858 HTN patients in NC. The mean and standard deviation (SD) of SBP, DBP and PP in KARE were 121.64 mmHg (18.62), 80.24 mmHg (11.46), and 41.41 mmHg (11.54), respectively. Similarly, HEXA showed 121.68 mmHg (14.37) of SBP, 80.24 mmHg (11.46) of DBP and 44.63 mmHg (9.26) of PP. Finally, the mean and SD of SBP, DBP and PP in NC were 133.81 mmHg (18.03), 83.88 mmHg (10.77) and 49.93 mm Hg (12.89), respectively.

**European American Samples**

**The Atherosclerosis Risk In Communities (ARIC).** The Atherosclerosis Risk In Communities Study is a population-based prospective cohort study of cardiovascular disease sponsored by the National Heart, Lung, and Blood Institute (NHLBI). ARIC included 15,792 individuals aged 45-64 years at baseline (1987-89), chosen by probability sampling from four US communities11. Cohort members completed four clinic examinations each spread over about three years, conducted approximately three years apart between 1987 and 1998. The data used in this study are from the first visit in 1987-1989. A detailed study protocol is available on the ARIC study website (<http://www.cscc.unc.edu/aric>). Blood pressure was measured using a standardized Hawksley random-zero mercury column sphygmomanometer with participants in a sitting position after a resting period of 5 minutes. The size of the cuff was chosen according to the arm circumference. Three sequential recordings for systolic and diastolic blood pressure were obtained; the mean of the last two measurements was used in this analysis, discarding the first reading. Blood pressure lowering medication use was recorded from the medication history. Outliers (>4 SD from the mean) with respect to the systolic or diastolic blood pressure distributions were excluded from the analysis. For this study the sample was restricted to individuals of European descent by self-report and principal component analysis using genome-wide genotypes.

**African Samples**

**Uganda Study** The Uganda study sought to determine whether a set of genetic loci significantly associated with blood pressure traits could be replicated among samples from the Medical Education Partnership Initiative for Cardiovascular Disease (MEPI-CVD) survey in Uganda, East Africa. The methods of the MEPI-CVD Survey have been described elsewhere [^22^](#_ENREF_22). In brief, MEPI-CVD Survey was a cross-sectional study conducted between September 2012 and May, 2013 in Wakiso district of central Uganda among men and women aged 18 years and older.  Data on CVD risk factors of interest was collected by trained research nurses using the World Health Organisation (WHO) modified expanded STEPs questionnaire. Subjects were asked to provide information on their age, sex, address, dietary habits, tobacco and alcohol consumption, exercise, smoke exposure, socio-economic status (housing characteristics), family history and symptoms of heart disease including angina. Self-reported history of hypertension diagnosis, diabetes, dyslipidaemia and the treatment for these conditions was also recorded. Anthropometric measurements collected included height, weight, and waist circumference. Blood pressure, fasting cholesterol and blood sugar measurements were also collected.

Participants were requested beforehand to refrain from smoking, drinking alcohol or caffeinated beverage a half an hour prior to blood pressure measurement. Blood pressure and heart rate was measured with an Omron automated sphygmomanometer model HEM-907. The BP was measured on the left arm after the participant had sat for at least five minutes. The blood pressure was taken in the sitting position, legs uncrossed, the arm resting on a table and the ante-cubital fossa at the level of the lower sternum. Two arm cuffs that fitted arm circumferences 9-13 inches and 13-17 inches were used in the process. Three readings were taken three minutes apart and the mean of the closest two values were used to describe the blood pressure of the subject. Additional measurements included height which was measured to the nearest 0.1 cm as the perpendicular distance between the top of the head (the vertex) and the bottom of the feet by a SECA 214 portable stadiometer. The weight was measured to the nearest 0.1 kg using a SECA 762 weighing scale with the subjects putting on loose clothing. The waist circumference was measured to the nearest 0.1 cm at the level of the midpoint between the inferior margin of the last rib and the crest of the ilium in the mid-axillary plane using a non-stretchable tape measure. Thirty SNPs were selected for genotyping and association analysis with BP. These SNPs were selected based on the previous association evidence with BP from GWASs or admixture mapping analysis[^23-28^](#_ENREF_23).

**ACKNOWLEDGEMENTS**

**BioVU:** The dataset(s) used for the analyses described were obtained from Vanderbilt University Medical Center’s BioVU which is supported by institutional funding and by the Vanderbilt CTSA grant 1UL1RR024975‐01 from NCRR/NIH. Support was also provided by Vanderbilt Clinical and Translational Research Scholar award (5KL2RR024975 to TLE), and Additional support was provided by the Building Interdisciplinary Research Careers in Women’s Health career development program (K12HD4383 to DRVE).

**CARe Acknowledgement**: The authors wish to acknowledge the support of the National Heart, Lung, and Blood Institute and the contributions of the research institutions, study investigators, field staff and study participants in creating this resource for biomedical research. The following nine parent studies have contributed parent study data, ancillary study data, and DNA samples through the Broad Institute (N01-HC-65226) to create this genotype/phenotype data base for wide dissemination to the biomedical research community. This work was also funded by the Center of Excellence in Personalized Medicine (CEPMED), the Canada Research Chair program, the “Fonds de recherche du Québec en Santé (FRQS)”, and the “Fondation de l’Institut de Cardiologie de Montréal” (to GL):

**Atherosclerotic Risk in Communities (ARIC):** University of North Carolina at Chapel Hill (N01HC-55015), Baylor Medical College (N01-HC-55016), University of Mississippi Medical Center (N01-HC-55021), University of Minnesota (N01-HC-55019), Johns Hopkins University (N01-HC55020), University of Texas, Houston (N01-HC-55017), University of North Carolina, Forsyth County (N01-HC-55018);

**Cardiovascular Health Study (CHS):** University of Washington (N01-HC-85079), Wake Forest University (N01-HC-85080), Johns Hopkins University (N01-HC-85081), University of Pittsburgh (N01-HC-85082), University of California, Davis (N01-HC-85083), University of California, Irvine (N01-HC-85084), New England Medical Center (N01-HC-85085), University of Vermont (N01HC-85086), Georgetown University (N01-HC-35129), Johns Hopkins University (N01 HC-15103), University of Wisconsin (N01-HC-75150), Geisinger Clinic (N01-HC-45133), University of Washington (N01 HC-55222, U01 HL080295); Cleveland Family Study (CFS): Case Western Reserve University (RO1 HL46380-01-16);

**Coronary Artery Risk in Young Adults (CARDIA):** University of Alabama at Birmingham (N01HC-48047), University of Minnesota (N01-HC-48048), Northwestern University (N01-HC-48049), Kaiser Foundation Research Institute (N01-HC-48050), University of Alabama at Birmingham (N01-HC-95095), Tufts-New England Medical Center (N01-HC-45204), Wake Forest University (N01-HC-45205), Harbor-UCLA Research and Education Institute (N01-HC-05187), University of California, Irvine (N01-HC-45134, N01-HC-95100);

**Multi‐Ethnic Study of Atherosclerosis (MESA):** MESA and the MESA SHARe project are conducted and supported by the National Heart, Lung, and Blood Institute (NHLBI) in collaboration with MESA investigators. Support for MESA is provided by contracts HHSN268201500003I, N01-HC-95159, N01-HC-95160, N01-HC-95161, N01-HC-95162, N01-HC-95163, N01-HC-95164, N01-HC-95165, N01-HC-95166, N01-HC-95167, N01-HC-95168, N01-HC-95169, UL1-TR-000040, UL1-TR-001079, UL1-TR-001420, UL1-TR-001881, and DK063491. The MESA CARe data used for the analyses described in this manuscript were obtained through Genetics (accession numbers).  Funding for CARe genotyping was provided by NHLBI Contract N01-HC-65226

**FBPP:** Axiom study is supported by the National Institutes of Health, grant number HL086718 from National Heart, Lung, Blood Institute. Z Zhang and X Zhu are supported by HL086718 from National Heart, Lung, Blood Institute and HG003054 from the National Human Genome Research Institute.

**GeneSTAR:** GeneSTAR was supported by NIH grants through the National Institute of Nursing Research (NR0224103) and the National Heart, Lung, and Blood Institute (HL58625-01A1, HL59684, HL071025-01A1, U01HL72518, and HL087698); and by M01-RR000052 to the Johns Hopkins General Clinical Research Center.

**GENOA:** Genetic Epidemiology Network of Arteriopathy (GENOA) study is supported by the National Institutes of Health, grant numbers HL087660 and HL100245 from the National Heart, Lung, Blood Institute. Ghana study: The authors wish to acknowledge the support of the study investigators, field staff and study participants in creating this resource for biomedical research. The Ghana Study is funded by NLM grant LM010098.

**HANDLS:** The Healthy Aging in Neighborhoods of Diversity across the Life Span Study (HANDLS) research was supported by the Intramural Research Program of the NIH, National Institute on Aging and the National Center on Minority Health and Health Disparities (project # Z01AG000513 and human subjects protocol # 2009‐149). Data analyses for the HANDLS study utilized the high‐performance computational capabilities of the Biowulf Linux cluster at the National Institutes of Health, Bethesda, Md. (http://biowulf.nih.gov).

**HUFS:** The Howard University Family Study was supported by National Institutes of Health grants S06GM008016‐320107 to Charles Rotimi and S06GM008016‐380111 to Adebowale Adeyemo. We thank the participants of the study, for which enrollment was carried out at the Howard University General Clinical Research Center, supported by National Institutes of Health grant 2M01RR010284. The contents of this publication are solely the responsibility of the authors and do not necessarily represent the official view of the National Institutes of Health. This research was supported in part by the Intramural Research Program of the Center for Research on Genomics and Global Health (CRGGH). The CRGGH is supported by the National Human Genome Research Institute, the National Institute of Diabetes and Digestive and Kidney Diseases, the Center for Information Technology, and the Office of the Director at the National Institutes of Health (Z01HG200362). Genotyping support was provided by the Coriell Institute for Medical Research.

**HyperGEN:** The hypertension network is funded by cooperative agreements (U10) with NHLBI: HL54471, HL54472, HL54473, HL54495, HL54496, HL54497, HL54509, HL54515, and 2 R01 HL55673‐12. The study involves: University of Utah (Network Coordinating Center, Field Center, and Molecular Genetics Lab); Univ. of Alabama at Birmingham (Field Center and Echo Coordinating and Analysis Center); Medical College of Wisconsin (Echo Genotyping Lab); Boston University (Field Center); University of Minnesota (Field Center and Biochemistry Lab); University of North Carolina (Field Center); Washington University (Data Coordinating Center); Weil Cornell Medical College (Echo Reading Center); National Heart, Lung, & Blood Institute. For a complete list of HyperGEN Investigators please see: www.biostat.wustl.edu/hypergen/Acknowledge.html

**Loyola-Nigeria:** The Loyola-Nigeria study was supported by NIH grant numbers R01-HL053353. The authors acknowledge the assistance of the research staff and participants in Ibadan and Igbo-Ora, Oyo State, Nigeria.

**Loyola-Maywood:** Maywood African-American study is supported by the National Institutes of Health grant number HL074166 from National Heart, Lung, Blood Institute.

**Mt Sinai IPM Study:** The Mt. Sinai IPM study was supported by The Andrea and Charles Bronfman Philanthropies. The authors would like to thank the participants from New York City, United States, for participating in the Mount Sinai IPM Biobank Program.

**WHI**: The WHI program is funded by the National Heart, Lung, and Blood Institute, National Institutes of Health, U.S. Department of Health and Human Services through contracts N01WH22110, 24152, 32100-2, 32105-6, 32108-9, 32111-13, 32115, 32118-32119, 32122, 42107-26, 42129-32, and 44221.

**HRS:** HRS is supported by the National Institute on Aging (NIA U01AG009740). The genotyping was funded separately by the National Institute on Aging (RC2 AG036495, RC4 AG039029). Genotyping was conducted by the NIH Center for Inherited Disease Research (CIDR) at Johns Hopkins University. Genotyping quality control and final preparation of the data were performed by the Genetics Coordinating Center at the University of Washington.

**SUPPLEMENTARY REFERENCES**

1. The Atherosclerosis Risk in Communities (ARIC) Study: design and objectives. The ARIC investigators. *Am J Epidemiol* **129**, 687-702 (1989).

2. Friedman, G.D. *et al.* CARDIA: study design, recruitment, and some characteristics of the examined subjects. *J Clin Epidemiol* **41**, 1105-16 (1988).

3. Palmer, L.J. *et al.* A whole-genome scan for obstructive sleep apnea and obesity. *Am J Hum Genet* **72**, 340-50 (2003).

4. Taylor, H.A., Jr. The Jackson Heart Study: an overview. *Ethn Dis* **15**, S6-1-3 (2005).

5. Fuqua, S.R. *et al.* Recruiting African-American research participation in the Jackson Heart Study: methods, response rates, and sample description. *Ethn Dis* **15**, S6-18-29 (2005).

6. Bild, D.E. *et al.* Multi-Ethnic Study of Atherosclerosis: objectives and design. *Am J Epidemiol* **156**, 871-81 (2002).

7. Evans, M.K. *et al.* Healthy aging in neighborhoods of diversity across the life span (HANDLS): overcoming barriers to implementing a longitudinal, epidemiologic, urban study of health, race, and socioeconomic status. *Ethn Dis* **20**, 267-75 (2010).

8. Fried, L.P. *et al.* The Cardiovascular Health Study: design and rationale. *Ann Epidemiol* **1**, 263-76 (1991).

9. Psaty, B.M. *et al.* Assessing the use of medications in the elderly: methods and initial experience in the Cardiovascular Health Study. The Cardiovascular Health Study Collaborative Research Group. *J Clin Epidemiol* **45**, 683-92 (1992).

10. Province, M.A. *et al.* A meta-analysis of genome-wide linkage scans for hypertension: the National Heart, Lung and Blood Institute Family Blood Pressure Program. *Am J Hypertens* **16**, 144-7 (2003).

11. Williams, R.R. *et al.* NHLBI family blood pressure program: methodology and recruitment in the HyperGEN network. Hypertension genetic epidemiology network. *Ann Epidemiol* **10**, 389-400 (2000).

12. Cooper, R. *et al.* The prevalence of hypertension in seven populations of west African origin. *Am J Public Health* **87**, 160-8 (1997).

13. Cooper, R. *et al.* Evaluation of an electronic blood pressure device for epidemiological studies. *Blood Press Monit* **2**, 35-40 (1997).

14. Rotimi, C.N. *et al.* A genome-wide search for type 2 diabetes susceptibility genes in West Africans: the Africa America Diabetes Mellitus (AADM) Study. *Diabetes* **53**, 838-41 (2004).

15. Design of the Women's Health Initiative clinical trial and observational study. The Women's Health Initiative Study Group. *Control Clin Trials* **19**, 61-109 (1998).

16. Shumaker, S.A. *et al.* The Women's Health Initiative Memory Study (WHIMS): a trial of the effect of estrogen therapy in preventing and slowing the progression of dementia. *Control Clin Trials* **19**, 604-21 (1998).

17. Vaidya, D. *et al.* Incidence of coronary artery disease in siblings of patients with premature coronary artery disease: 10 years of follow-up. *Am J Cardiol* **100**, 1410-5 (2007).

18. Yanek, L.R. *et al.* Hypertension among siblings of persons with premature coronary heart disease. *Hypertension* **32**, 123-8 (1998).

19. Frohlich, E.D. Recommendations for blood pressure determination by sphygmomanometry. *Ann Intern Med* **109**, 612 (1988).

20. Chobanian, A.V. *et al.* The Seventh Report of the Joint National Committee on Prevention, Detection, Evaluation, and Treatment of High Blood Pressure: the JNC 7 report. *JAMA* **289**, 2560-72 (2003).

21. Pulley, J., Clayton, E., Bernard, G.R., Roden, D.M. & Masys, D.R. Principles of human subjects protections applied in an opt-out, de-identified biobank. *Clin Transl Sci* **3**, 42-8 (2010).

22. Kayima, J. *et al.* Determinants of hypertension in a young adult Ugandan population in epidemiological transition—the MEPI-CVD survey. *BMC Public Health* **15**, 830 (2015).

23. Ehret, G.B. *et al.* Genetic variants in novel pathways influence blood pressure and cardiovascular disease risk. (2011).

24. Franceschini, N. *et al.* Genome-wide association analysis of blood-pressure traits in African-ancestry individuals reveals common associated genes in African and non-African populations. *The American Journal of Human Genetics* **93**, 545-554 (2013).

25. Zhu, X. *et al.* Combined admixture mapping and association analysis identifies a novel blood pressure genetic locus on 5p13: contributions from the CARe consortium. *Hum Mol Genet* **20**, 2285-95 (2011).

26. Zhu, X. & Cooper, R.S. Admixture mapping provides evidence of association of the VNN1 gene with hypertension. *PLoS One* **2**(2007).

27. Zhu, X. *et al.* Admixture mapping for hypertension loci with genome-scan markers. *Nat Genet* **37**, 177-81 (2005).

28. Wang, Y.-J. *et al.* The association of the vanin-1 N131S variant with blood pressure is mediated by endoplasmic reticulum-associated degradation and loss of function. *PLoS Genet* **10**, e1004641 (2014).
